# Supplementary material for: Energetics of Baird aromaticity supported by inversion of photoexcited chiral [4n]annulene derivatives
Source: Nat Commun. 2017 Aug 24;8:346. doi: 10.1038/s41467-017-00382-1 (PMC5570949; doi:10.1038/s41467-017-00382-1)
Supplement: Supplementary file 1 — Supplementary Information [file 41467_2017_382_MOESM1_ESM.pdf]

Description of Supplementary Files

File Name: Supplementary Information
Description: Supplementary Figures, Supplementary Tables, Supplementary Note, Supplementary Methods, Supplementary Discussion and Supplementary References

File Name: Peer Review File

Supplementary Methods

General

All reagents were purchased from Kanto Chemical, Tokyo Chemical Industry (TCI) and Wako Pure Chemical Industries. ^{1}H NMR and ^{13}C NMR spectra were recorded on a JEOL model JNM-ECA 500II spectrometer operating at 500 and 125 MHz, respectively. All chemical shifts (δ) are expressed in ppm. ^{1}H NMR spectra are referenced relative to the signals arising from residual non-deuterated solvent, $CHCl_3$ (δ 7.26 ppm), while ^{13}C NMR spectra are referenced relative to $CDCl_3$ (δ 77.2 ppm). Matrix-assisted laser desorption/ionization time-of-flight (MALDI-TOF) mass spectrometry was performed in reflector mode on a Bruker model Autoflex™ speed spectrometer using 9-nitroanthracene as a matrix. Column chromatography was performed on a Biotage model Isolera™ Prime flash system, using a Biotage SNAP Cartridge KP-Sil column (25–100 g; particle size 50 μm; irregular silica). Recycling preparative size exclusion chromatography (SEC) was performed on a Japan Analytical Industry (JAI) model LC908 high-performance liquid chromatograph using a column set consisting of JAIGEL 1H and 2H. Chiral HPLC was performed on a JASCO model PU-2080i HPLC pump, equipped with a JASCO model MD-2015 Plus variable-wavelength UV/Vis detector and CD-2095A CD detector with a DAICEL CHIRALPAK IF (4.6 mm-ϕ × 150 mm) or CHIRALPAK IA (4.6 mm-ϕ × 150 mm) column. Electronic absorption and circular dichroism (CD) spectra were recorded on a JASCO model V-670 UV/Vis/NIR spectrometer and a JASCO model J-820 spectrometer, respectively, equipped with a UNISOKU CoolSpeK temperature/stirring controller using a quartz cell with a 10 mm optical path length. Photoirradiation for excitation was performed using an Asahi Spectra model MAX-301 300-W xenon light source using 365 and 420 nm bandpass filters. Absolute photoluminescence quantum yields were measured with a Hamamatsu Photonics model C9920-02G absolute PL quantum yield measurement system. Single-crystal XRD was performed on a Rigaku model VariMax Dual with Saturn724+ CCD X-ray diffractometer.

Synthesis and Characterization

***β*-linked thiophene dimer (Th2)**

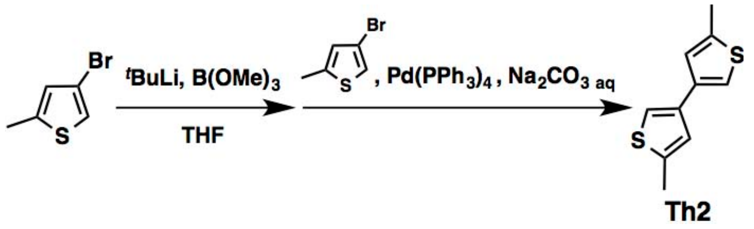

To a THF solution (200 mL) of 4-bromo-2-methylthiophene (10.0 g, 56.5 mmol) was added a hexane solution of *tert*-butyllithium (37.3 mL, 60.8 mmol) at –78 °C dropwise under Ar, and the mixture was stirred at –78 °C for 1 h. $B(OMe)_3$ (9.48 g, 91.3 mmol) was slowly added to this reaction mixture, and the resultant mixture was stirred at –78 °C for 1 h followed by 25 °C for 12 h. 4-Bromo-2-methylthiophene (10.0 g, 56.5 mmol), $Pd(PPh_3)_4$ (1.76 g, 1.52 mmol), and 2 M aqueous Na_2CO_3 (65 mL) were successively added to this reaction mixture, and the resultant mixture was refluxed for 12 h under Ar. Then, the reaction mixture was poured into aqueous NH_4Cl, and the separated aqueous layer was extracted 3 times with EtOAc. The combined organic extract was washed with brine, dried over Na_2SO_4, and evaporated to dryness under reduced pressure. The residue was subjected to column chromatography on silica gel using hexane/CH_2Cl_2 (10/1 v/v) as eluent to allow isolation of **Th2** as a white solid (8.71 g, 44.8 mmol) in 79% yield. ^{1}H NMR (500 MHz, $CDCl_3$, 25 °C): δ (ppm) 7.06 (d, J = 2.0 Hz, 2H), 6.96 (d, J = 1.0 Hz, 2H), 2.50 (d, J = 1.0 Hz, 6H); ^{13}C NMR (125 MHz, $CDCl_3$, 25 °C): δ (ppm) 140.3, 137.4, 124.7, 117.4, 15.5; High Resolution MALDI-TOF-MS: *m*/*z* 194.080 ($[M]^+$, calcd. for $C_{10}H_{10}S_2$ 194.022).

$^{Th4}COT_{Saddle}$ and $^{Th6}CDH_{Screw}$

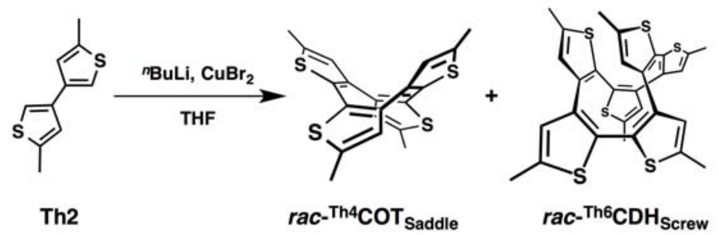

To a THF solution (120 mL) of **Th2** (1.94 g, 10 mmol) was added a hexane solution of butyllithium (9.1 mL, 24 mmol) at –78 °C dropwise under Ar, and the mixture was stirred at –78 °C for 1h and slowly warmed up to reach 25 °C in 1 h. The reaction mixture was added to

a flask with anhydrous $CuBr_2$ (7.95 g, 35.6 mmol), and the mixture was stirred for 12 h under Ar. Then, the reaction mixture was poured into a 30% aqueous ammonia solution, and the separated aqueous layer was extracted 5 times with EtOAc. The combined organic extract was washed with brine, dried over Na_2SO_4, and evaporated to dryness under reduced pressure. The residue was subjected to column chromatography on silica gel using hexane/EtOAc (10/1 v/v), followed by recycling size exclusion chromatography (SEC). The fifth fraction of SEC was collected and recrystallized from CH_2Cl_2/hexane to give a racemic mixture of $^{\mathbf{Th4}}\mathbf{COT}_{\mathbf{Saddle}}$ as a white solid (319 mg, 0.83 mmol) in 17% yield. The third fraction of SEC was collected and recrystallized from THF/hexane to give a racemic mixture of $^{\mathbf{Th6}}\mathbf{CDH}_{\mathbf{Screw}}$ as a yellow solid (119 mg, 0.21 mmol) in 6.2% yield.

$^{\mathbf{Th4}}\mathbf{COT}_{\mathbf{Saddle}}$: ^{1}H NMR (500 MHz, $CDCl_3$, 25 °C): δ (ppm) 6.57 (d, J = 1.5 Hz, 4H), 2.43 (d, J = 1.5 Hz, 12H); ^{13}C NMR (125 MHz, $CDCl_3$, 25 °C): δ (ppm) 141.0, 136.7, 130.2, 128.1, 15.5; High Resolution MALDI-TOF-MS: m/z 384.223 ($[M]^+$, calcd. for $C_{20}H_{16}S_4$ 384.014). Optical resolution of $^{\mathbf{Th4}}\mathbf{COT}_{\mathbf{Saddle}}$ was performed at 25 °C using hexane/CH_2Cl_2 (95/5 v/v) as an eluent on a DAICEL CHIRALPAK IF (4.6 mm-ϕ × 150 mm) column. The flow rate was 1.0 mL/min, and the injection amount was 5 μL (500 mg/L in hexane/CH_2Cl_2 (50/50 v/v)).

$^{\mathbf{Th6}}\mathbf{CDH}_{\mathbf{Screw}}$: ^{1}H NMR (500 MHz, $CDCl_3$, 25 °C): δ (ppm) 6.65 (d, J = 1.0 Hz, 2H), 6.35 (s, 2H), 6.08 (s, 2H), 2.48 (d, J = 1.5 Hz, 6H), 2.28 (s, 6H), 2.21 (d, J = 1.0 Hz, 6H); ^{13}C NMR (125 MHz, $CDCl_3$, 25 °C): δ (ppm) 140.0, 138.3, 138.1, 135.4, 133.5, 131.8, 130.8, 130.2, 129.2, 128.1, 127.7, 126.9, 15.5, 15.4, 15.2; High Resolution MALDI-TOF-MS: m/z 576.352 ($[M]^+$, calcd. for $C_{30}H_{24}S_6$ 576.020). Optical resolution of $^{\mathbf{Th6}}\mathbf{CDH}_{\mathbf{Screw}}$ was performed at 0 °C using hexane/EtOH (100/0.1 v/v) as an eluent on a DAICEL CHIRALPAK IA (4.6 mm-ϕ × 150 mm) column. The flow rate was 1.0 mL/min, and the injection amount was 5 μL (1000 mg/L in eluent).

NMR Spectra

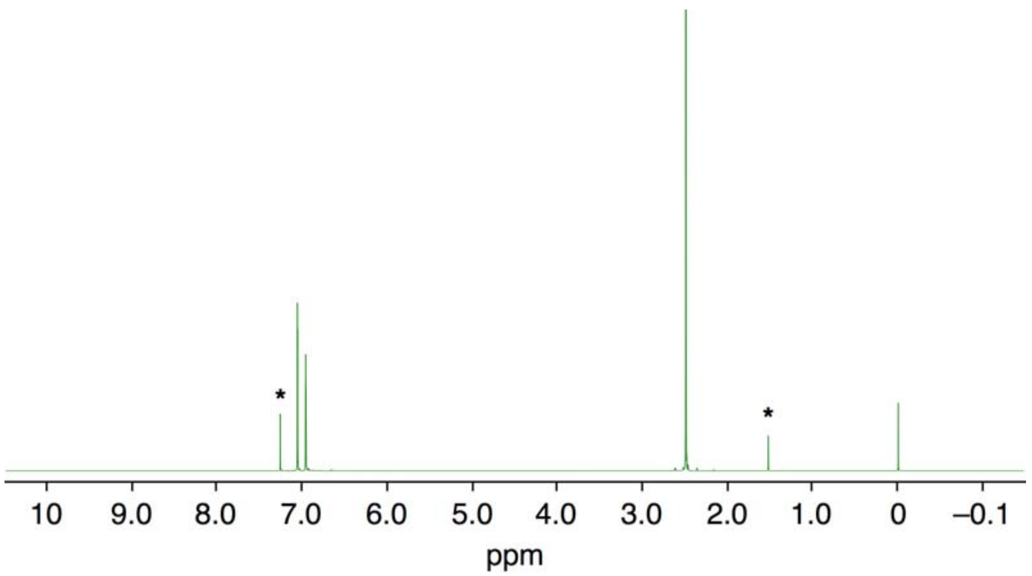

Supplementary Figure 1 | ^{1}H NMR spectrum of Th2 in $CDCl_3$ at 25 °C. Asterisked signals at δ 7.26 and 1.54 ppm are due to partially non-deuterated residues of $CDCl_3$ and water, respectively.

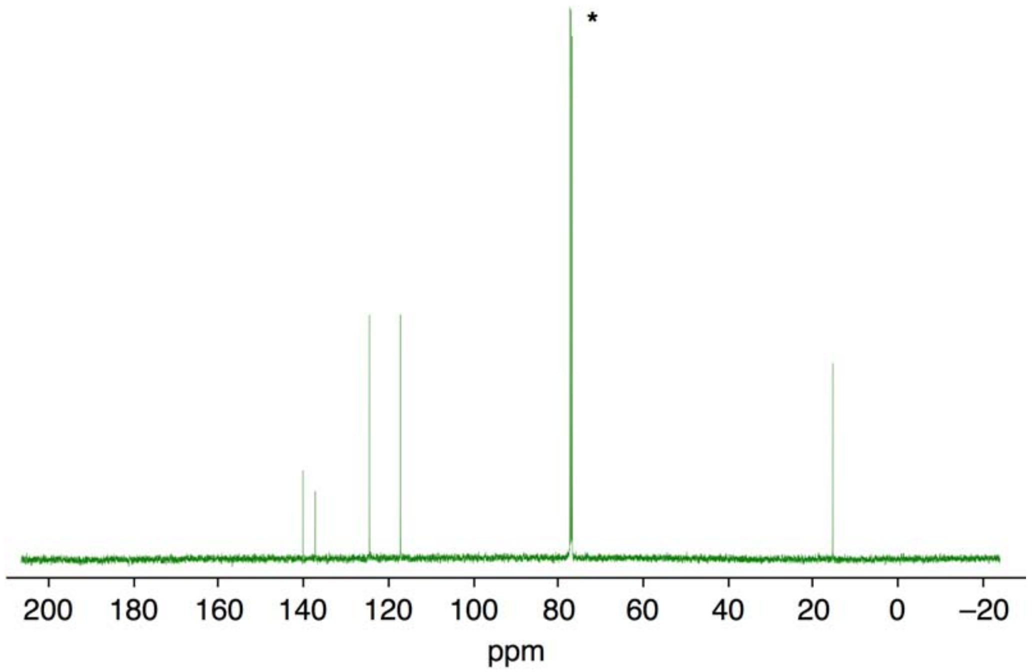

Supplementary Figure 2 | ^{13}C NMR spectrum of Th2 in $CDCl_3$ at 25 °C. Asterisked signal at δ 77.2 ppm is due to $CDCl_3$.

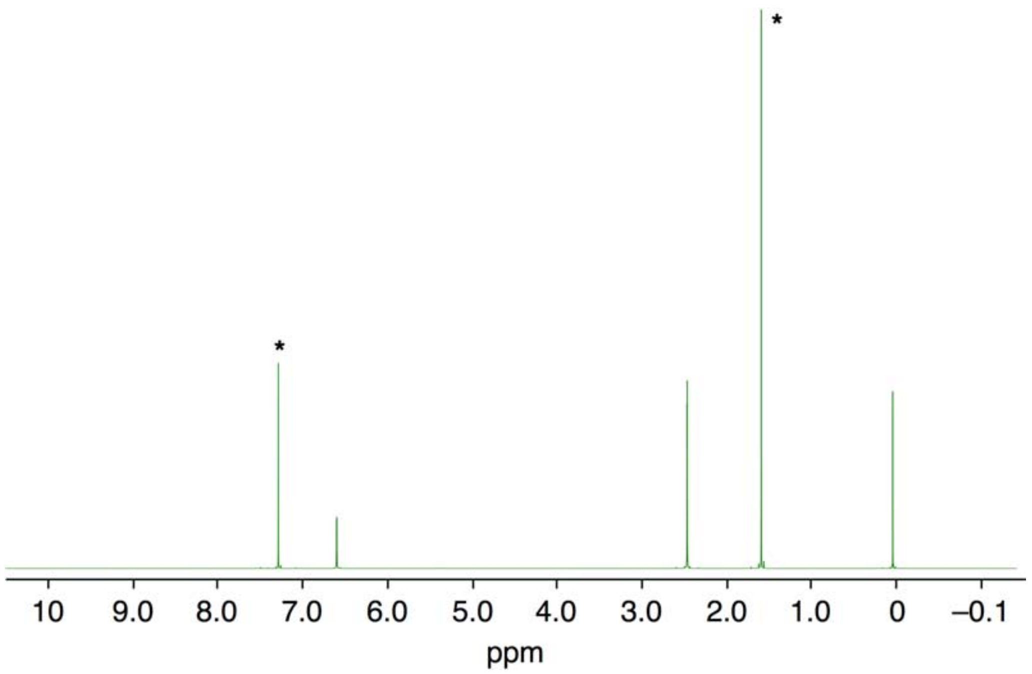

Supplementary Figure 3 | ^{1}H NMR spectrum of $^{Th4}COT_{Saddle}$ in $CDCl_3$ at 25 °C. Asterisked signals at δ 7.26 and 1.54 ppm are due to partially non-deuterated residues of $CDCl_3$ and water, respectively.

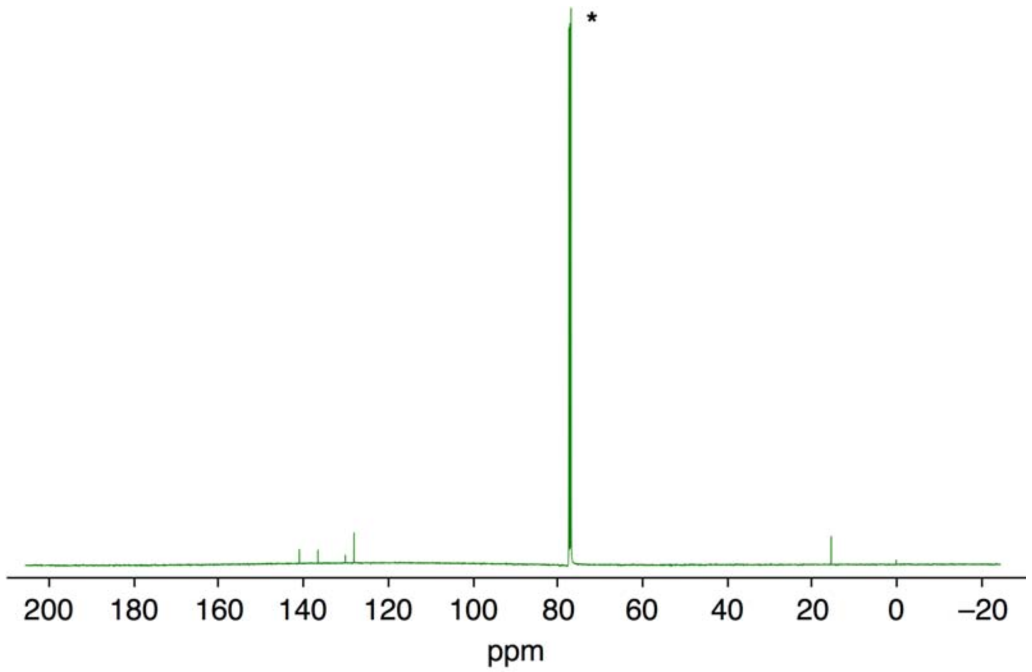

Supplementary Figure 4 | ^{13}C NMR spectrum of $^{Th4}COT_{Saddle}$ in $CDCl_3$ at 25 °C. Asterisked signal at δ 77.2 ppm is due to $CDCl_3$.

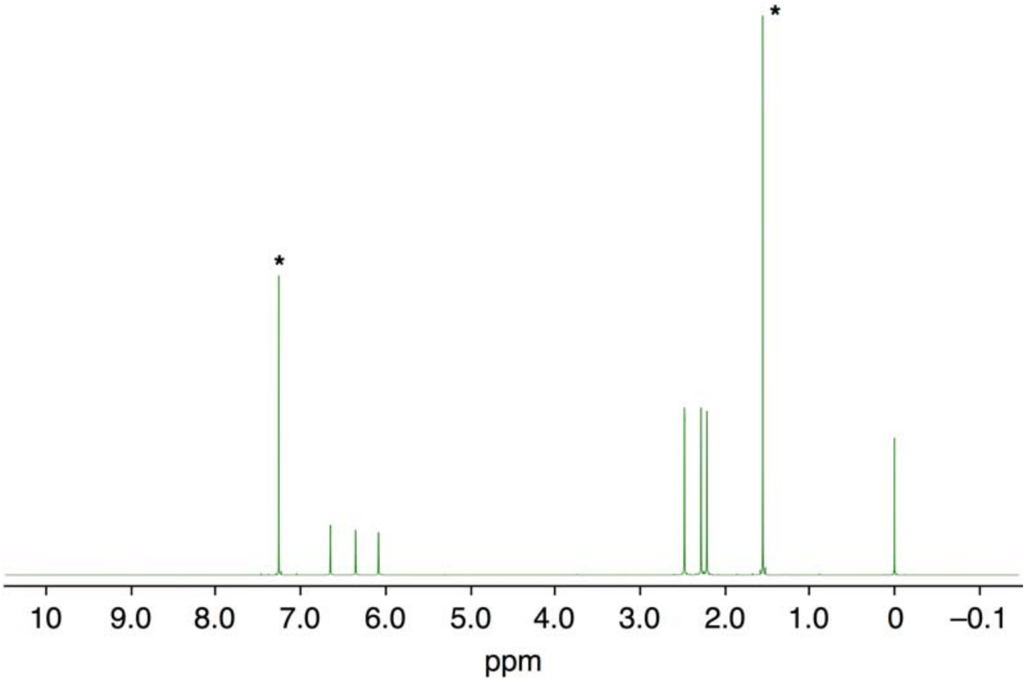

Supplementary Figure 5 | ^{1}H NMR spectrum of $^{Th6}CDH_{Screw}$ in $CDCl_3$ at 25 °C. Asterisked signals at δ 7.26 and 1.54 ppm are due to partially non-deuterated residues of $CDCl_3$ and water, respectively.

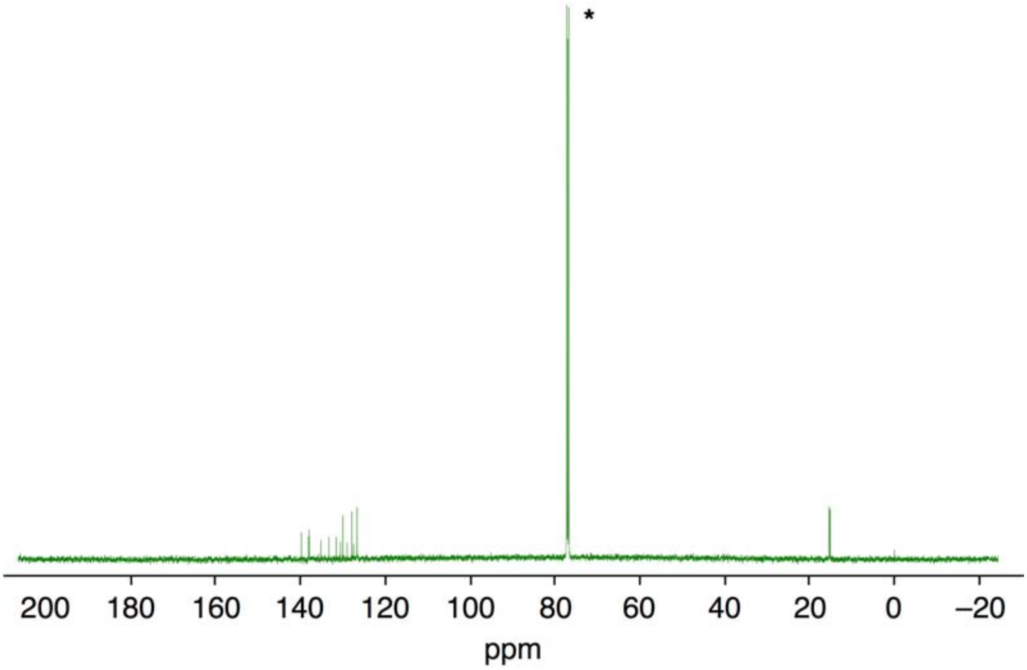

Supplementary Figure 6 | ^{13}C NMR spectrum of $^{Th6}CDH_{Screw}$ in $CDCl_3$ at 25 °C. Asterisked signal at δ 77.2 ppm is due to $CDCl_3$.

X-ray Crystallography of ^{Th4}COT$_{Saddle}$ and ^{Th6}CDH$_{Screw}$

Crystals of ^{Th4}**COT**$_{Saddle}$ and ^{Th6}**CDH**$_{Screw}$ suitable for X-ray analysis were obtained by slow diffusion of ethanol into a $CHCl_3$ solution of ^{Th4}**COT**$_{Saddle}$, slow diffusion of hexane into a THF solution of ^{Th6}**CDH**$_{Screw}$, respectively. CCDC 1526430 and CCDC 1526429 contain the supplementary data for this paper. These data can be obtained free of charge from The Cambridge Crystallographic Data Centre via www.ccdc.ac.uk/data_request-cif.

Supplementary Table 1 | Crystal data and structure refinement of ^{Th4}COT$_{Saddle}$

	^{Th4}**COT**$_{Saddle}$
Empirical formula	$C_{20}H_{16}S_4$
Formula weight	384.57
Temperature (K)	93
Wavelength (Å)	0.71069
Crystal system	triclinic
Space group	*P*–1
a (Å)	7.8532(19)
b (Å)	10.950(3)
c (Å)	11.469(3)
α (deg)	76.581(13)
β (deg)	75.213(14)
γ (deg)	69.559(12)
Volume (Å^3)	882.4(4)
Z	2
Calculated density, (g/cm^3)	1.447
Absorption coefficient (mm^{-1})	0.537
F(000)	400
Crystal size (mm)	0.05 × 0.06 × 0.07
θ range for data collection (deg)	2.514 to 24.997
Index ranges	–9≤h≤9, –12≤k≤13, –13≤l≤13
Reflections collected	5970
Independent reflections	3052 [*R*(int) = 0.0269]
Absorption correction	Multi-scan
Refinement method	Full-matrix least-squares on F^2
Data / restraints / parameters	3052 / 0 / 221
Goodness-of-fit on F^2	1.040
Final *R* indices [I > 2σ(I)]	R_1 = 0.0350, wR_2 = 0.0795
Largest diff. peak and hole (e Å^{-3})	0.300 and –0.305

Supplementary Table 2 | Crystal data and structure refinement of $^{Th6}CDH_{Screw}$

	$^{Th6}CDH_{Screw}$
Empirical formula	$C_{30}H_{24}S_6$
Formula weight	576.85
Temperature (K)	93
Wavelength (Å)	0.71075
Crystal system	monoclinic
Space group	*C*2/*c*
a (Å)	39.974(8)
b (Å)	9.0297(16)
c (Å)	16.077(3)
α (deg)	90
β (deg)	109.501(3)
γ (deg)	90
Volume (Å^3)	5470.2(18)
Z	8
Calculated density, (g/cm^3)	1.401
Absorption coefficient (mm^{-1})	0.519
F(000)	2400
Crystal size (mm)	0.30 × 0.15 × 0.10
θ range for data collection (deg)	2.319 to 24.999
Index ranges	$-47 \le h \le 47, -10 \le k \le 10, -19 \le l \le 17$
Reflections collected	17714
Independent reflections	4805 [*R*(int) = 0.0304]
Absorption correction	Multi-scan
Refinement method	Full-matrix least-squares on F^2
Data / restraints / parameters	4805 / 0 / 331
Goodness-of-fit on F^2	1.254
Final *R* indices [$I > 2\sigma(I)$]	$R_1 = 0.0462$, $wR_2 = 0.0994$
Largest diff. peak and hole (e Å^{-3})	0.324 and –0.362

Correction Methods for Kinetic Studies under Photoirradiation

All the kinetic studies for the chiral inversion processes under photoexcitation conditions were conducted at a temperature where the decay profiles of the CD intensity were negligibly small without photoirradiation. Since the CD spectra cannot be measured during photoirradiation, we alternately performed irradiation (250 s, red vertical lines in Supplementary Fig. 7a) and CD spectroscopic measurement (50 s, black dots are the CD intensity at a certain wavelength). Under the irradiation conditions, we can extrapolate the time-dependent CD intensity decay profiles drawn as grey solid lines in Supplementary Fig. 7a. When performing kinetic studies using the Eyring equation, we corrected the experimental time to be the total irradiation time and used the plot for kinetic analysis (Supplementary Fig. 7b).

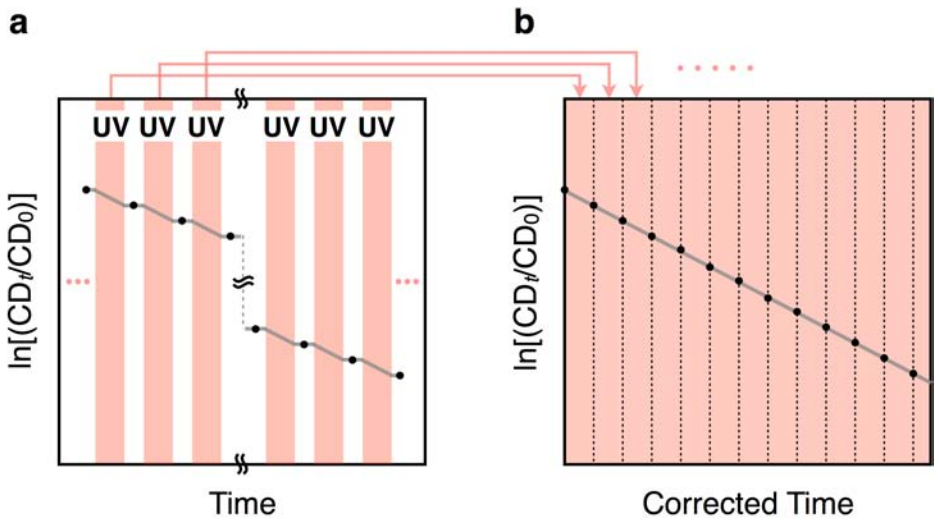

Supplementary Figure 7 | Illustration of the correction method for kinetic analysis. **a,** Illustration of the CD intensity decay profile under photoirradiation. **b,** Illustration of the CD intensity decay profile after correction by removing the time during the CD spectroscopic measurement under dark.

General Methods for Transient Absorption Spectroscopy (TAS)

Femtosecond Transient Absorption. The femtosecond time-resolved transient absorption (fs-TA) spectrometer consists of Optical Parametric Amplifiers (Palitra, Quantronix) pumped by a Ti:sapphire regenerative amplifier system (Integra-C, Quantronix) operating at 1 kHz repetition rate and an optical detection system. The generated OPA pulses, which were used as pump pulses, had a pulse width of ~ 100 fs and an average power of 100 mW in the range 280–2700 nm. White light continuum (WLC) probe pulses were generated using a sapphire window (3 mm of thickness) by focusing of small portion of the fundamental 800 nm pulses, which was picked off a quartz plate before entering to the OPA. The time delay between the pump and probe beams was carefully controlled by making the pump beam travel along a variable optical delay (ILS250, Newport). The intensities of the spectrally dispersed WLC probe pulses were monitored by a High Speed spectrometer (Ultrafast Systems). To obtain the time-resolved transient absorption difference signal (ΔA) at a specific time, the pump pulses were chopped at 500 Hz, and the absorption spectra intensities were saved alternately with or without the pump pulse. Typically, 4000 pulses excited samples to obtain the fs-TA spectra at a particular delay time. The polarization angle between the pump and probe beam was set to the magic angle (54.7°) using a Glan-laser polarizer with a half-wave retarder to prevent polarization-dependent signals. Cross-correlation FWHM in the pump-probe experiments was less than 200 fs, and the chirp of the WLC probe pulses was 800 fs in the 400–800 nm region. To minimize chirp, all reflection optics in the probe beam path and a 2 mm path length quartz cell were used. The three-dimensional data sets of ΔA versus time and wavelength were subjected to singular value decomposition and global fitting to obtain the kinetic time constants and their associated spectra using Surface Xplorer software (Ultrafast Systems). After fluorescence and TA experiments, we carefully checked the absorption spectra of all compounds to avoid artefacts from the degradation and photo-oxidation of samples. HPLC grade solvents were used for all steady-state and time-resolved spectroscopic studies.

Nanosecond Transient Absorption. The nanosecond transient absorption spectra were obtained using nanosecond flash photolysis techniques. Specifically, a tunable excitation pulse was generated using an Optical Parametric Oscillator system (Continuum, Surelite OPO), which was pumped by 355 nm from the third-harmonic output of a Q-switched Nd:YAG laser (Continuum, Surelite II-10). The time duration of the excitation pulse was ca. 6 ns, and the

pulse energy was ca. 2 mJ/pulse. A CW Xe lamp (150 W) was used as the probe light source for the transient absorption measurement. The probe light was collimated on the sample cell and was spectrally resolved using a 15 cm monochromator (Acton Research, SP150) equipped with a 600 grooves/mm grating after passing the sample. The spectral resolution was approximately 3 nm for the transient absorption experiment. The light signal was detected using an avalanche photodiode (APD). The output signal from the APD was recorded using a 500 MHz digital storage oscilloscope (Lecroy, WaveRunner 6050A) for the temporal profile measurement. Since the triplet-state dynamics of molecules in solution are strongly dependent on the concentration of oxygen molecules dissolved in solution, we attempted to remove oxygen by degassing with Ar gas for 15 min.

Determination of the Lifetimes of the Excited Species

The lifetime of ^{Th4}**COT**$_{Saddle}$ in the S_1 state was determined to be 5.5 ps by fs-TAS (Supplementary Fig. 16), where the decay profile was fitted with a single exponential. The lifetime of ^{Th4}**COT**$_{Saddle}$ in the T_1 state was determined by fs-TAS (Supplementary Fig. 22) and ns-TAS (Supplementary Fig. 23) under sensitized conditions using fluorenone as a triplet sensitizer. Under these conditions, three excited species with lifetimes of 5.5 ps, 140 ps and 2.0 μs were detected (Supplementary Figs. 22 and 23). The excited species with a lifetime of 5.5 ps was assigned to the S_1 state of ^{Th4}**COT**$_{Saddle}$. Considering that the lifetimes of the S_1 and T_1 states of fluorenone are 140 ps and 7.2 μs, respectively (determined by fs-TAS and ns-TAS in Supplementary Figs. 20 and 21), the observed excited species with a lifetime of 140 ps was assigned to the S_1 state of fluorenone. Therefore, the remaining excited species with a lifetime of 2.0 μs was assigned to the T_1 state of ^{Th4}**COT**$_{Saddle}$. This is supported by the calculated triplet-triplet absorption spectrum of ^{Th4}**COT**$_{Saddle}$, which exhibited a similar absorption band to the ns-TAS at approximately 600 nm (Supplementary Fig. 48).

The fs-TAS and ns-TAS of ^{Th6}**CDH**$_{Screw}$ revealed that both the S_1 and T_1 states were generated when excited with 355 nm light, with lifetimes of 70 ps and 350 ns, respectively (Supplementary Figs. 18 and 19). This was further confirmed by the fs-TAS and ns-TAS in the presence of the triplet sensitizer fluorenone (Supplementary Figs. 24 and 25). Under the sensitized conditions, excited species with lifetimes of 70 ps and 350 ns were also observed, with a larger population of the latter species. Another excited species with a lifetime of 2.0 μs was determined to be the T_1 state of fluorenone. The shorter lifetime compared to that obtained from Supplementary Fig. 21 (7.2 μs) is probably due to partial quenching by the energy transfer to ^{Th6}**CDH**$_{Screw}$.

For the TAS in sensitized conditions, 10 equiv. of fluorenone was required to detect a signal in the spectrum. In addition, both the 355 and 420 nm photoexcitations were employed for the TAS in the sensitized conditions because the photoexcitation at 420 nm, where the isomerization reactions of ^{Th4}**COT**$_{Saddle}$ and ^{Th6}**CDH**$_{Screw}$ in sensitized conditions were performed, cannot always generate a TA signal of sufficient intensity. We believe that photoexcitation at 355 nm in the presence of fluorenone not only works for exciting fluorenone to generate the T_1 state of ^{Th4}**COT**$_{Saddle}$ or ^{Th6}**CDH**$_{Screw,}$ but also works for exciting ^{Th4}**COT**$_{Saddle}$ or ^{Th6}**CDH**$_{Screw}$ directly to the S_1 state.

Supplementary Table 3 | List of lifetimes of ^{Th4}COT$_{Saddle}$ and ^{Th6}CDH$_{Screw}$ in the S_1 and T_1 states based on TAS.

	^{Th4}**COT**$_{Saddle}$	^{Th6}**CDH**$_{Screw}$	Fluorenone
S_1 state	5.5 ps	70 ps	140 ps
T_1 state	2.0 μs	350 ns	7.2 μs

General Calculation Methods

DFT calculations. DFT calculations were performed with B3LYP and the 6-31G and 6-311G Pople basis set families[1,2]. Structures in the S_0 and T_1 states were optimized using B3LYP-D3(BJ)/6-31G(d), and single-point calculations with B3LYP-D3(BJ)/6-311+G(d,p) were performed on these geometries. Corrections to the enthalpy were taken from the frequency calculations at the B3LYP-D3(BJ)/6-31G(d) level. Transition state structures were found using the QST3 and Berny optimization methods in Gaussian[3], and their identities were confirmed by frequency calculations and IRC analysis. We checked for possible broken-symmetry UDFT solutions using the keyword stable=opt in Gaussian and found none for the structures considered. Single-point calculations with M06-2X[4] and B2PLYP[5] augmented with dispersion correction were also employed.

TD-DFT calculations. TD-DFT calculations were performed using TD-B3LYP-D3(BJ)/6-31+G(d,p) with optimization of the lowest excited state. Minimum structures were confirmed by numerical frequency calculations. Transition states were found with the QST3 method in Gaussian, starting from the minimum energy structures, and were confirmed by frequency calculations to have exactly one negative frequency corresponding to chiral interconversion. Minor perturbation along this normal mode in both directions and subsequent geometry optimization led to reactants and products, respectively. The saddle points in the S_1 state were found using TD-B3LYP-D3(BJ)/6-31+G(d,p) and Gaussian 16 Rev. A.03[6]

NICS calculations. NICS scan calculations were performed with Gaussian in conjunction with the Aroma 1.0 package[7–10] using B3LYP/6-311+G(d,p) and the GIAO procedure[11]. Single point $NICS(1)_{iso}$ calculations were used in some instances due to it being more readily available. Special integration grids were used by applying the keyword cphf=grid=fine integral=grid=ultrafine in Gaussian.

For the NICS scans in the S_1 state, we used state-specific CASSCF[12] calculations using Dalton2016.0[13] with an active space of eight electrons in eight orbitals (8in8) based on the optimized DFT geometries. The active space was identical to that given below in Supplementary Fig. 26. Calculations were conducted using the 6-31++G(d,p) basis set, which includes both diffuse functions and polarization functions. Due to the large size of the system, we could not use any larger basis set.

ACID calculations. ACID plots were generated with ACID 2.0.0 from Gaussian calculations using B3LYP-6-311+G(d,p) and CSGT[14]. Special integration grids were used by applying the keywords cphf=grid=fine and integral=grid=ultrafine in Gaussian. A density of points of ca. 40/$Å^3$ was used for the ACID plots.

DLNPO-CCSD(T) calculations. DLPNO-CCSD(T)[15,16] single-point energy calculations were performed with Orca 3.0.3[17] and the cc-pVTZ basis set[18,19] on the geometries from B3LYP-D3(BJ)/6-31G(d). Corrections to the enthalpy were taken from the DFT frequency calculations. DLPNO-CCSD(T) calculations approximate full CCSD(T) by 1 kcal mol^{-1} or better and represent possibly the best *ab initio* method that is generally applicable to molecules of this size[20].

CASSCF/CASPT2 calculations. CASSCF calculations[12] were performed for $^{\mathbf{Th4}}\mathbf{COT}_{\mathbf{Saddle}}$ with an active space of 8 electrons in 8 orbitals and the 6-31G(d) basis set using a development version of Molcas 8.1[21]. The active space orbitals correspond to the 8 orbitals of the COT core (Supplementary Fig. 26). A state-average procedure with the three lowest roots of singlet multiplicity was employed. CASPT2 single-point calculations were performed using an imaginary shift of 0.2 a.u. and a multi-state procedure with the same three roots as in the CASSCF calculation[22,23].

The S_1 transition state was optimized at the CASSCF level (where it is the third root due to the lack of dynamic correlation) using the saddle method with the keyword RP-Coordinates[24]. The approximate Hessian generated from this procedure displays two negative frequencies, one corresponding to the inversion coordinate and one associated with methyl rotation. Because no dispersion forces are accounted for in CASSCF, the barrier for methyl rotation is usually very small; therefore, negative frequencies corresponding to these rotations often occur. As this negative frequency is not important to the inversion process, it can be disregarded for the purposes of transition state structure validation.

Activation Energy for $^{Th4}COT_{Saddle}$ with High-level Methods

Due to the overestimation by B3LYP-D3(BJ)/6-311+G(d,p), giving an activation enthalpy of 29.9 kcal mol^{-1} compared to the experimental value of 25.4 ± 0.8 kcal mol^{-1}, we performed calculations with the high-level DLPNO-CCSD(T) method and the cc-pVTZ basis set and the M06-2X and B2PLYP density functions, which have known accuracy for thermochemistry. Corrections to the enthalpy were taken from the frequency calculations at the B3LYP-D3(BJ)/6-31G(d) level. The results given in Supplementary Table 4 lie in the range 28–33 kcal mol^{-1}, higher than the experimental value of 25.4 ± 0.8 kcal mol^{-1}. The reason for the discrepancy of ca. 3-4 kcal mol^{-1} between the calculated values and experiment is not clear but may be due to the limited basis set and/or solvent effects.

Supplementary Table 4 | Calculated activation enthalpies for $^{Th4}COT_{Saddle}$ compared to experiments using various methods.

Method	$\Delta H^{‡}$ (kcal mol^{-1})
B3LYP-D3(BJ)/6-31G(d)	28.8
B3LYP-D3(BJ)/6-311+G(d,p)	29.9
M06-2X-D3/6-311+G(d,p)	30.6
B2PLYP-D3(BJ)/6-311+G(d,p)	33.1
DLPNO-CCSD(T)/cc-pVTZ	28.7
Exp.	25.4 ± 0.8

For the parent COT molecule, the computed activation enthalpies are in the range 11.2–13.2 kcal mol^{-1}, very close to the experimental value of 12.7 ± 0.5 kcal mol^{-1} (Supplementary Table 5)[25]. This good agreement between the theory and experiment in the case of the parent COT and only slight overestimation in the case of $^{Th4}COT_{Saddle}$ makes us confident in the computational data for these compounds.

Supplementary Table 5 | Calculated activation enthalpies for parent COT compared to experiments using various methods.

Method	$\Delta H^{‡}$ (kcal mol^{-1})
B3LYP-D3(BJ)/6-31G(d)	11.2
B3LYP-D3(BJ)/cc-pVTZ	11.8
M06-2X-D3/cc-pVTZ	14.4
B2PLYP-D3(BJ)/cc-pVTZ	12.4
DLPNO-CCSD(T)/cc-pVTZ	13.2
CCSD(T)/cc-pVTZ	12.7
Exp.[25]	12.7 ± 0.5

S_1 Transition State for ^{Th4}COT$_{Saddle}$ at the CASSCF(8in8) Level

To verify the validity of the calculated TD-DFT S_1 transition state, we performed benchmark calculations with CASSCF/6-31G(d). This is important because the parent COT molecule displays two singlet excited states which lie very close in energy to each other: one HOMO –> LUMO singly excited (SE) state and one $HOMO^2$ –> $LUMO^2$ doubly excited (DE) state[26]. For the parent molecule, the DE state is the lowest energy state at the D_{8h} singlet state minimum geometry and crosses the SE state along the relaxation from D_{2d} to D_{8h}. However, in the case of ^{Th4}**COT**$_{Saddle}$, the SE state is consistently lower than the DE state along the interconversion of the two chiral S_1 minima over the S_1 transition state. This makes it possible to study this reaction using TD-DFT, which would not be possible if the DE state was lower, as doubly excited states cannot be treated with conventional TD-DFT. CASSCF using the RP-Coordinates saddle method found a more planar but qualitatively similar transition state structure compared to TD-DFT. The saddle method also provides a pseudo-IRC that we used for single-point calculations with TD-B3LYP-D3(BJ) and MS-CASPT2 (Supplementary Fig. 27). The activation energies with MS-CASPT2 and TD-B3LYP-D3(BJ) are higher than that calculated for the TD-B3LYP-D3(BJ) transition state, indicating that dynamic correlation is important for finding the correct transition state structure. The S_2 state also exhibits a barrier for inversion and is separated from the S_1 state by 10–20 kcal mol^{-1} along the whole inversion process. In summary, the *ab initio* CASSCF calculations of the S_1 inversion barrier of ^{Th4}**COT**$_{Saddle}$ validate the results from TD-B3LYP-D3(BJ) and that the activation energy and transition state structure is probably better calculated with TD-B3LYP-D3(BJ) (with inclusion of dynamical correlation) because the activation energy with the CASSCF transition state structure is too large compared to the experiment.

Supplementary Discussion

Ring Inversion Kinetic Studies

The observed decay profiles of $^{\mathbf{Th4}}\mathbf{COT}_{\mathbf{Saddle}}$ and $^{\mathbf{Th6}}\mathbf{CDH}_{\mathbf{Screw}}$ both satisfied first-order kinetics, where the rate constants of racemization (k_{rac}) and ring inversion (k_{inv}) were obtained from Supplementary Equation (1):

$$\ln\left(\frac{\mathrm{CD}_t}{\mathrm{CD}_0}\right) = -k_{\mathrm{rac}}t = -2k_{\mathrm{inv}}t \quad (1)$$

The half-life ($\tau_{1/2}$) of the CD intensity was obtained from Supplementary Equation (2):

$$\tau_{1/2} = \frac{\ln 2}{k_{\mathrm{rac}}} = \frac{\ln 2}{2k_{\mathrm{inv}}} = \frac{0.693}{2k_{\mathrm{inv}}} \quad (2)$$

The k_{inv} values obtained at various temperatures were plotted and analysed using the Eyring equation, Supplementary Equation (3):

$$\ln\left(\frac{k_{\mathrm{inv}}}{T}\right) = -\frac{\Delta H^{\ddagger}}{R}\cdot\frac{1}{T} + \ln\left(\frac{k_{\mathrm{B}}}{h}\right) + \frac{\Delta S^{\ddagger}}{R} \quad (3)$$

where T, $\Delta H^{\ddagger}$, R, k_B, h and $\Delta S^{\ddagger}$ are the absolute temperature, activation enthalpy, gas constant, Boltzmann constant, Planck's constant and activation entropy, respectively. By plotting $\ln(k_{inv}/T)$ versus $1/T$, we obtained $\Delta H^{\ddagger}$ and $\Delta S^{\ddagger}$ from the slope and y-intercept, respectively.

The Eyring plots for the ring inversion of $^{\mathbf{Th4}}\mathbf{COT}_{\mathbf{Saddle}}$ and $^{\mathbf{Th6}}\mathbf{CDH}_{\mathbf{Screw}}$ in the dark are shown in Supplementary Fig. 17.

Calculation of k_{inv} under Photoirradiation

The calculation of inversion kinetics under photoirradiation requires special attention. First, one must consider the measurement time. Since the CD intensity decays only under photoexcitation conditions, the measurement time was corrected according to the procedure shown in Supplementary Fig. 11. The second factor to consider is the rate constant (k_{inv}). Because photoirradiation cannot excite all the molecules in the system simultaneously, the observed rate constant (k_{obs}) is not an intrinsic value of the molecule (k_{inv}) but an apparent value. Thus, k_{obs} satisfies the following Supplementary Equation (4):

$$k_{\mathrm{obs}} = k_{\mathrm{inv}} \frac{[\mathrm{M}^*]}{[\mathrm{M}]}$$

$$\Leftrightarrow \qquad k_{\mathrm{inv}} = \frac{k_{\mathrm{obs}}[\mathrm{M}]}{[\mathrm{M}^*]} \tag{4}$$

Since the experimental set-ups for photoirradiation were identical within a series of experiment for each Eyring plot, we can assume that the concentration of the substrate in the excited state ([M*]) is constant. By inserting Supplementary Equation (4) into Supplementary Equation (3), we obtain

$$\ln\left(\frac{k_{\mathrm{obs}}[\mathrm{M}]}{[\mathrm{M}^*]T}\right) = -\frac{\Delta H^{\ddagger}}{R}\cdot\frac{1}{T} + \ln\left(\frac{k_{\mathrm{B}}}{h}\right) + \frac{\Delta S^{\ddagger}}{\mathrm{R}}$$

$$\Leftrightarrow \qquad \ln\left(\frac{k_{\mathrm{obs}}}{T}\right) = -\frac{\Delta H^{\ddagger}}{R}\cdot\frac{1}{T} + \ln\left(\frac{k_{\mathrm{B}}}{h}\right) + \frac{\Delta S^{\ddagger}}{R} + \ln\left(\frac{[\mathrm{M}^*]}{[\mathrm{M}]}\right) \tag{5}$$

Therefore, by plotting ln (k_{obs}/T) versus $1/T$, we can obtain $\Delta H^{\ddagger}$ from the slope. However, we cannot obtain the activation entropy ($\Delta S^{\ddagger}$) since [M*]/[M] cannot be determined in our experimental set-ups.

The Eyring plot for the ring inversion of $^{\mathbf{Th4}}\mathbf{COT}_{\mathbf{Saddle}}$ under photoirradiation (λ = 365 ± 5 nm) is shown in Supplementary Fig. 18.

In the case of the photosensitization conditions, the triplet excited state of the substrate (^{3}M*) is generated through multiple electronic transition events, including a photoexcitation of the sensitizer and an intersystem crossing, followed by a bimolecular energy transfer from the sensitizer to the substrate M. These processes are known to be temperature dependent[27] and thus [^{3}M*] should also be temperature dependent. Therefore, the activation enthalpy obtained by using Supplementary Equation (5) under photosensitized conditions should intrinsically include some artefact derived from temperature. In order to minimize the effect of temperature on [^{3}M*], we collected the CD intensity decay profiles within a relatively narrow temperature range (0, 5, 10, 15 and 20 °C; Supplementary Fig. 19). A fairly good coefficient of determination (R^2 = 0.9270) was obtained for the Eyring plot (Supplementary Fig. 19c). To determine whether the temperature effect is small within the range we investigated, we compared the activation energies obtained using the data points at a lower temperature range (0, 5 and 10 °C), then at a higher temperature range (10, 15 and 20 °C) and then using the full temperature range (0, 5, 10, 15 and 20 °C). All the values were within the range of 2.4 ± 0.14 kcal mol^{-1} indicating the small effect of temperature on the activation enthalpy.

Exclusion of the Possibility of Photothermal Activation of Ring Inversion

An alternative hypothesis for the photo-acceleration of the ring inversion of $^{\mathbf{Th4}}\mathbf{COT}_{\mathbf{Saddle}}$ is thermal activation of molecular motion by absorbing photon energy. If photothermal activation plays a dominant role, the effect of photoirradiation on $^{\mathbf{Th6}}\mathbf{CDH}_{\mathbf{Screw}}$ should be much higher than that on $^{\mathbf{Th4}}\mathbf{COT}_{\mathbf{Saddle}}$ because the absorptivity of $^{\mathbf{Th6}}\mathbf{CDH}_{\mathbf{Screw}}$ at λ = 365 nm (ε = 9.3 × 10^3 cm^{-1} M^{-1}, Fig. 2d) is 7.2 times greater than that of $^{\mathbf{Th4}}\mathbf{COT}_{\mathbf{Saddle}}$ (ε = 1.3 × 10^3 cm^{-1} M^{-1}, Fig. 2c) and both compounds have low luminescence quantum yields ($^{\mathbf{Th4}}\mathbf{COT}_{\mathbf{saddle}}$: ϕ_{FL} = 0.000, $^{\mathbf{Th6}}\mathbf{CDH}_{\mathbf{Screw}}$: ϕ_{FL} = 0.001 at λ_{ex} = 365 nm). Although these photophysical properties suggest that $^{\mathbf{Th6}}\mathbf{CDH}_{\mathbf{Screw}}$ should have a much higher photothermal effect than $^{\mathbf{Th4}}\mathbf{COT}_{\mathbf{Saddle}}$, inversion rate enhancement was only observed in the case of $^{\mathbf{Th4}}\mathbf{COT}_{\mathbf{Saddle}}$. Therefore, photoenhancement of the ring inversion of $^{\mathbf{Th4}}\mathbf{COT}_{\mathbf{Saddle}}$ was a consequence of a photochemical process, not a photothermal process.

ACID Plots and NICS Values of ^{Th4}COT$_{Saddle}$ in the S_0 and T_1 States

The ACID plots indicate that the cyclooctatetraene (COT) ring is non-aromatic in the S_0 saddle-shaped minimum energy structure (Supplementary Fig. 33) but antiaromatic in the transition state (Supplementary Fig. 34). The thiophene units also lose part of their aromaticity. For the T_1 state, the ACID plots suggest that the saddle-shaped minimum energy structure has aromaticity in both the thiophene ring and the COT ring (Supplementary Fig. 35). This current is delocalized as a 16π-electron current. In the transition state structure, the aromaticity of the COT unit is more pronounced and that of the thiophene units decreases (Supplementary Fig. 36).

The NICS scans of the NICS$_{zz}$ values with distance from the COT ring centre are consistent with the ACID plots. In the S_0 state, the COT ring of **^{Th4}COT$_{Saddle}$** is non-aromatic at the minimum energy structure (Supplementary Fig. 37) and antiaromatic at the transition state structure (Fig. 5c). In the T_1 state, it can be concluded that both the minimum energy and transition state structure have aromatic tendency (Supplementary Fig. 37 and Fig. 5c).

The NICS scans in the S_1 state are given for the S_1 minimum energy structure (Supplementary Fig. 38) and the S_1 transition state structure (Supplementary Fig. 39). The profiles display the tycal aromatic minimum in NICS$_{zz}$, while the values are much larger (ca –180 ppm for the S_1 transition state and ca. –560 ppm for the S_1 minimum) than typically observed for the ground state (NICS(1)$_{zz}$ of benzene, which is –29.2 ppm at the B3LYP/6-311+G(d,p) level. Until the reason for these large values is identified, we hesitate to use the S_1 NICS values as anything other than qualitative indicators of aromaticity that have to be matched with other aromaticity indices, such as geometry.

Energetics of the Planar Conformation of [12]annulene

To confirm that $^{\mathbf{Th6}}\mathbf{CDH}_{\mathbf{Screw}}$ cannot take a planar conformation, energy calculation of [12]annulene in its C_2-symmetric conformation (similar to the minimum energy structure of $^{\mathbf{Th6}}\mathbf{CDH}_{\mathbf{Screw}}$) and D_{12h}-symmetric conformation (hypothetical planar conformation) was performed. The planar conformation is enormously higher in energy ($\Delta E = 106.3$, $\Delta H = 114.8$, $\Delta G = 110.9$ kcal mol^{-1}), which indicates the absence of the planar conformation in $^{\mathbf{Th6}}\mathbf{CDH}_{\mathbf{Screw}}$ conformers (Supplementary Fig. 46).

Triplet Energies of Fluorenone, ^{Th4}COT$_{Saddle}$ and ^{Th6}CDH$_{Screw}$

For efficient photosensitization, the triplet state energy (adiabatic) of the photosensitizer should be slightly higher than the triplet state energy (adiabatic) of the substrates. As shown in Supplementary Table 6, fluorenone, the sensitizer used in this study, has a ca. 5–10 kcal mol^{-1} higher triplet state energy than those of **^{Th4}COT$_{Saddle}$** and **^{Th6}CDH$_{Screw}$**, which is reasonable for smooth photosensitization.

Supplementary Table 6 | Energetics of fluorenone, ^{Th4}COT$_{Saddle}$ and ^{Th6}CDH$_{Screw}$ in the T_1 state.

	T_1 vertical (kcal mol^{-1})	T_1 adiabatic (kcal mol^{-1})	T_1 transition state (kcal mol^{-1})
**^{Th4}COT$_{Saddle}$**[§]	61.3	42.9	52.3
**^{Th6}CDH$_{Screw}$**[§]	62.2	46.6	70.1
Fluorenone (exp.)	–	53.3[*], 50.9[†,‡]	–
Fluorenone (calc.)[§]	62.5	52.6	–

[*]In methylcyclohexane/isopentane (5:1) at 77 K[28]. [†]In methylcyclohexane at 298 K[29]. [‡]Single-crystalline state at 77 K[30]. [§]Calculated value at the B3LYP/6-311+G(d,p) level.

Detailed Analysis of the Ring Inversion Transition State of ^{Th4}COT$_{Saddle}$

For the structural transition of COT, a possibility of pseudorotation[31] (Supplementary Fig. 49), other than going through planar transition state, was significantly discussed around the 1990s for the ring inversion and bond shifting. The process of ring inversion from one tub conformer to its mirror image proceeds through an intermediate tub conformer via two pseudorotation transition states. For the parent COT, it is now widely accepted that the structural transition instead occurs through a planar transition state in the ground state and not through pseudorotation[32,33]. However, in some COT derivatives with bulky substituents, pseudorotation pathways for structural transition from one tub conformation to the other one have been reported[34,35]. Also for the excited state of parent COT, the lowest-energy conical intersection of COT reachable from the S_1 state (12 kcal mol^{-1} above the planar D_{8h} minimum that could also relax to either tub conformer) has a structure similar to a pseudorotation TS[36].

For our system, the ring inversion process for **^{Th4}COT$_{Saddle}$** does not go through pseudorotation as the intermediate tub conformation would be destabilized due to co-planar thiophene rings (Supplementary Fig. 50). We have not been able to optimize any such intermediate by calculation. However, we think that the inversion of **^{Th4}COT$_{Saddle}$** borrows some of the features of the pseudorotation pathway as evidenced by the not perfectly planar conformations of the transition states in S_0, T_1 and S_1 states (Fig. 4a). A completely planar conformation (D_{2h} symmetry) is a higher order saddle point in all electronic states. Additionally, we have also been able to optimize several other saddle points that all lie within 1 kcal mol^{-1} that show that region around the transition state is flat with significant structural flexibility (Supplementary Fig. 51). The difference from the ring inversion TS of COT itself is probably caused by the bulky thiophene groups. After investigating and rejecting the pseudorotation mechanism we are confident that the mechanism we present is the correct one. Additionally, the transition state conformation is still nearly planar and, according to our analysis using BLA (Supplementary Fig. 40), NICS (Fig. 5c), ACID (Supplementary Figs. 34 and 36), as mentioned in the text, those transition state structures, though not perfectly planar, can still maintain its cyclic conjugation.

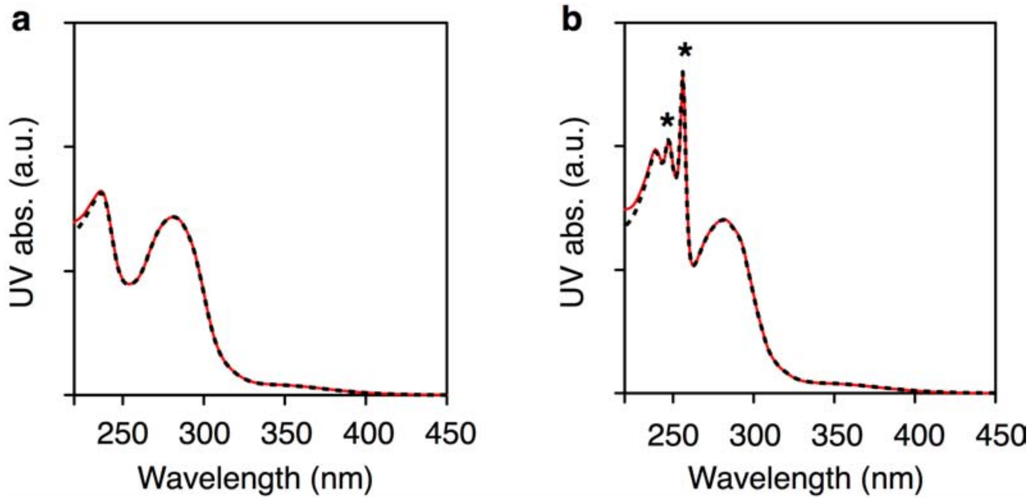

Supplementary Figure 8 | Electronic absorption spectra of ^{Th4}COT$_{Saddle}$ before and after the photochemical experiments. **a,** Electronic absorption spectra of **^{Th4}COT$_{Saddle}$** (1.8 × 10$^{–5}$ mol L$^{–1}$ in methylcyclohexane) measured at 25 °C before (black dashed line) and after (red solid line) photoirradiation (*λ* = 365 ± 5 nm). **b,** Electronic absorption spectra of **^{Th4}COT$_{Saddle}$** (2.6 × 10$^{–5}$ M in methylcyclohexane) measured at 25 °C before (black dashed line) and after (red solid line) photoirradiation (*λ* = 420 ± 5 nm) in the presence of fluorenone (8.8 × 10$^{–6}$ M). Asterisked sharp peaks at approximately 250 nm correspond to the absorption of fluorenone.

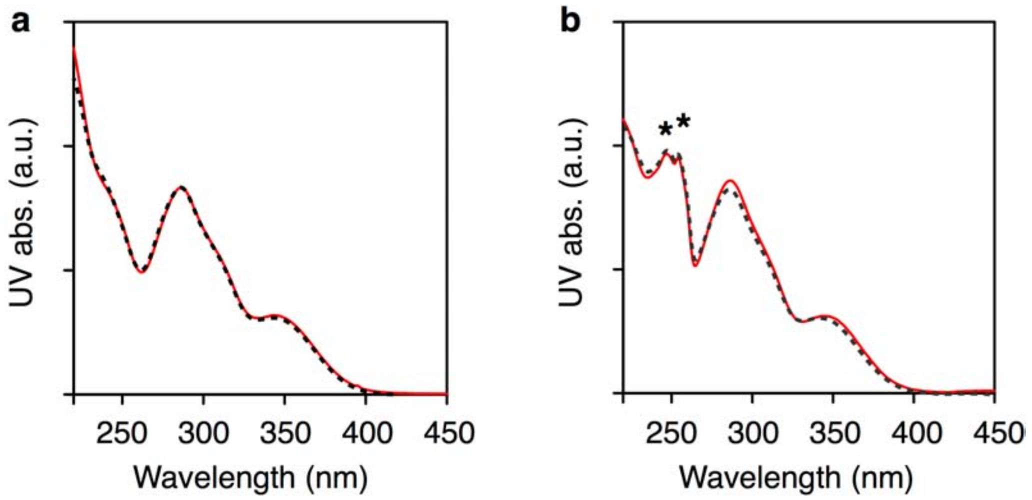

Supplementary Figure 9 | Electronic absorption spectra of ^{Th6}CDH$_{Screw}$ before and after the photochemical experiments. **a,** Electronic absorption spectra of **^{Th6}CDH$_{Screw}$** (2.4 × 10^{-5} mol L^{-1} in methylcyclohexane) measured at 25 °C before (black dashed line) and after (red solid line) photoirradiation (λ = 365 ± 5 nm). **b,** Electronic absorption spectra of **^{Th6}CDH$_{Screw}$** (1.7 × 10^{-5} M in methylcyclohexane) measured at 25 °C before (black dashed line) and after (red solid line) photoirradiation (λ = 420 ± 5 nm) in the presence of fluorenone (3.9 × 10^{-6} M). Asterisked peaks at approximately 250 nm band correspond to the absorption of fluorenone.

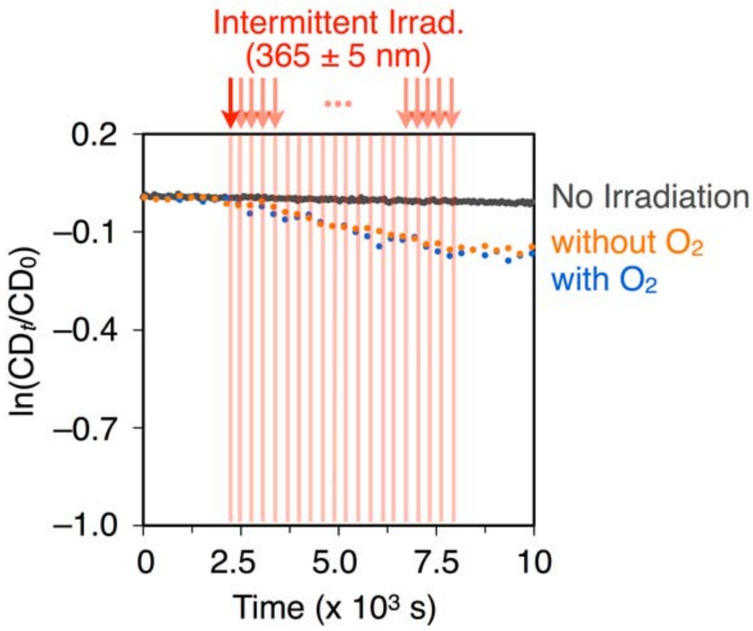

Supplementary Figure 10 | CD intensity decay profiles of ^{Th4}COT$_{Saddle}$. CD intensity decay profiles at 260 nm of **^{Th4}COT$_{Saddle}$** (1.8×10^{-5} mol L^{-1} in methylcyclohexane) measured at 20 °C. The decay profiles without photoirradiation, with photoirradiation (λ = 365 ± 5 nm) after bubbling the solution with argon for 60 min and with photoirradiation (λ = 365 ± 5 nm) after bubbling the solution with oxygen for 60 min are plotted as grey, orange and indigo dots, respectively. Photoirradiation (250 s, red vertical lines) and CD spectroscopy (50 s, white area between red vertical lines) were conducted alternately for 6000 s.

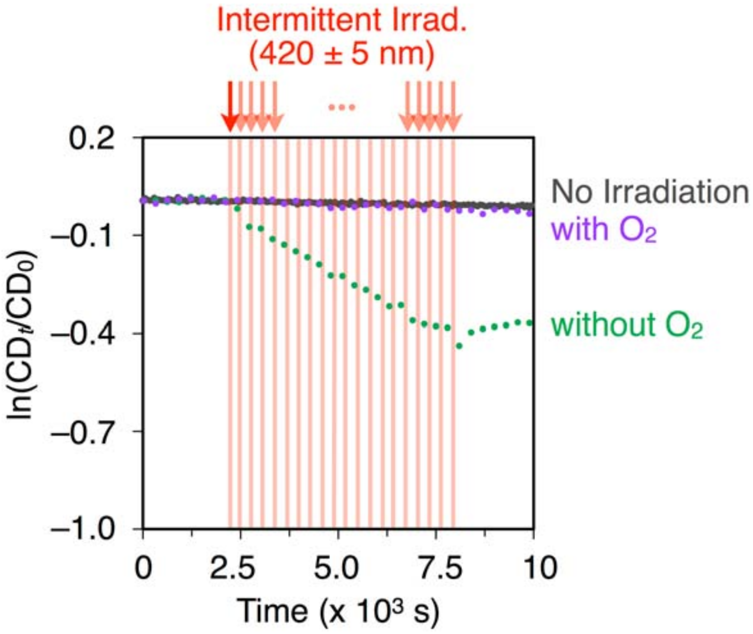

Supplementary Figure 11 | CD intensity decay profiles of ^{Th4}COT$_{Saddle}$. CD intensity decay profiles at 260 nm of **^{Th4}COT$_{Saddle}$** (2.6×10^{-5} mol L^{-1} in methylcyclohexane) in the presence of fluorenone (0.34 equiv.) measured at 20 °C. The decay profiles without photoirradiation and with photoirradiation (λ = 420 ± 5 nm) after bubbling the solution with argon for 60 min are plotted as grey and green dots, respectively. The decay profile of **^{Th4}COT$_{Saddle}$** (3.1×10^{-5} mol L^{-1} in methylcyclohexane) in the presence of fluorenone (0.44 equiv.) with photoirradiation (λ = 420 ± 5 nm) after bubbling the solution with oxygen for 60 min is plotted as purple dots. Photoirradiation (250 s, red vertical lines) and CD spectroscopy (50 s, white area between red vertical lines) were conducted alternately for 6000 s.

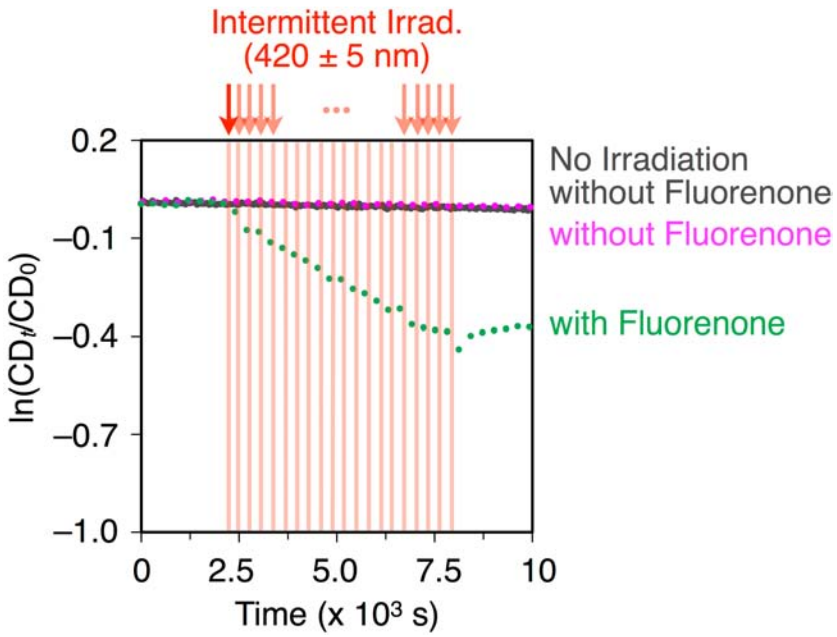

Supplementary Figure 12 | CD intensity decay profiles of ^{Th4}COT$_{Saddle}$. CD intensity decay profiles at 260 nm of **^{Th4}COT$_{Saddle}$** (2.6×10^{-5} mol L^{-1} in methylcyclohexane) measured at 20 °C after bubbling the solution with argon for 60 min. The decay profile without photoirradiation is shown using grey dots. The decay profiles with photoirradiation (λ = 420 ± 5 nm) in the presence of fluorenone (0.34 equiv.) and in the absence of fluorenone are plotted in green and magenta, respectively. Photoirradiation (250 s, red vertical lines) and CD spectroscopy (50 s, white area between red vertical lines) were conducted alternately for 6000 s.

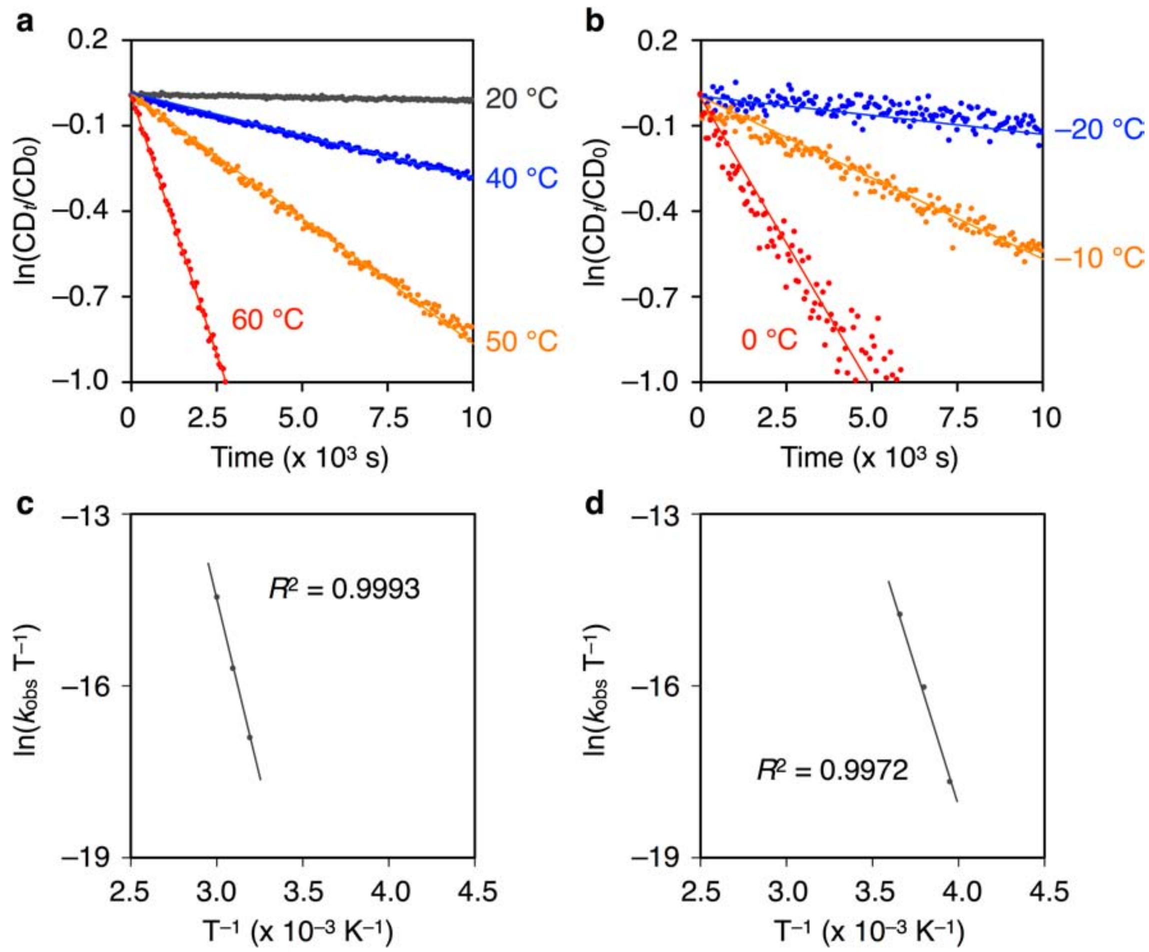

Supplementary Figure 13 | Temperature-dependent CD decay profiles of ^{Th4}COT$_{Saddle}$ and ^{Th6}CDH$_{Screw}$ in the dark. **a,** Time-dependent CD intensity decay profiles at 260 nm of **^{Th4}COT$_{Saddle}$** (3.1 × 10^{-5} mol L^{-1} in methylcyclohexane), measured at 20, 40, 50 and 60 °C, plotted in grey, blue, orange and red, respectively. The half-lives ($t_{1/2}$) of the CD intensity were 2.4 × 10^4 s (6.7 h), 7.8 × 10^3 s (2.2 h) and 2.0 × 10^3 s (0.56 h) at 40, 50 and 60 °C, respectively. **b,** Time-dependent CD decay profiles at 280 nm of **^{Th6}CDH$_{Screw}$** (2.4 × 10^{-5} mol L^{-1} in methylcyclohexane), measured at –20, –10 and 0 °C, plotted in blue, orange and red, respectively. The half-lives ($t_{1/2}$) of the CD intensity were 6.5 × 10^4 s (18 h), 1.2 × 10^4 s (3.3 h) and 3.2 × 10^3 s (0.90 h) at –20, –10 and 0 °C, respectively. **c,d,** Eyring plots, based on Supplementary Equation (3) in Supplementary Discussion, of **^{Th4}COT$_{Saddle}$** (**c**) and **^{Th6}CDH$_{Screw}$** (**d**). The activation enthalpy for the ring inversion was 25.4 kcal mol^{-1} with an estimated standard deviation of 0.8 kcal mol^{-1} (n = 3) and 20.4 kcal mol^{-1} for **^{Th4}COT$_{Saddle}$** and **^{Th6}CDH$_{Screw}$**, respectively.

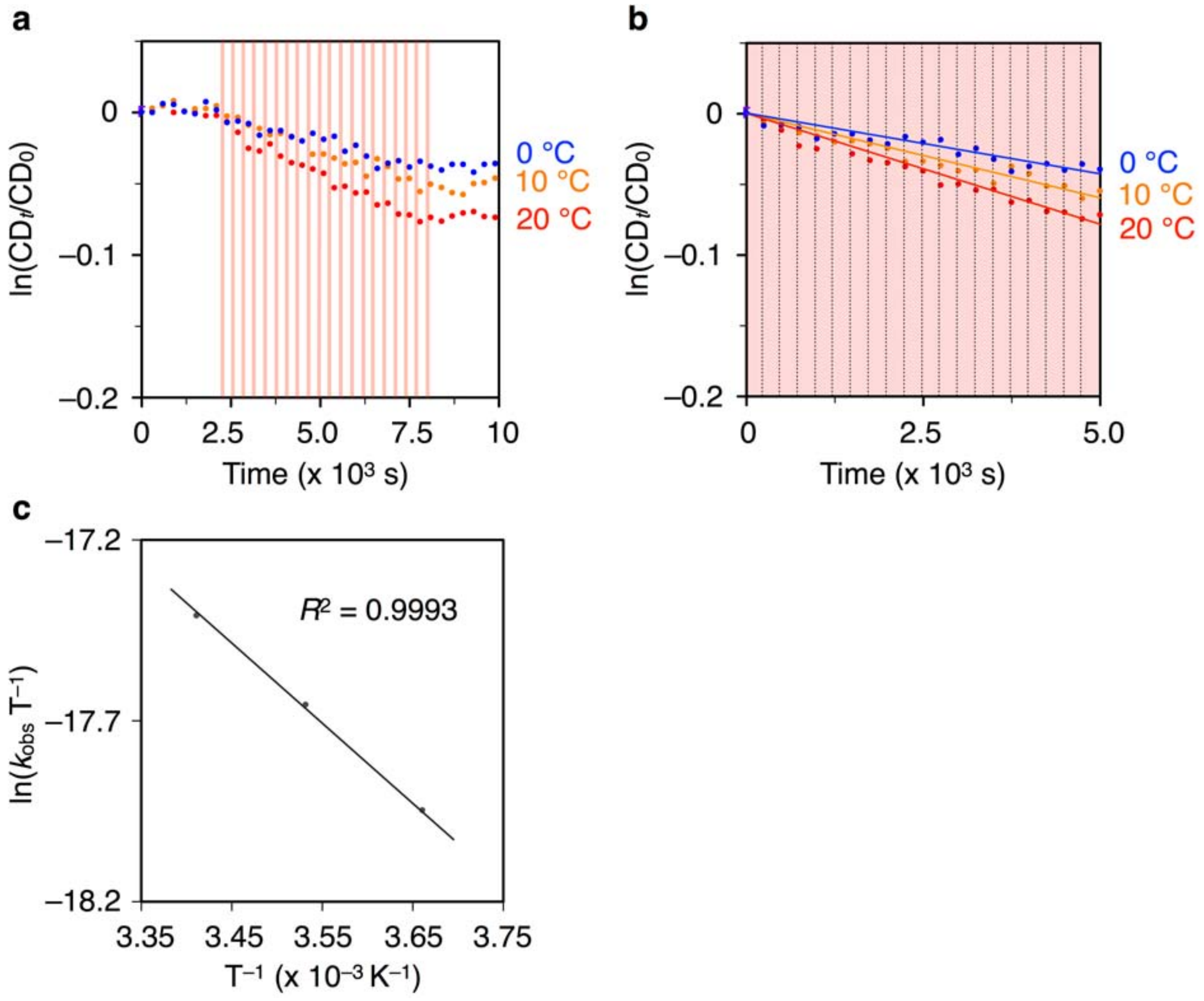

Supplementary Figure 14 | Temperature-dependent CD decay profiles of ^{Th4}COT$_{Saddle}$ under photoirradiation. **a,** Time-dependent CD intensity decay profiles at 260 nm of ^{Th4}COT$_{Saddle}$ (2.5×10^{-5} mol L^{-1} in methylcyclohexane) under photoirradiation (λ = 365 ± 5 nm) measured at 0, 10 and 20 °C, plotted in blue, orange and red, respectively. Photoirradiation (250 s, red vertical lines in **a**) and CD spectroscopy (50 s, white area between red vertical lines in **a**) were conducted alternately for 6000 s. Each dot represents the CD intensity at 260 nm. **b,** Corrected CD intensity decay profiles. The correction was performed using the methods described in Supplementary Fig. 7. **c,** Modified Eyring plot based on Supplementary Equation (5) in Supplementary Discussion. The activation enthalpy for the ring inversion was 4.3 kcal mol^{-1} with an estimated standard deviation of 0.3 kcal mol^{-1} (*n* = 3).

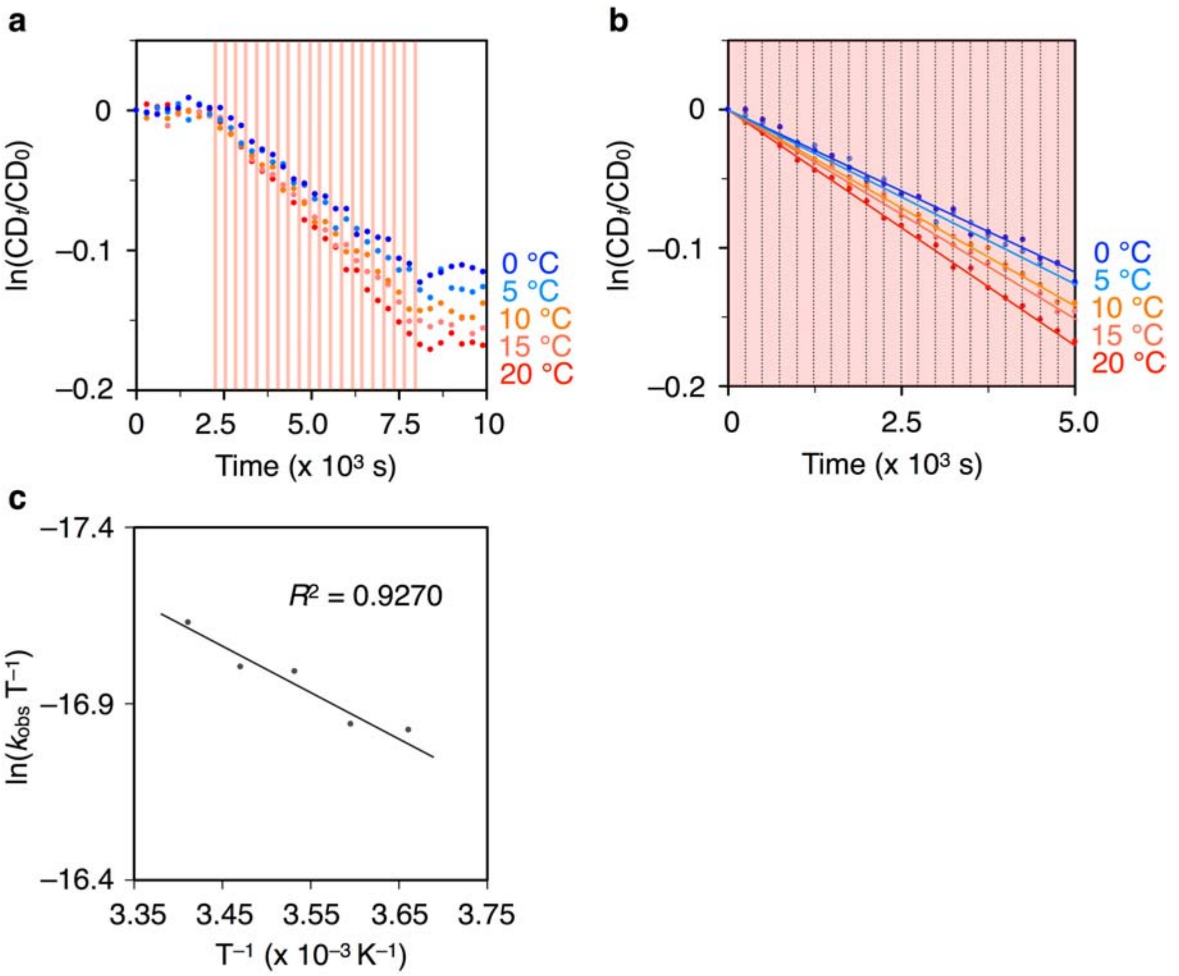

Supplementary Figure 15 | Temperature-dependent CD decay profiles of ^{Th4}COT$_{Saddle}$ under photosensitization conditions. **a,** Time-dependent CD intensity decay profiles at 260 nm of **^{Th4}COT$_{Saddle}$** (6.6 × 10^{-5} mol L^{-1} in methylcyclohexane) in the presence of fluorenone (0.12 equiv.) under photoirradiation (λ = 420 ± 5 nm) measured at 0, 5, 10, 15 and 20 °C, plotted in blue, sky-blue, orange, pink and red, respectively. Photoirradiation (250 s, red vertical lines in **a**) and CD spectroscopy (50 s, white area between red vertical lines in **a**) were conducted alternately for 6000 s. Each dot represents the CD intensity at 260 nm. **b,** Corrected CD intensity decay profiles. The correction was performed according to the methods described in Supplementary Fig. 7. **c,** Modified Eyring plot based on Supplementary Equation (5) in Supplementary Discussion. The activation enthalpy for the ring inversion was 4.0 kcal mol^{-1} with an estimated standard deviation of 1.4 kcal mol^{-1} (n = 5). The linear relationship in the modified Eyring plot supports the negligible temperature dependence of [^{3}M*] in this temperature range, as described in Supplementary Discussion.

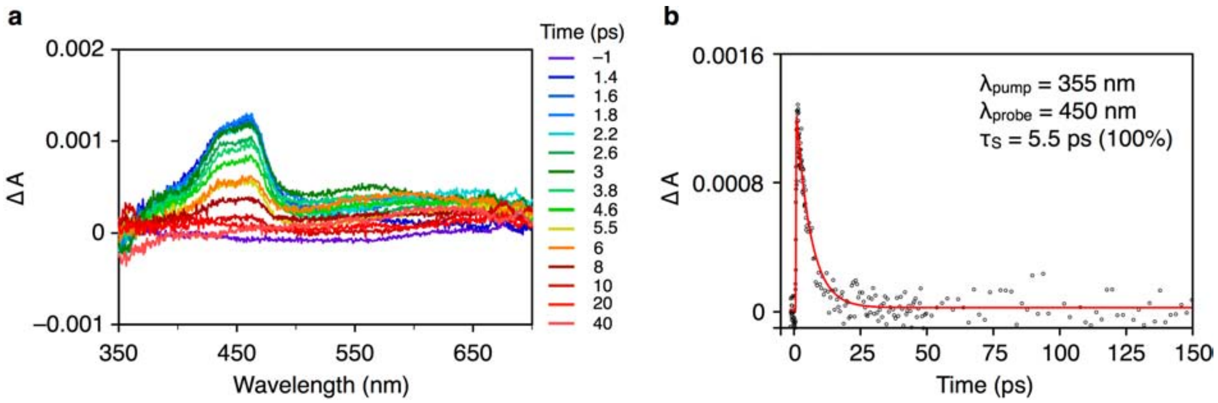

Supplementary Figure 16 | Femtosecond transient absorption spectrum (fs-TAS) and its decay profile for ^{Th4}COT$_{Saddle}$. a, fs-TAS of **^{Th4}COT$_{Saddle}$** (5.0 × 10^{-4} mol L^{-1}) with photoexcitation at 355 nm. The spectrum was recorded in deaerated methylcyclohexane at 25 °C. **b,** Femtosecond transient absorption decay profile at 450 nm, which showed a single exponential decay with a time constant of 5.5 ps.

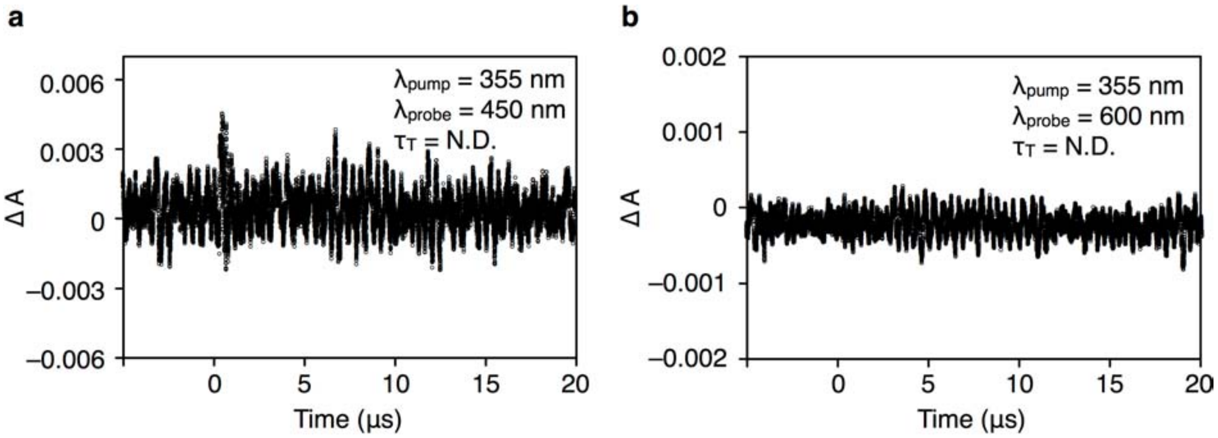

Supplementary Figure 17 | Nanosecond transient absorption (ns-TA) decay profiles for ^{Th4}COT$_{Saddle}$. a,b, Nanosecond transient absorption decay profile of **^{Th4}COT$_{Saddle}$** (5.0 × 10^{-4} mol L^{-1}) at 450 nm (**a**) and 600 nm (**b**) with photoexcitation at 355 nm. No signal was observed in the nanosecond transient measurement.

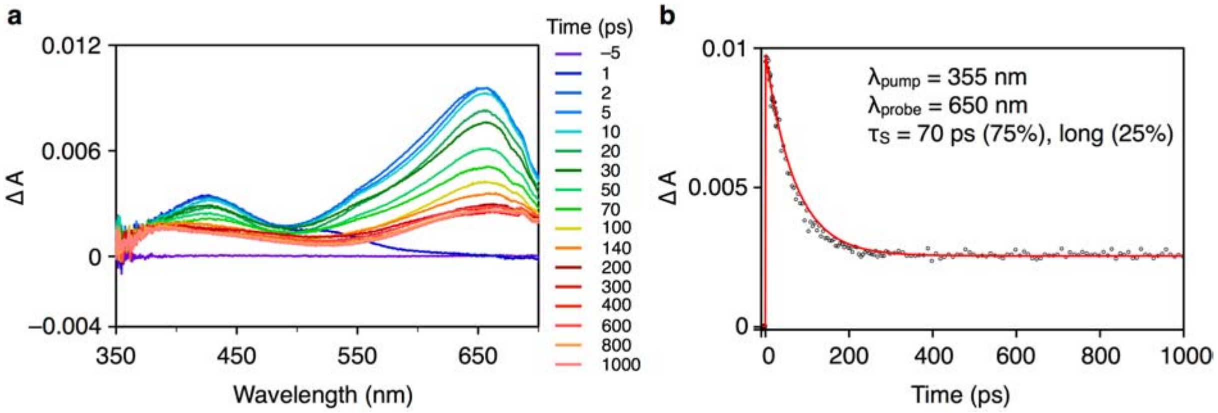

Supplementary Figure 18 | fs-TAS and its decay profile for ^{Th6}CDH$_{Screw}$. a, fs-TAS of **^{Th6}CDH$_{Screw}$** (5.0 × 10^{-4} mol L^{-1}) with photoexcitation at 355 nm. The spectrum was recorded in deaerated methylcyclohexane at 25 °C. **b,** Femtosecond transient absorption decay profile at 650 nm, which showed double exponential decay with time constants of 70 ps (75%) and that of a much longer component (25%). This result indicates the generation of both the S_1 state and T_1 states of **^{Th6}CDH$_{Screw}$**.

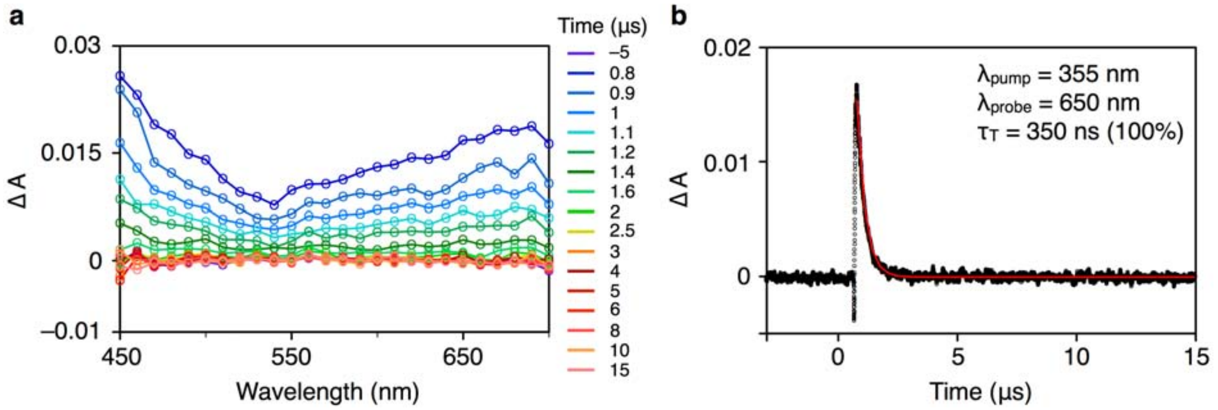

Supplementary Figure 19 | ns-TAS and its decay profile of ^{Th6}CDH$_{Screw}$. a, ns-TAS of **^{Th6}CDH$_{Screw}$** (5.0 × 10^{-4} mol L^{-1}) with photoexcitation at 355 nm. The spectrum was recorded in deaerated methylcyclohexane at 25 °C. **b,** Nanosecond transient absorption decay profile at 650 nm, which showed a single exponential decay with a time constant of 350 ns.

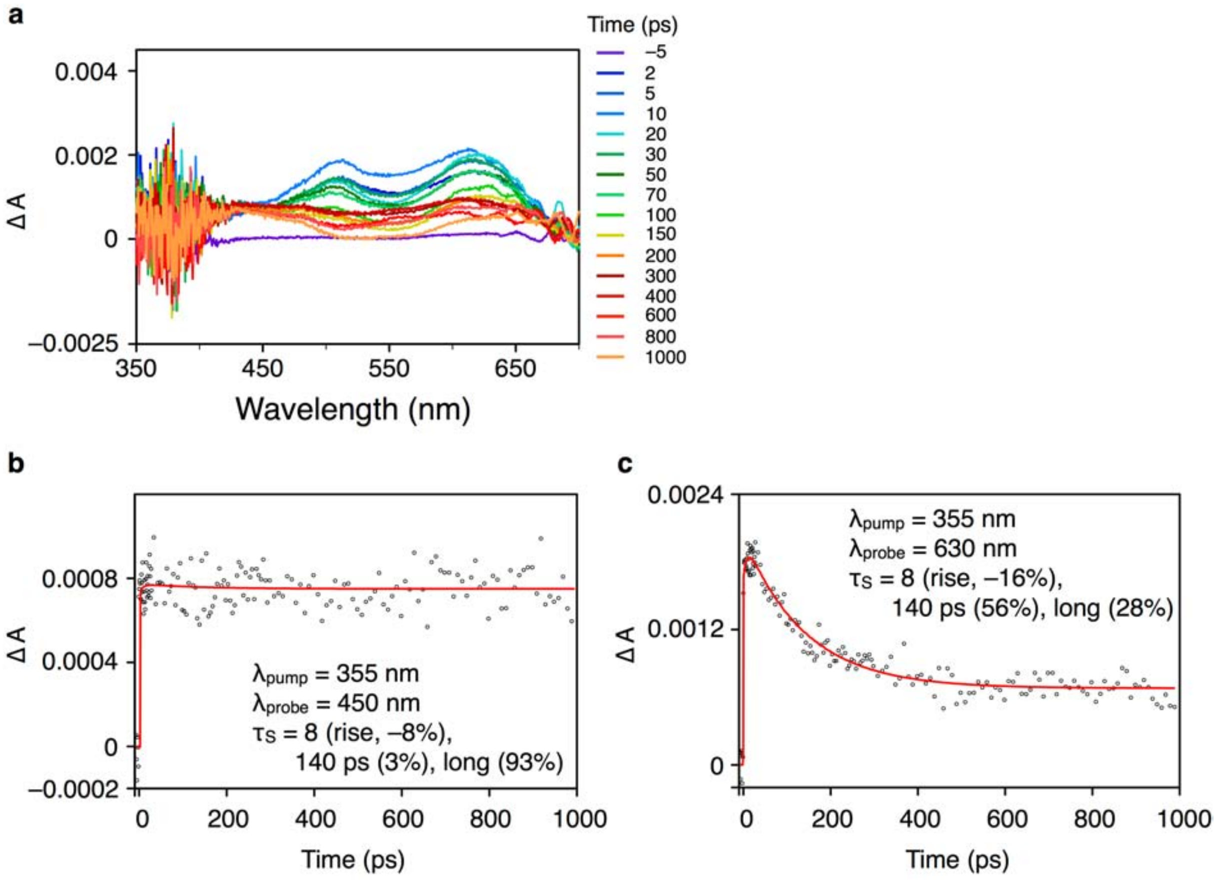

Supplementary Figure 20 | fs-TAS and its decay profile for fluorenone. a, fs-TAS of fluorenone (1.0×10^{-3} mol L^{-1}) with photoexcitation at 355 nm. The spectrum was recorded in deaerated methylcyclohexane at 25 °C. **b,** Femtosecond transient absorption decay profile at 450 nm, which showed an exponential rise and decay with time constants of 8 ps (–8%), 140 ps (3%) and that of a much longer component (93%). **c,** Femtosecond transient absorption decay profile at 630 nm, which showed an exponential rise and decay with time constants of 8 ps (–16%), 140 ps (56%) and that of a much longer component (28%).

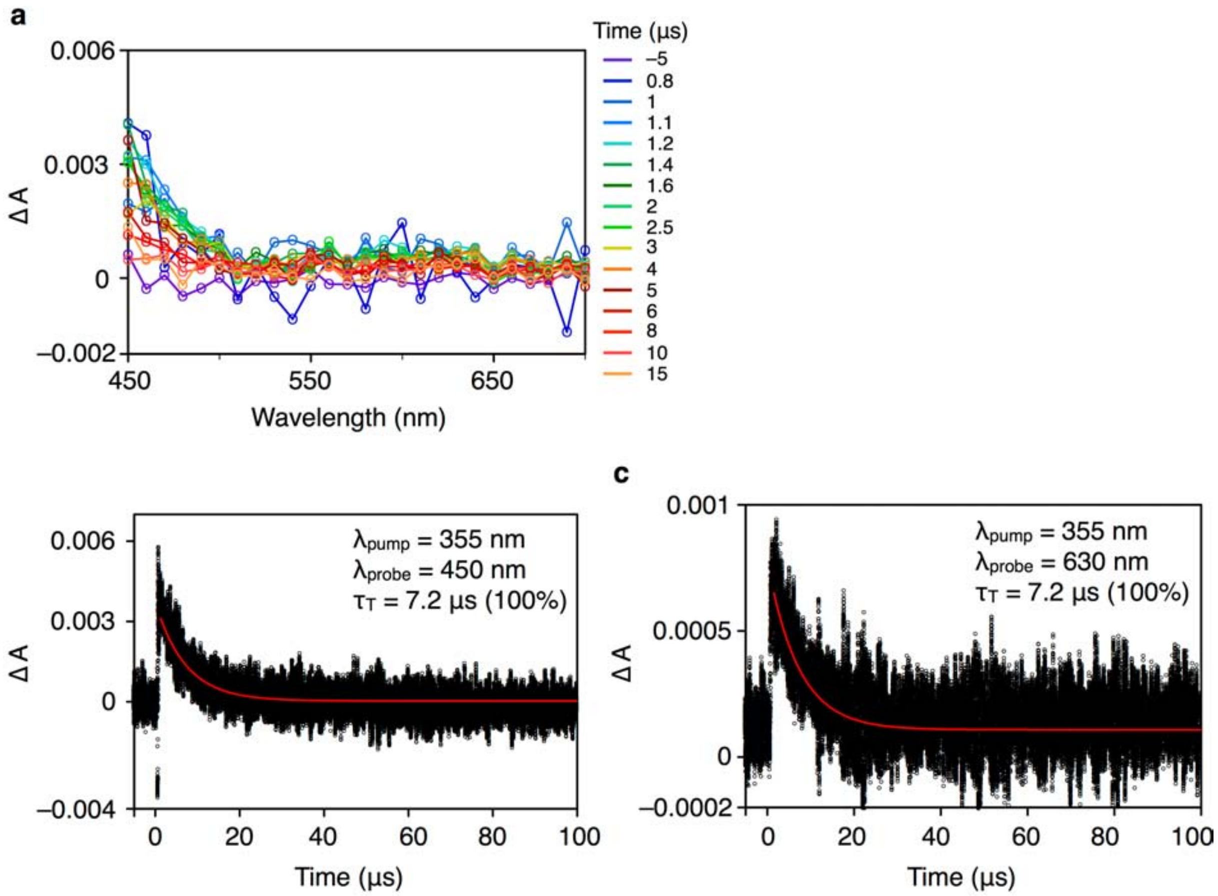

Supplementary Figure 21 | ns-TAS and its decay profile for fluorenone. **a,** ns-TAS of fluorenone (1.0×10^{-3} mol L^{-1}) with photoexcitation at 355 nm. The spectrum was recorded in deaerated methylcyclohexane at 25 °C. **b,** Nanosecond transient absorption decay profile at 450 nm, which showed a single exponential decay with a time constant of 7.2 μs. **c,** Nanosecond transient absorption decay profile at 630 nm, which showed a single exponential decay with a time constant of 7.2 μs.

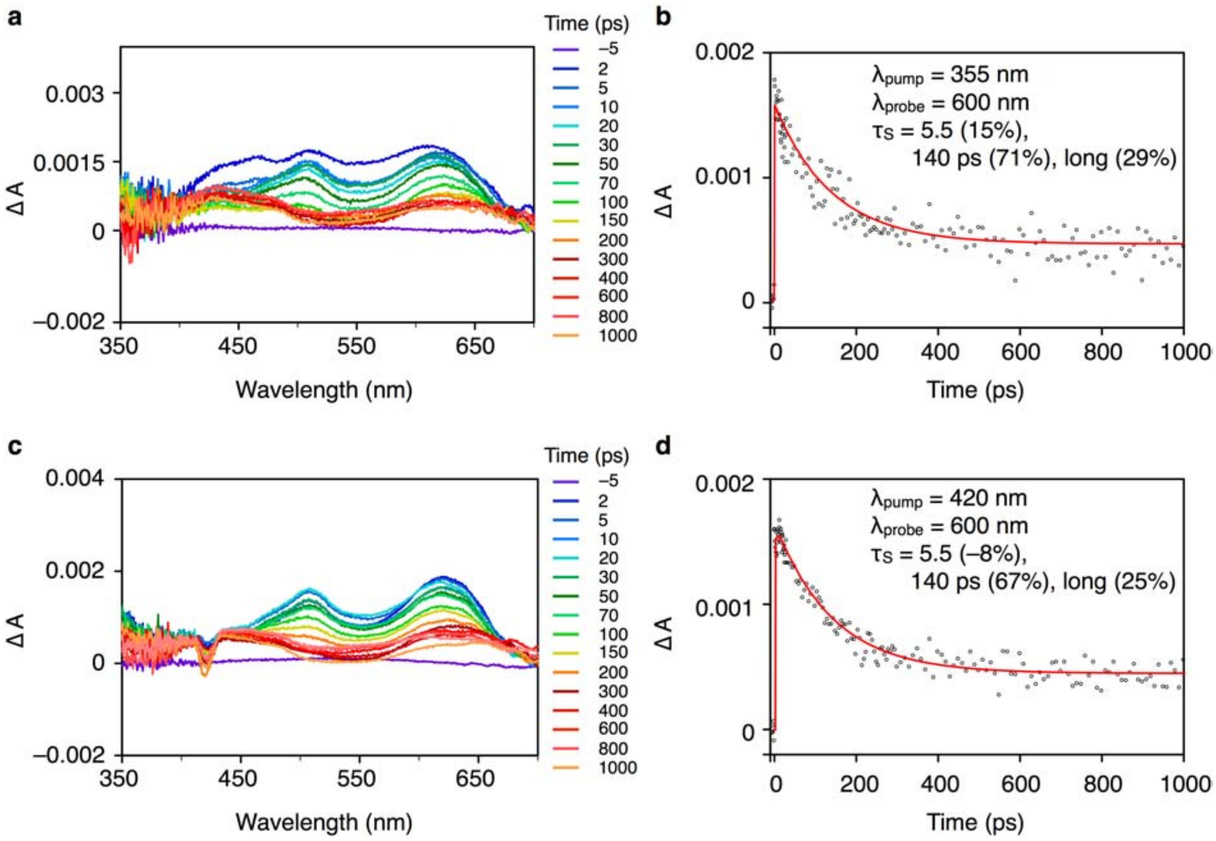

Supplementary Figure 22 | fs-TAS and its decay profile of $^{Th4}COT_{Saddle}$ in the presence of fluorenone. a, fs-TAS of **$^{Th4}COT_{Saddle}$** (2.5×10^{-4} mol L^{-1}) in the presence of fluorenone (2.5×10^{-3} mol L^{-1}) with photoexcitation at 355 nm. The spectrum was recorded in deaerated methylcyclohexane at 25 °C. **b,** Femtosecond transient absorption decay profile of TAS in **a** at 600 nm, which showed triple exponential decay with time constants of 5.5 ps (15%), 140 ps (71%) and that of a much longer component (29%). **c,** fs-TAS of **$^{Th4}COT_{Saddle}$** (1.0×10^{-4} mol L^{-1}) in the presence of fluorenone (6.0×10^{-4} mol L^{-1}) with photoexcitation at 420 nm. The spectrum was recorded in deaerated methylcyclohexane at 25 °C. **d,** Femtosecond transient absorption decay profile of TAS in **c** at 600 nm, which showed triple exponential decay with time constants of 5.5 ps (–8%), 140 ps (67%) and that of a much longer component (25%).

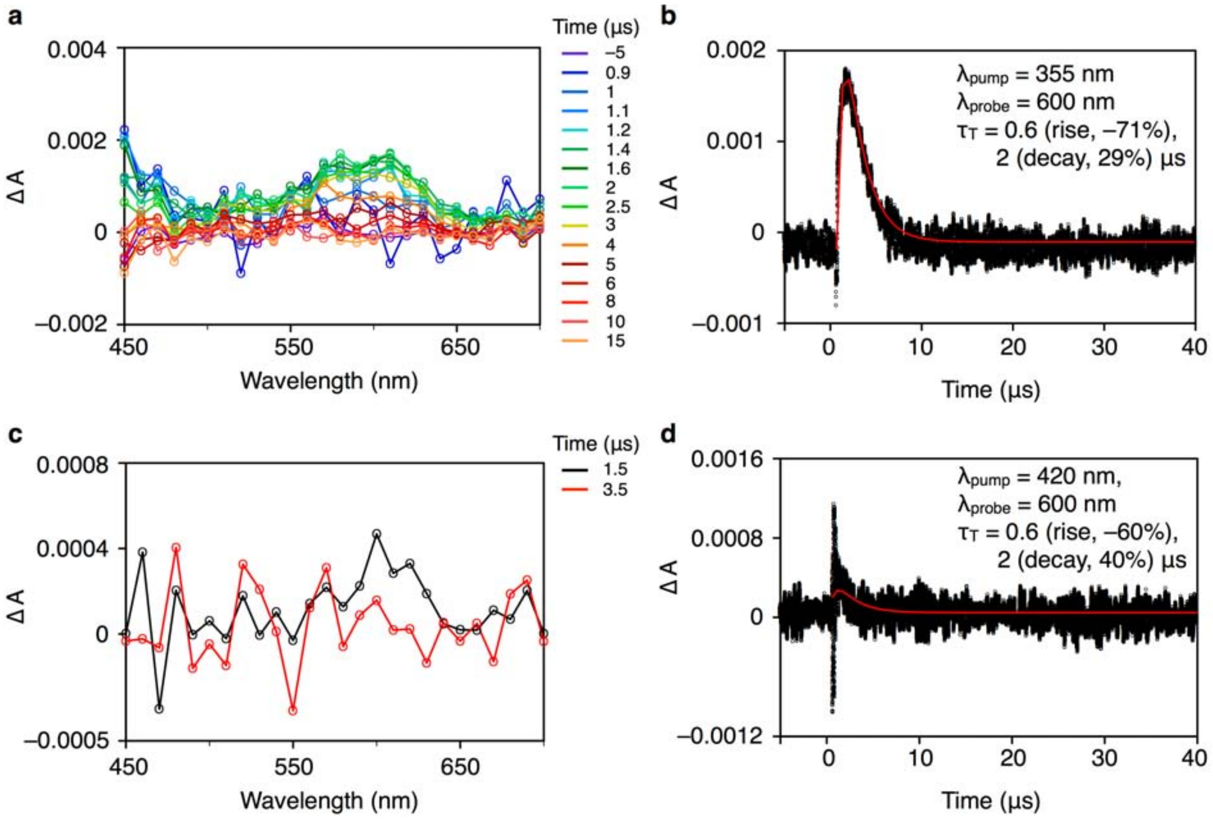

Supplementary Figure 23 | ns-TAS and its decay profile of ^{Th4}COT$_{Saddle}$ in the presence of fluorenone. **a,** ns-TAS of ^{Th4}**COT**$_{Saddle}$ (2.5×10^{-4} mol L^{-1}) in the presence of fluorenone (2.5×10^{-3} mol L^{-1}) with photoexcitation at 355 nm. The spectrum was recorded in deaerated methylcyclohexane at 25 °C. **b,** Nanosecond transient absorption decay profile of TAS in **a** at 600 nm, which showed exponential rise and decay with time constants of 0.6 μs (–71%) and 2 μs (29%). **c,** ns-TAS of ^{Th4}**COT**$_{Saddle}$ (1.0×10^{-4} mol L^{-1}) in the presence of fluorenone (6.0×10^{-4} mol L^{-1}) with photoexcitation at 420 nm. The spectrum was recorded in deaerated methylcyclohexane at 25 °C. **d,** Nanosecond transient absorption decay profile of TAS in **c** at 600 nm, which showed exponential rise and decay with time constants of 0.6 μs (–60%) and 2 μs (40%).

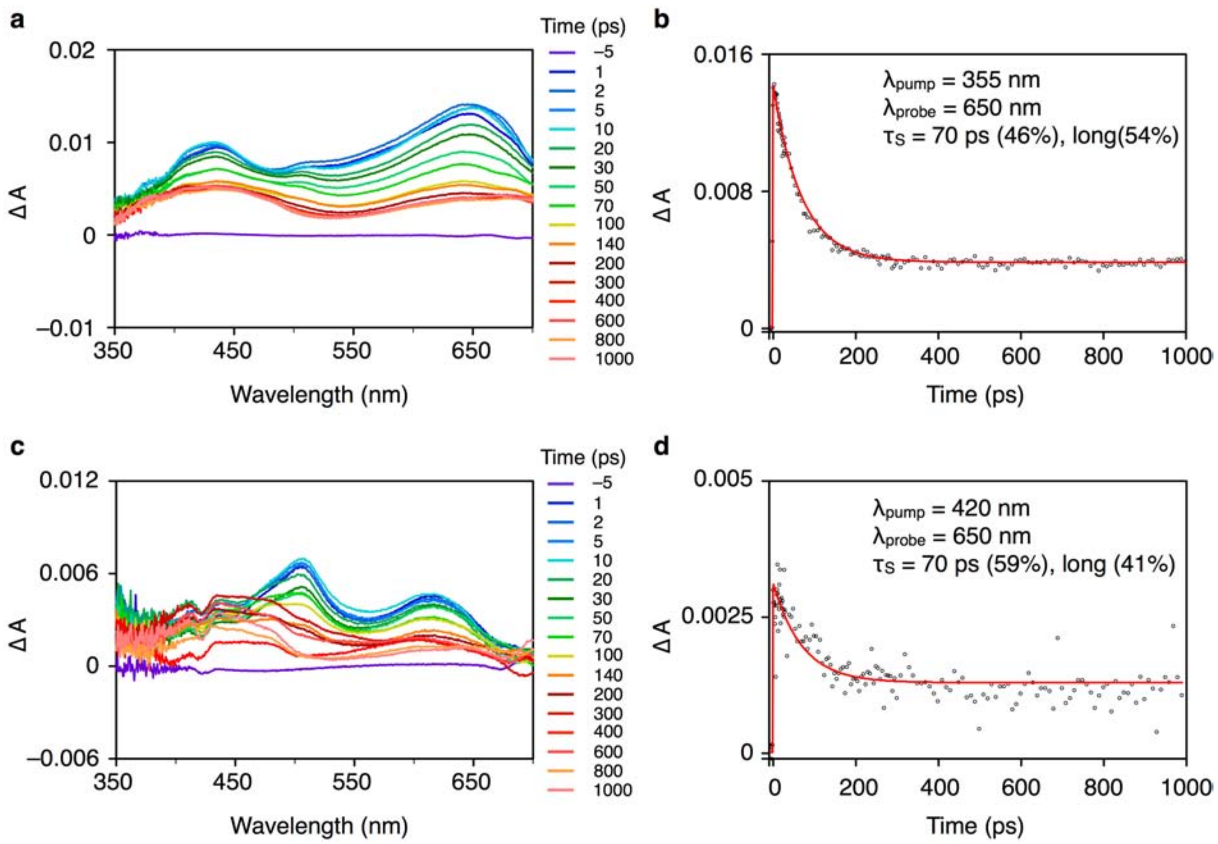

Supplementary Figure 24 | fs-TAS and its decay profile for ^{Th6}CDH$_{Screw}$ in the presence of fluorenone. **a,** fs-TAS of ^{Th6}**CDH$_{Screw}$** (2.5×10^{-4} mol L^{-1}) in the presence of fluorenone (2.5×10^{-3} mol L^{-1}) with photoexcitation at 355 nm. The spectrum was recorded in deaerated methylcyclohexane at 25 °C. **b,** Femtosecond transient absorption decay profile of TAS in **a** at 650 nm, which showed double exponential decay with time constants of 70 ps (46%) and that of a much longer component (54%). **c,** fs-TAS of ^{Th6}**CDH$_{Screw}$** (1.0×10^{-4} mol L^{-1}) in the presence of fluorenone (6.0×10^{-4} mol L^{-1}) with the photoexcitation at 420 nm. The spectrum was recorded in deaerated methylcyclohexane at 25 °C. **d,** Femtosecond transient absorption decay profile of TAS in **c** at 650 nm, which showed double exponential decay with time constants of 70 ps (59%) and that of a much longer component (41%).

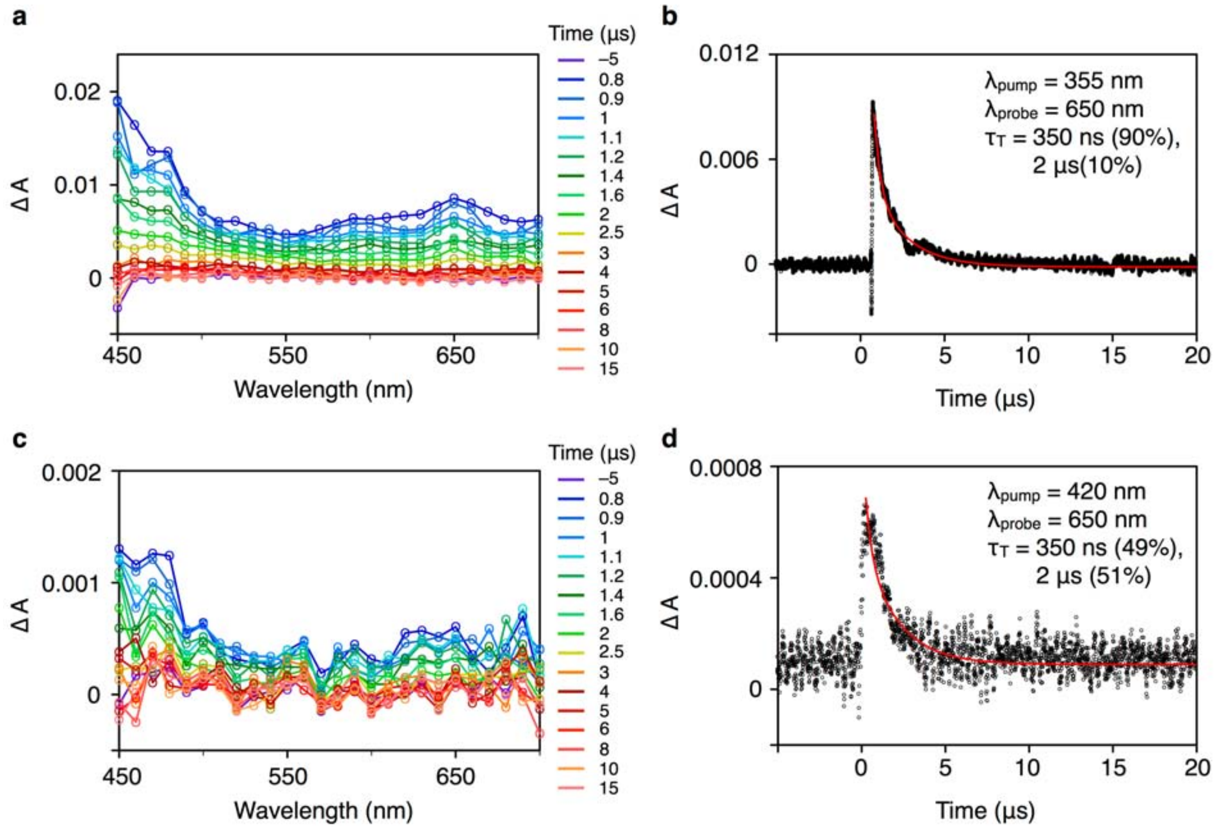

Supplementary Figure 25 | ns-TAS and its decay profile for ^{Th6}CDH$_{Screw}$ in the presence of fluorenone. **a,** ns-TAS of **^{Th6}CDH$_{Screw}$** (2.5×10^{-4} mol L^{-1}) in the presence of fluorenone (2.5×10^{-3} mol L^{-1}) with photoexcitation at 355 nm. The spectrum was recorded in deaerated methylcyclohexane at 25 °C. **b,** Nanosecond transient absorption decay profile of TAS in **a** at 650 nm, which showed double exponential decay with time constants of 350 ns (90%), 2 μs (10%). **c,** ns-TAS of **^{Th6}CDH$_{Screw}$** (1.0×10^{-4} mol L^{-1}) in the presence of fluorenone (6.0×10^{-4} mol L^{-1}) with photoexcitation at 420 nm. The spectrum was recorded in deaerated methylcyclohexane at 25 °C. **d,** Nanosecond transient absorption decay profile of TAS in **c** at 650 nm, which showed double exponential decay with time constants of 350 ns (49%), 2 μs (51%). Note that 30 equiv. of fluorenone was used in this series of experiment in order to obtain a signal with sufficient intensity.

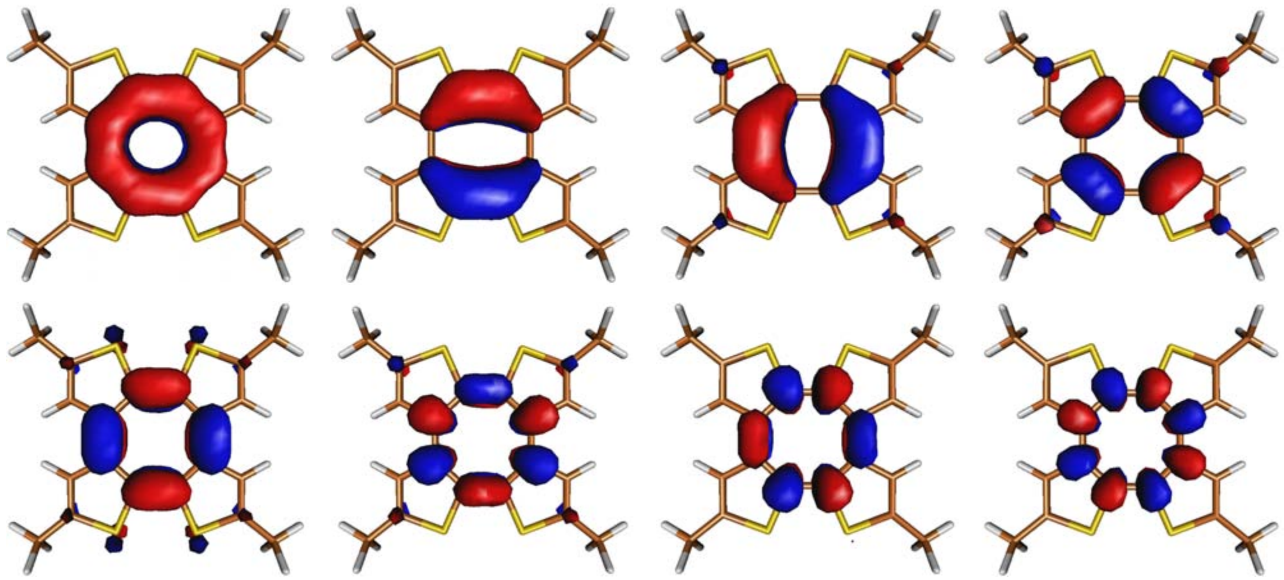

Supplementary Figure 26 | Molecular orbitals comprising the active space of ^{Th4}COT$_{Saddle}$. Example from calculation of the transition state structure in the S_1 state, plotted at an isodensity surface value of 0.04 a.u.

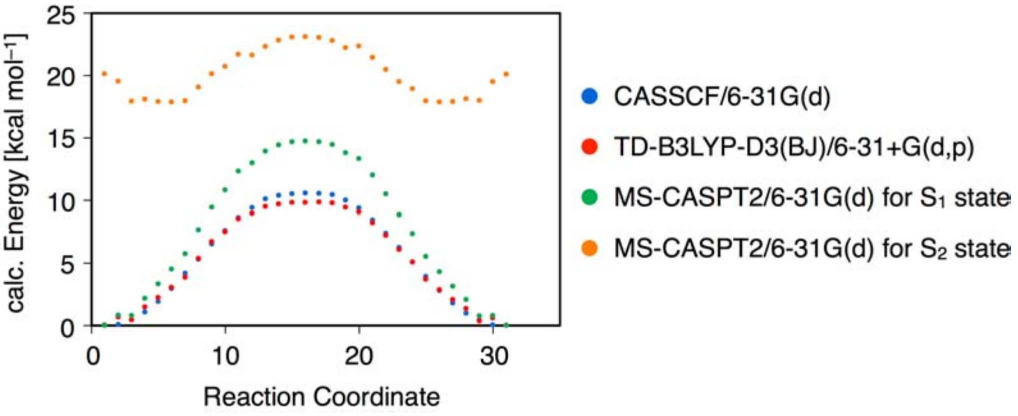

Supplementary Figure 27 | Activation energy barriers in the singlet excited sate. Activation energy barriers in the S_1 state along the CASSCF pseudo-IRC for CASSCF/6-31G(d), TD-B3LYP-D3(BJ)/6-31+G(d,p) and MS-CASPT2/6-31G(d).

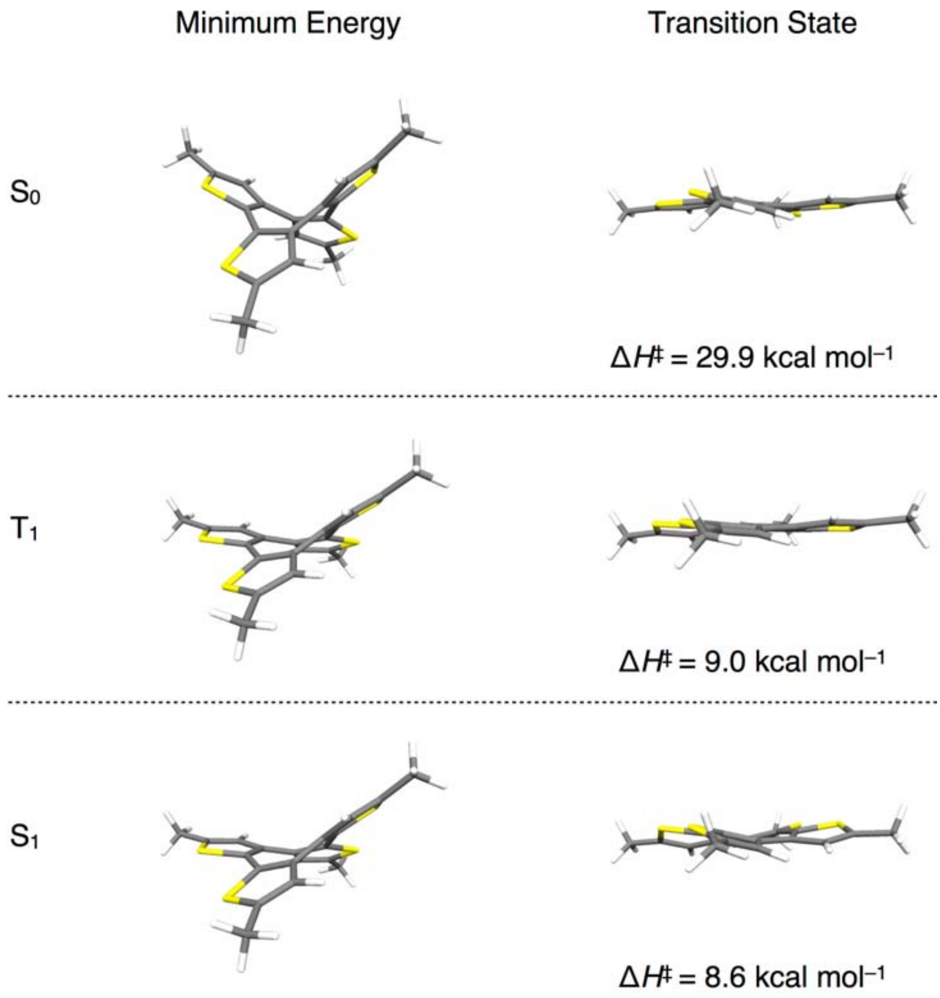

Supplementary Figure 28 | Calculated structures and energies for $^{Th4}COT_{Saddle}$. B3LYP-D3(BJ)/6-311+G(d,p)//6-31G(d) was used for the S_0 and T_1 states, whereas TD-B3LYP-D3(BJ)/6-31+G(d,p) was used for the S_1 states.

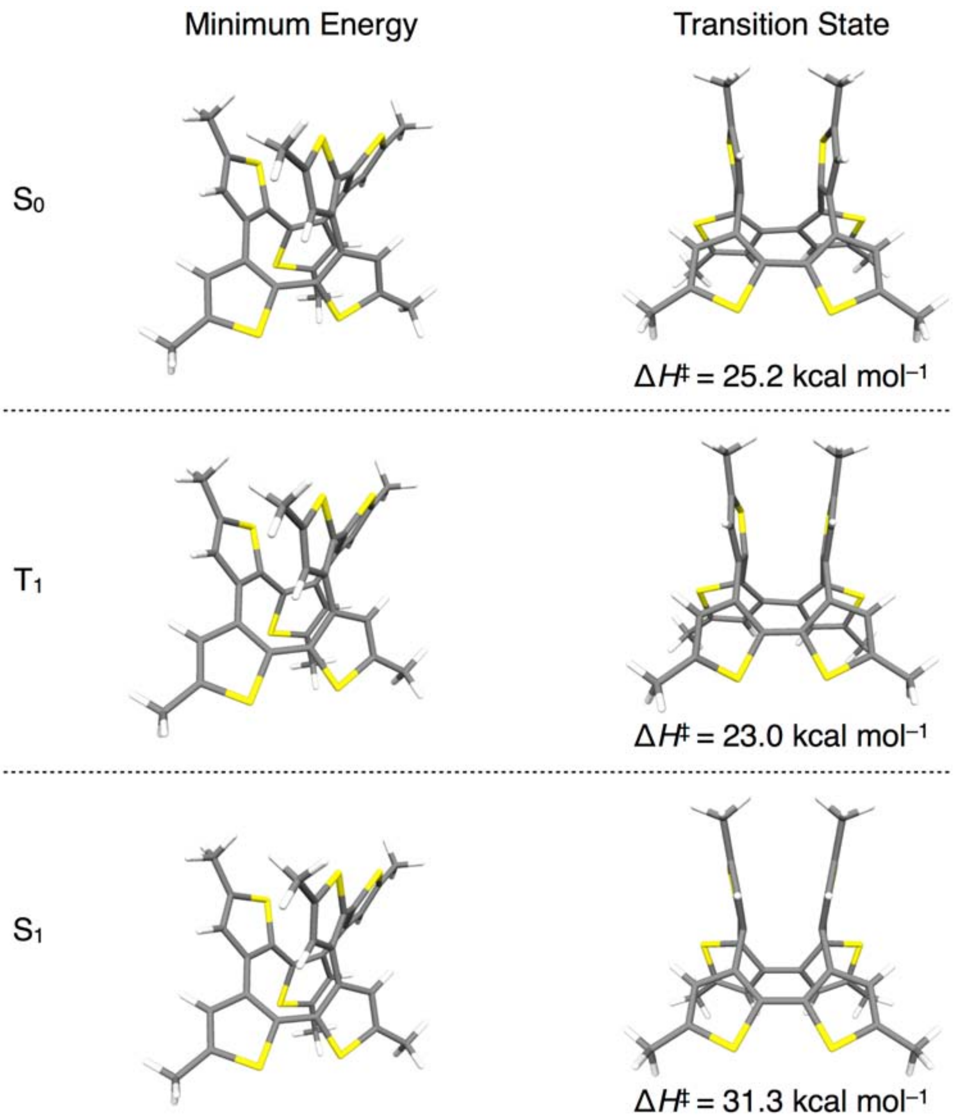

Supplementary Figure 29 | Calculated structures and energies for ^{Th6}CDH$_{Screw}$. B3LYP-D3(BJ)/6-311+G(d,p)//6-31G(d) was used for the S_0 and T_1 states, whereas TD-B3LYP-D3(BJ)/6-31+G(d,p) was used for the S_1 states.

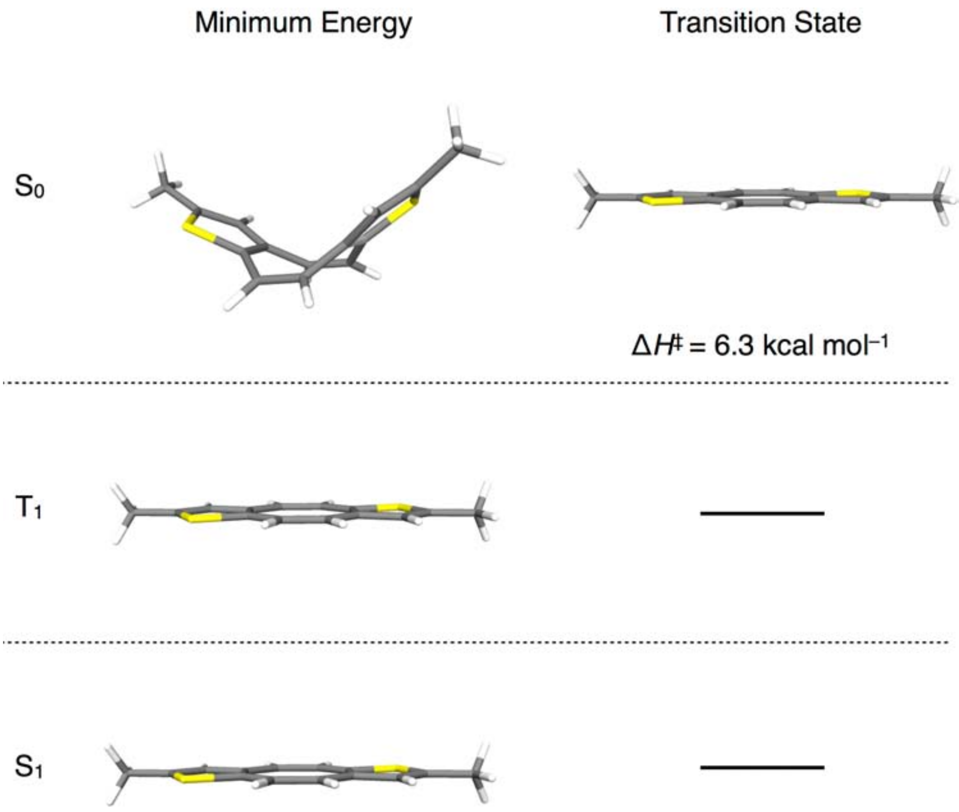

Supplementary Figure 30 | Calculated structures and energies for ^{Th2}COT$_{Saddle}$. The activation energy for the S_0 state is low compared to that of **^{Th4}COT$_{Saddle}$** due to less steric repulsion in the planar transition state. For the same reason, the T_1 and S_1 states exhibit planar minimum energy structures. Structures were optimized with B3LYP-D3(BJ)/6-31G(d), and single-point energies were calculated using the 6-311+G(d,p) basis set.

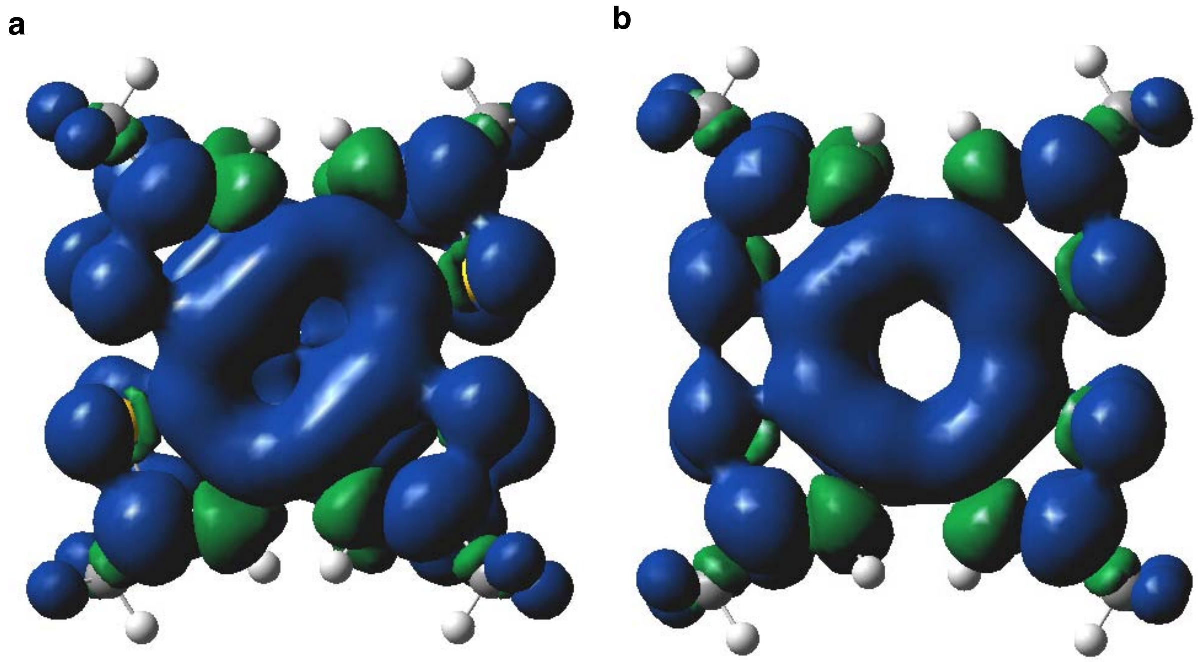

Supplementary Figure 31 | Spin densities for $^{Th4}COT_{Saddle}$ in the T_1 state. The minimum energy structure (**a**), and the transition state structure (**b**) in the T_1 state. Delocalization of the spin density (excess α spin due to the triplet state) in the central COT moiety indicates the influence of Baird aromaticity in this ring. Small delocalization of the spin also occurs to the thiophene units. The spin density maps are obtained at the B3LYP/6-31G(d) level and presented with an isodensity surface value of 0.0004.

a b

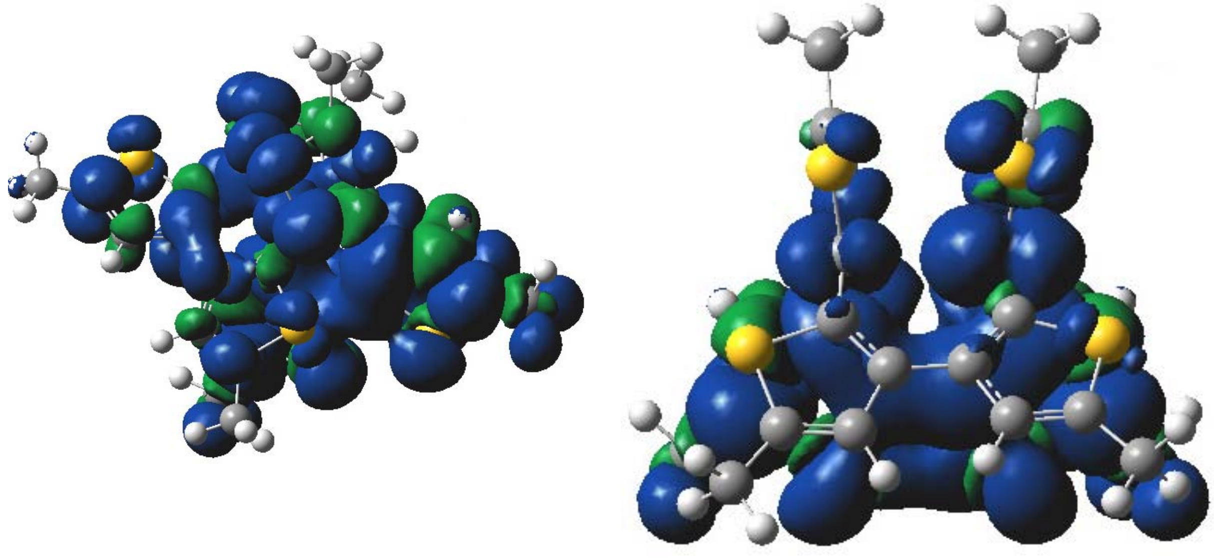

Supplementary Figure 32 | Spin densities for $^{Th6}CDH_{Screw}$ in the T_1 state. The minimum energy structure (**a**), and the transition state structure (**b**) in the T_1 state. Localization of the spin density (excess α spin due to the triplet state) to one side of the molecule is seen clearly in the transition state structure and for the minimum energy structure, although the screw conformation makes it more difficult to observe. The absence of spin delocalization in the transition state structure is strong evidence against Baird aromatic stabilization. The spin density maps are obtained at the B3LYP/6-31G(d) level and presented with an isodensity surface value of 0.0004.

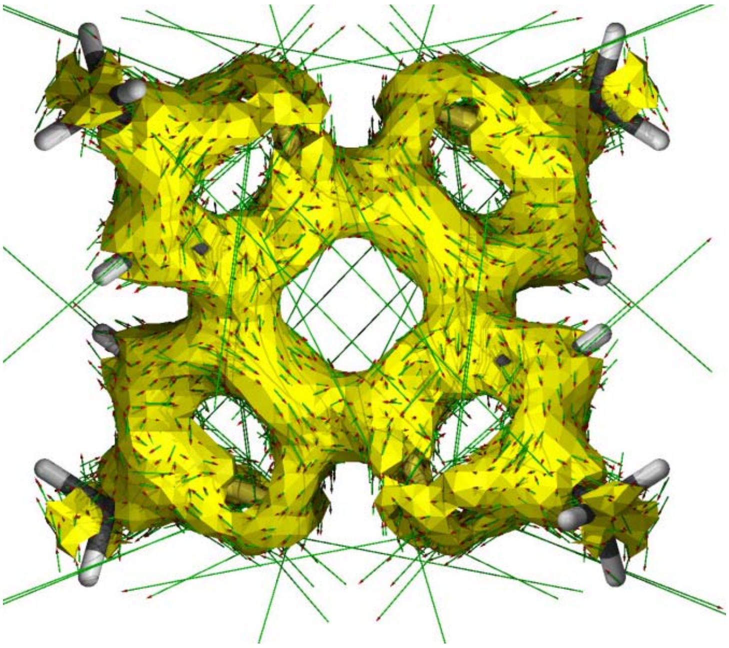

Supplementary Figure 33 | ACID plot of $^{Th4}COT_{Saddle}$, the saddle-shaped minimum energy structure in the S_0 state.

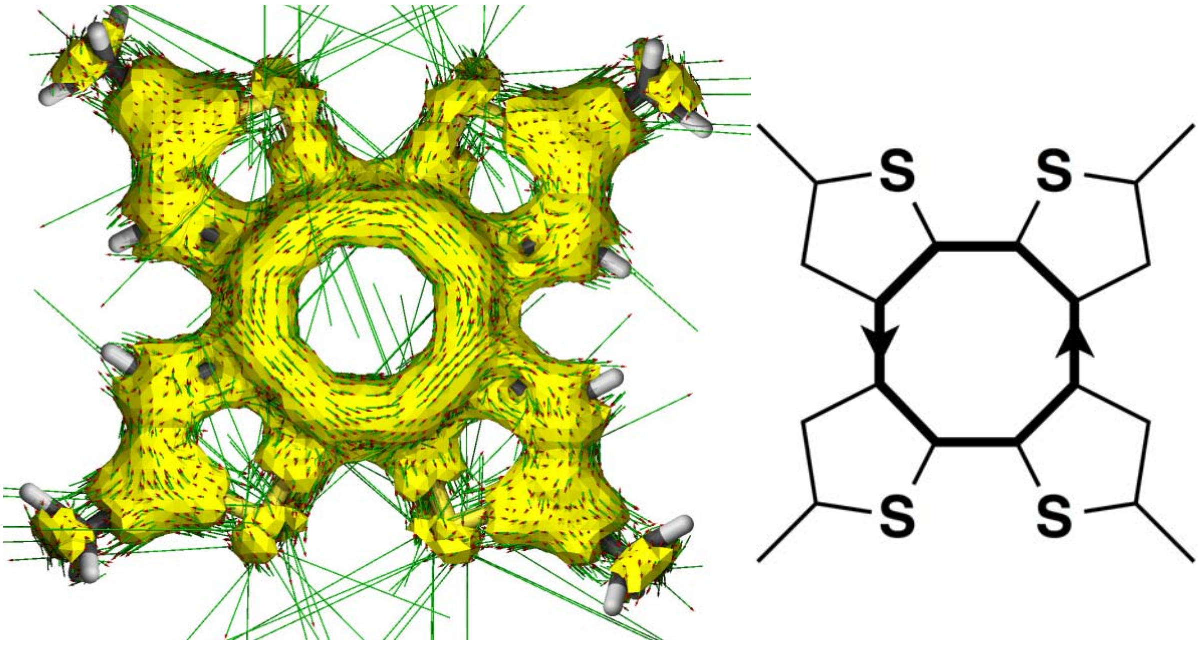

Supplementary Figure 34 | ACID plot of $^{Th4}COT_{Saddle}$, the transition state structure in the S_0 state.

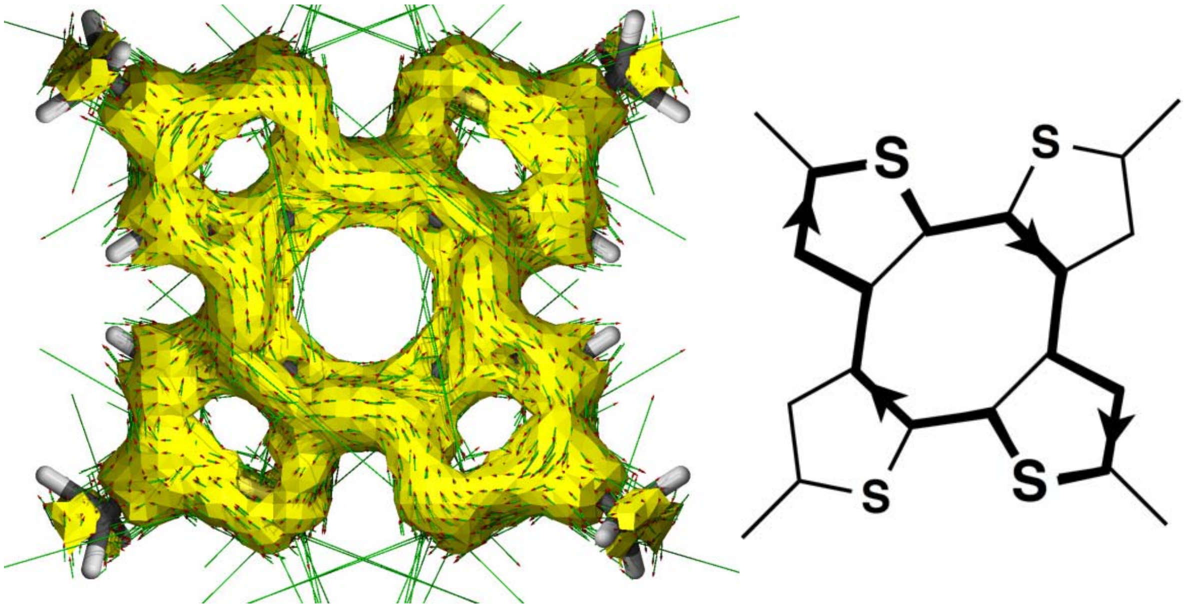

Supplementary Figure 35 | ACID plot of $^{Th4}COT_{Saddle}$, the saddle-shaped minimum energy structure in the T_1 state.

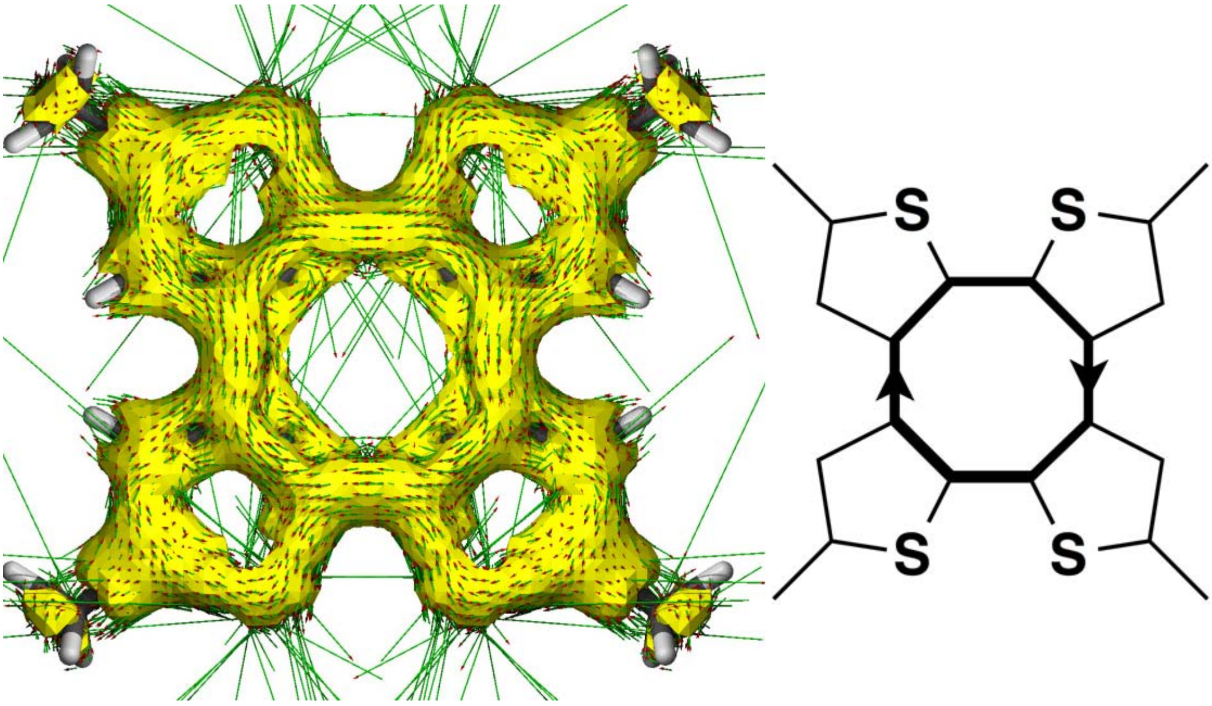

Supplementary Figure 36 | ACID plot of $^{Th4}COT_{Saddle}$, the transition state structure in the T_1 state.

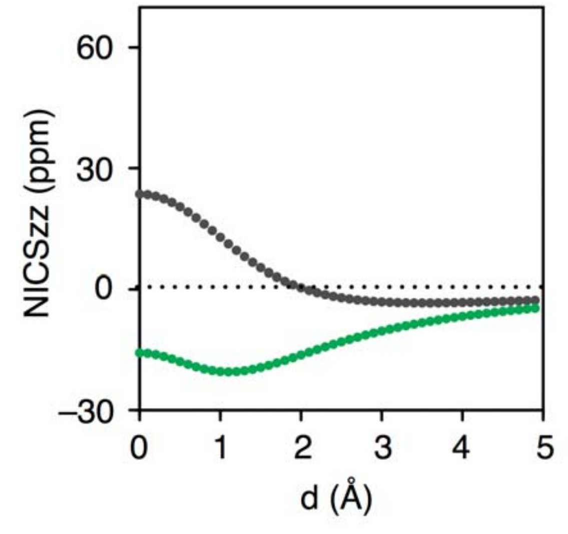

Supplementary Figure 37 | NICS$_{zz}$ scan of ^{Th4}COT$_{Saddle}$ in the S$_0$ and T$_1$ minimum energy structures. NICS$_{zz}$ scans orthogonal to the central COT ring, performed at the B3LYP/6-311+G(d,p) level, in the S$_0$ and T$_1$ states are plotted in black and green, respectively.

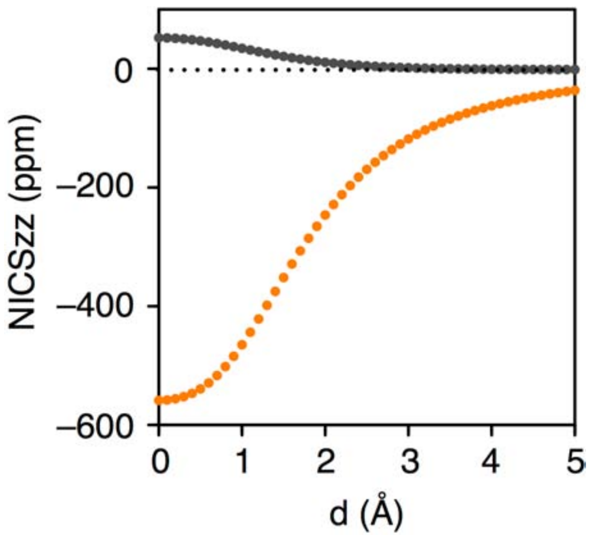

Supplementary Figure 38 | NICS$_{zz}$ scan of ^{Th4}COT$_{Saddle}$ in the S$_0$ and S$_1$ states at the S$_1$ minimum energy structure. NICS$_{zz}$ scans orthogonal to the central COT ring, performed at the CASSCF(8in8)/6-31++G(d,p) level, in the S$_0$ and S$_1$ states are plotted in black and orange, respectively. The results are qualitative, and the absolute value of NICS should not be taken as a measure of the strength of the aromaticity.

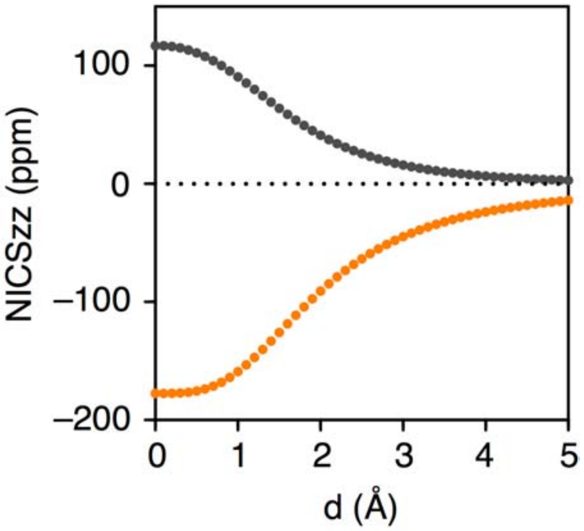

Supplementary Figure 39 | $NICS_{zz}$ scan of $^{Th4}COT_{Saddle}$ in the S_0 and S_1 states at the S_1 transition state structures. $NICS_{zz}$ scans orthogonal to the central COT ring, performed at the CASSCF(8in8)/6-31++G(d,p) level, in the S_0 and S_1 states are plotted in black and orange, respectively. The results are qualitative, and the absolute value of the NICS should not be taken as a measure of the strength of the aromaticity.

Supplementary Table 7 | Calculated $NICS_{iso}(1)$ values for π-conjugated circuits in $^{Th4}COT_{Saddle}$. The numbers separated by a solidus represent NICS values above and below the ring.

In S_0 State	COT ring	Thiophene 1	Thiophene 2
Minimum Energy	0.0/0.0	–6.8/–7.6	–6.8/–7.6
Transition State	11.9/11.9	–4.5/–4.8	–5.1/–4.1
In T_1 State	COT ring	Thiophene 1	Thiophene 2
Minimum Energy	–8.9/–8.9	–7.3/–4.4	–7.3/–4.4
Transition State	–9.7/–9.7	–4.8/–5.7	–4.9/–5.9

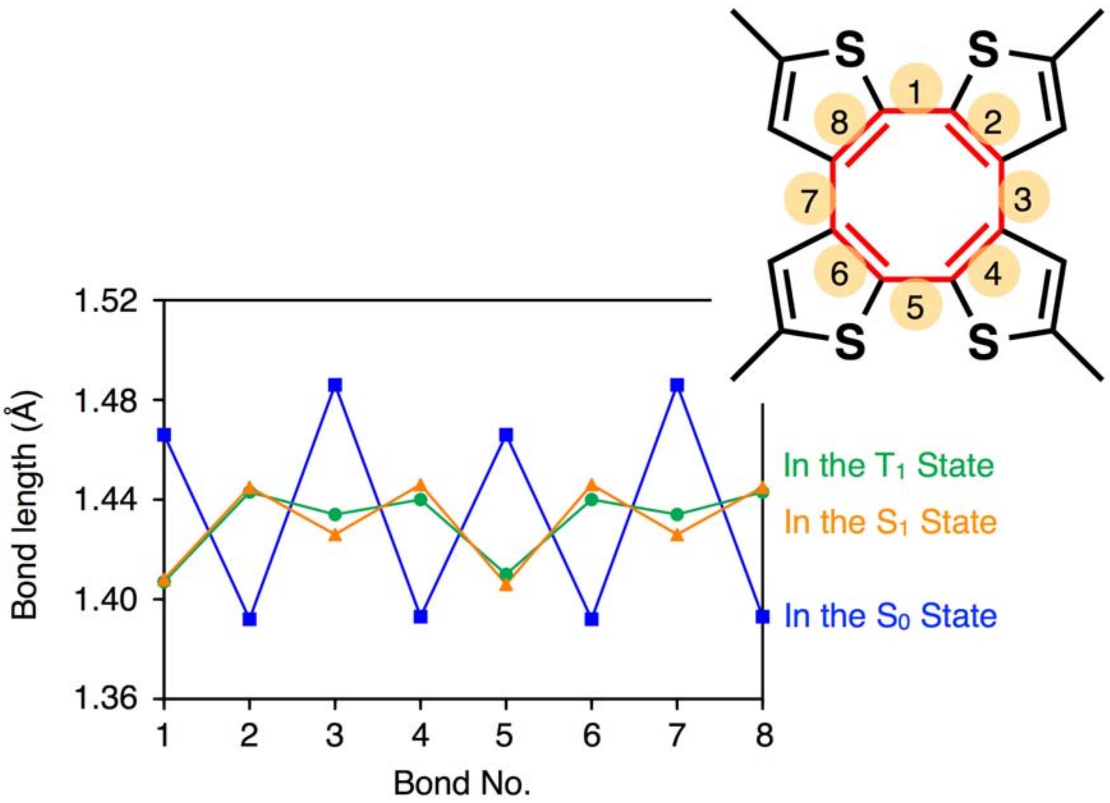

Supplementary Figure 40 | BLA of the transition state structures of $^{Th4}COT_{Saddle}$ in the S_0, T_1 and S_1 states. The lengths of C–C bonds in the COT ring, indexed using numerals in the inset figure, are plotted for the transition state structures in the S_0, T_1 and S_1 states as blue, green and orange lines, respectively. The BLA for the S_1 and T_1 states is similar. Therefore, the aromaticity of the S_1 state should be similar to that of the T_1 state.

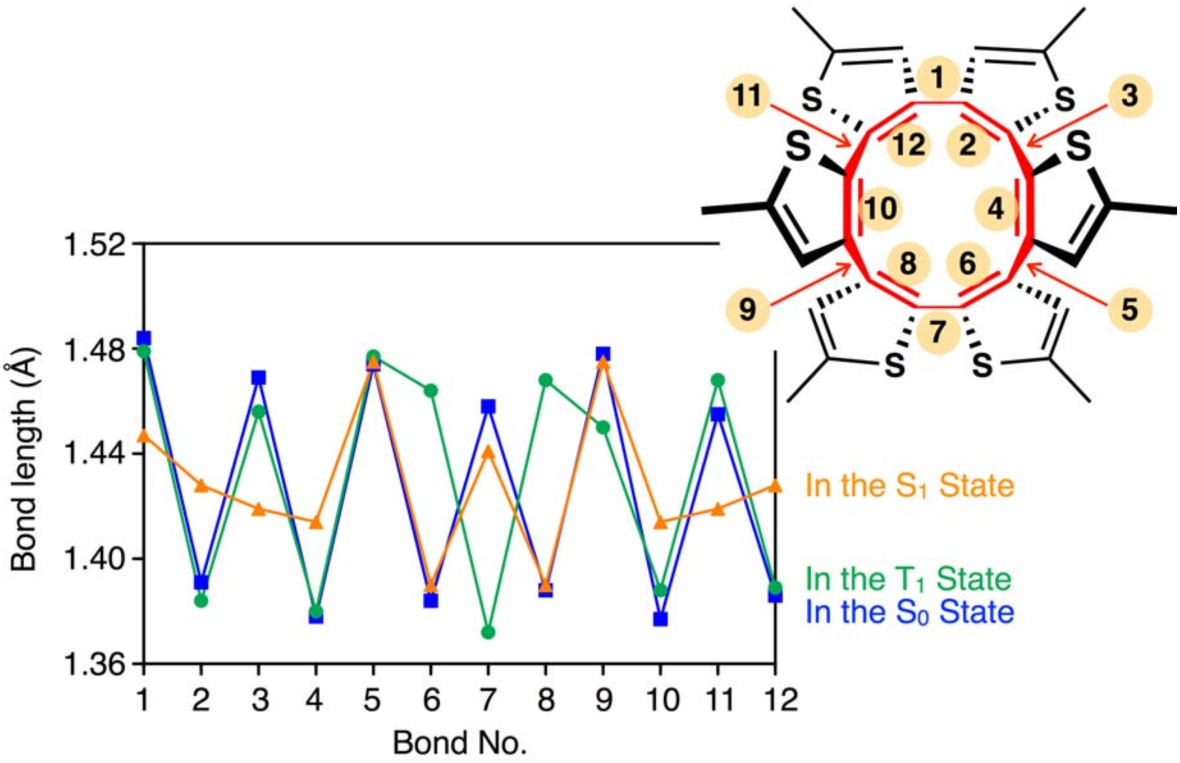

Supplementary Figure 41 | BLA of the transition state structures of $^{Th6}CDH_{Screw}$ in the S_0, T_1 and S_1 states. The lengths of the C–C bonds in the cyclododecahexaene ring ([12]annulene), indexed using numerals in the inset figure, are plotted for the transition state structures in the S_0, T_1 and S_1 states as blue, green and orange lines, respectively. All the BLA values were similar and large, indicating the absence of aromatic stabilization. Moreover, all the transition state structures in the S_0, S_1 and T_1 states exhibit multiple large dihedral angles of ca. 70° or more, indicating weak or non-existent cyclic conjugation (Supplementary Fig. 44).

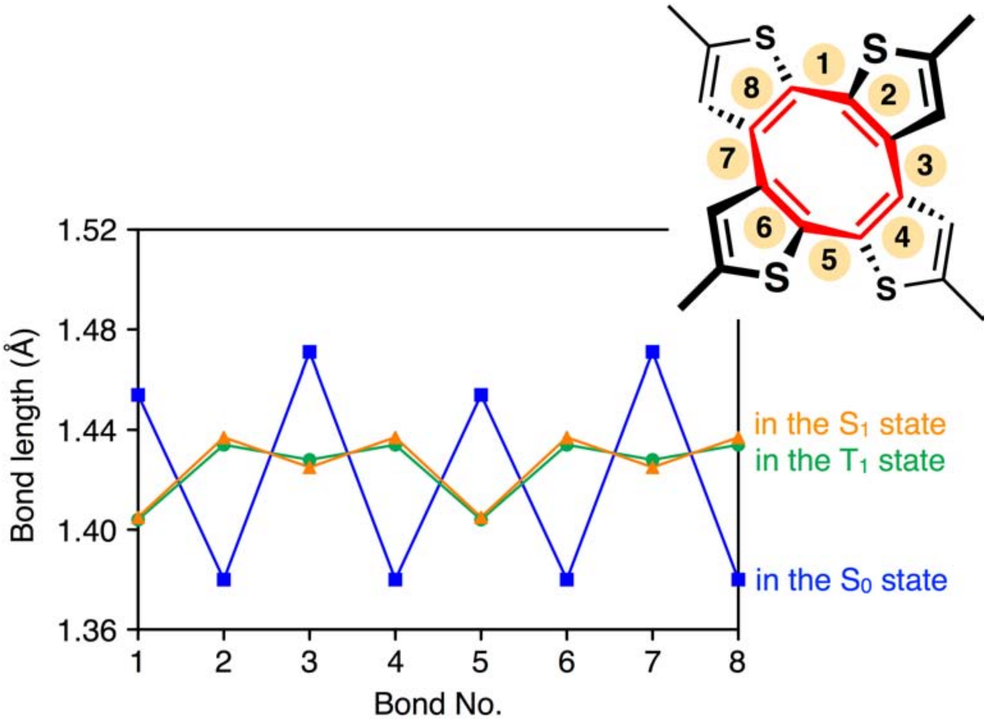

Supplementary Figure 42 | BLA of the minimum energy structures of $^{Th4}COT_{Saddle}$ in the S_0, T_1 and S_1 states. The lengths of the C–C bonds in the COT ring, indexed using numerals in the inset figure, are plotted for the minimum energy structure in the S_0, T_1 and S_1 states as blue, green and orange lines, respectively. The BLA for the S_1 and T_1 states is similar. Therefore, the aromaticity of the S_1 state should be similar to that of the T_1 state.

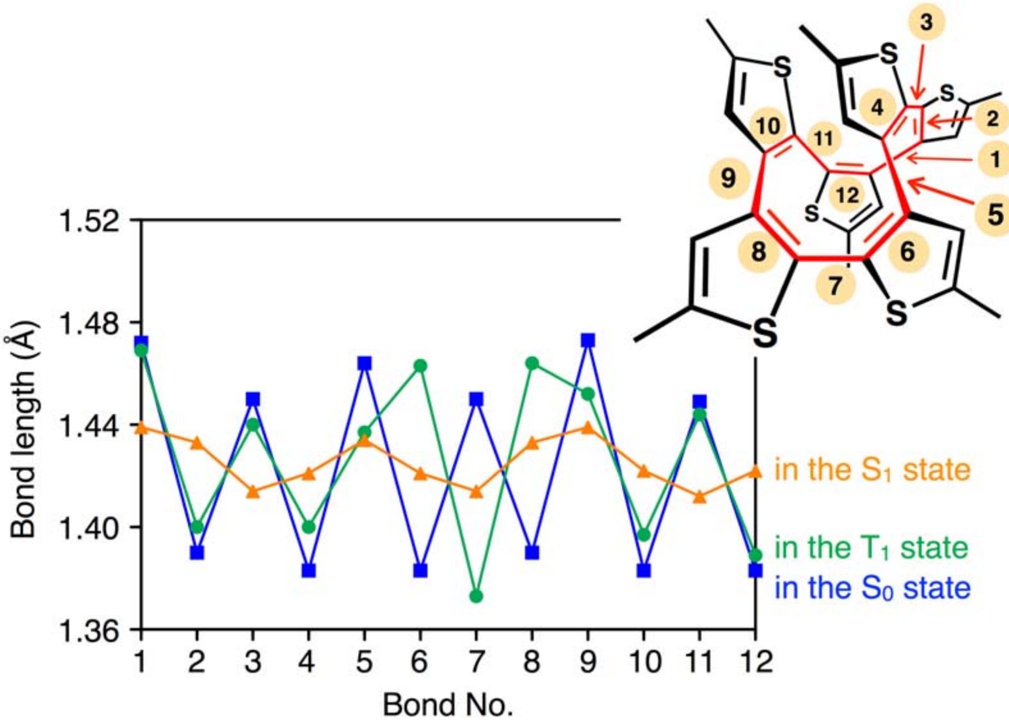

Supplementary Figure 43 | BLA of the minimum energy structures of ^{Th6}CDH$_{Screw}$ in the S_0, T_1 and S_1 states. The lengths of the C–C bonds in the cyclododecahexaene ring ([12]annulene), indexed using numerals in the inset figure, are plotted for the minimum energy structure in S_0, T_1 and S_1 states as blue, green and orange lines, respectively. The minimum energy structure in the S_1 state stands out with a lower BLA, indicating that it might have some extent of aromaticity. This was supported by the even distribution of dihedral angles of the core [12] annulene for the minimum energy structure in the S_1 state compared to the two other electronic states (Supplementary Fig. 45).

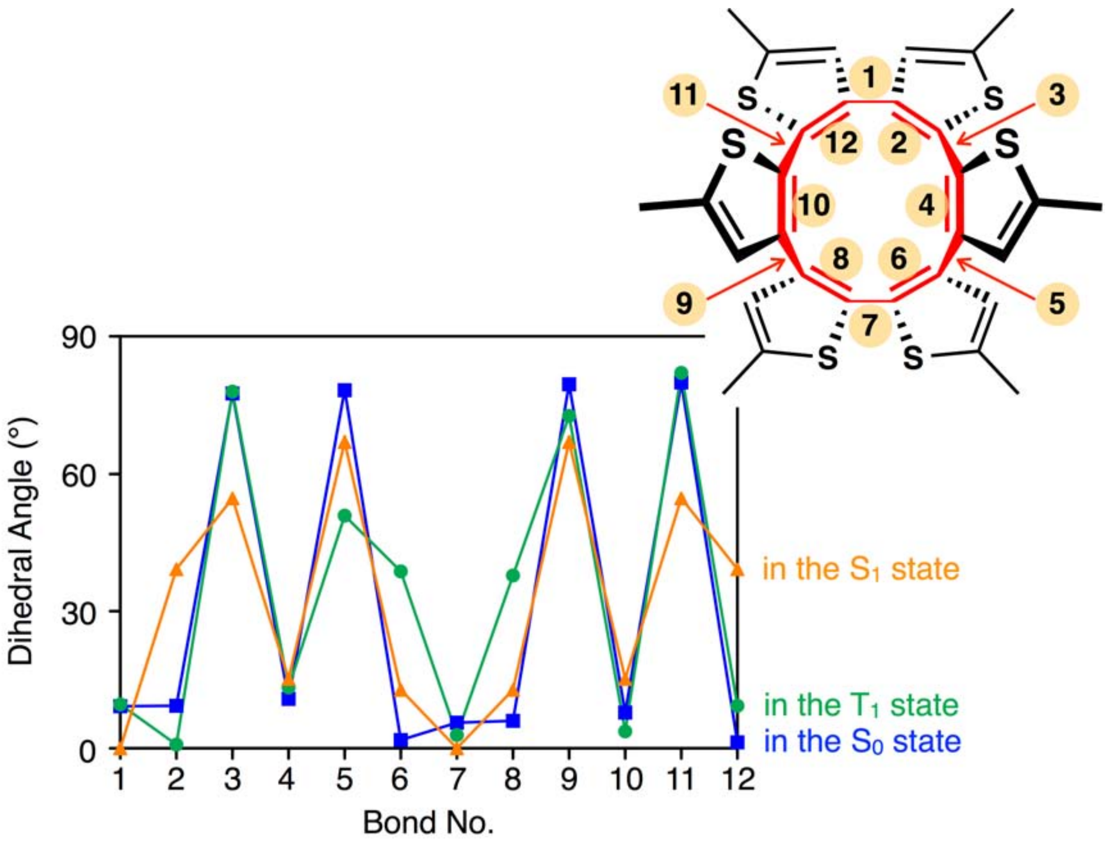

Supplementary Figure 44 | Dihedral angles of the transition state structures of ^{Th6}CDH$_{Screw}$ in the S_0, T_1 and S_1 states. The dihedral angles of the four continuous carbon atoms in the core [12]annulene, whose middle bond is indexed as the inset figure, are plotted for the transition state structures in the S_0, T_1 and S_1 states as blue, green and orange lines, respectively. All the transition state structures have multiple large dihedral angles of ca. 70° or more, indicating the weak or non-existent cyclic conjugation.

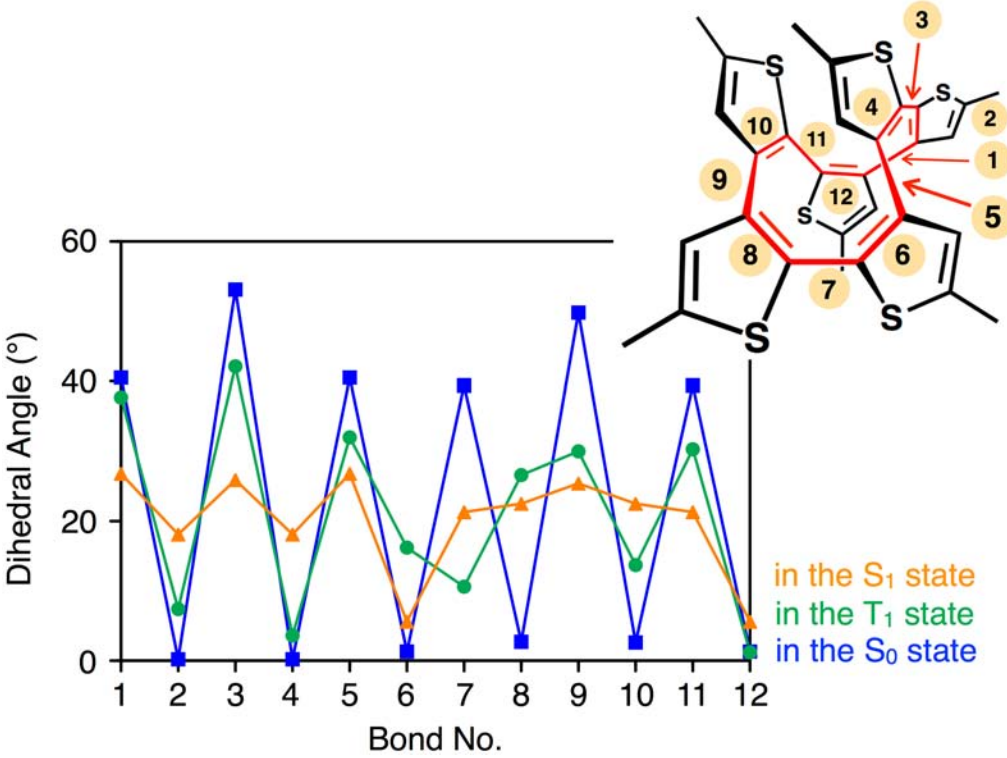

Supplementary Figure 45 | Dihedral angles of the minimum energy structures of ^{Th6}CDH$_{Screw}$ in the S_0, T_1 and S_1 states. The dihedral angles of the four continuous carbon atoms in the core [12]annulene, whose middle bond is indexed as the inset figure, are plotted for the minimum energy structure in the S_0, T_1 and S_1 states as blue, green and orange lines, respectively. The minimum energy structure in the S_1 state has a more even distribution of dihedral angles than those of the two other electronic states, indicating possible aromatic character. This is consistent with the lower BLA result for the minimum energy structure in the S_1 state (Supplementary Fig. 43).

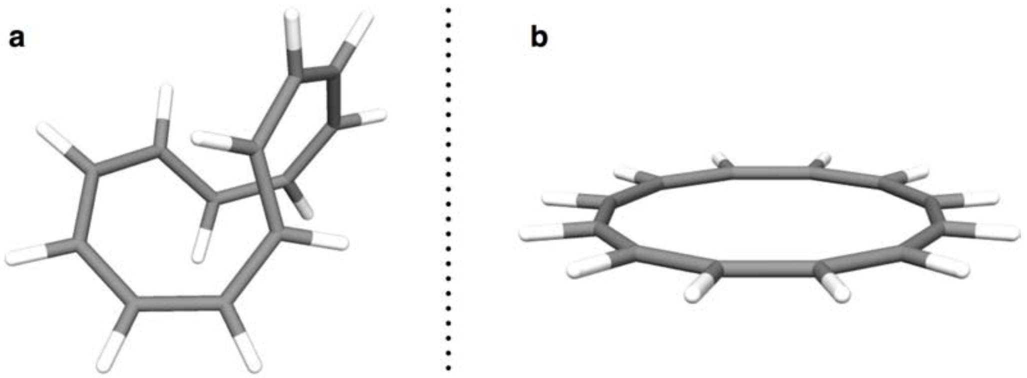

Supplementary Figure 46 | Energetics of [12]annulenes. a, Twisted C_2-symmetric conformation of [12]annulenes. **b,** Planar D_{12h}-symmetric conformation of [12]annulenes.

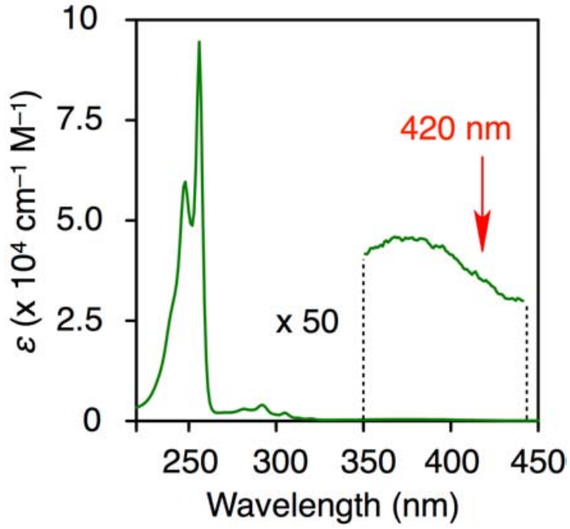

Supplementary Figure 47 | Electronic absorption spectrum of fluorenone at 25 °C in methylcyclohexane.

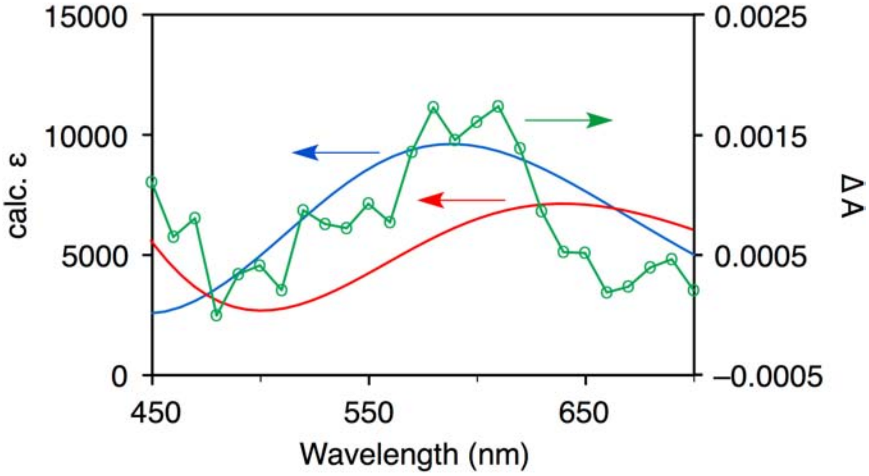

Supplementary Figure 48 | Calculated triplet-triplet vertical excitation spectra. The spectra calculated at the TD-CAM-B3LYP and TD-B3LYP levels of theory with the 6-311+G(2d,p) basis set are shown as blue and red lines, respectively. These spectra match the ns-TAS of $^{\mathbf{Th4}}\mathbf{COT}_{\mathbf{Saddle}}$ in the presence of fluorenone (green line; the spectrum at 2.0 μs in Supplementary Fig. 23a). This supports the formation of the T_1 state of $^{\mathbf{Th4}}\mathbf{COT}_{\mathbf{Saddle}}$ under sensitized conditions.

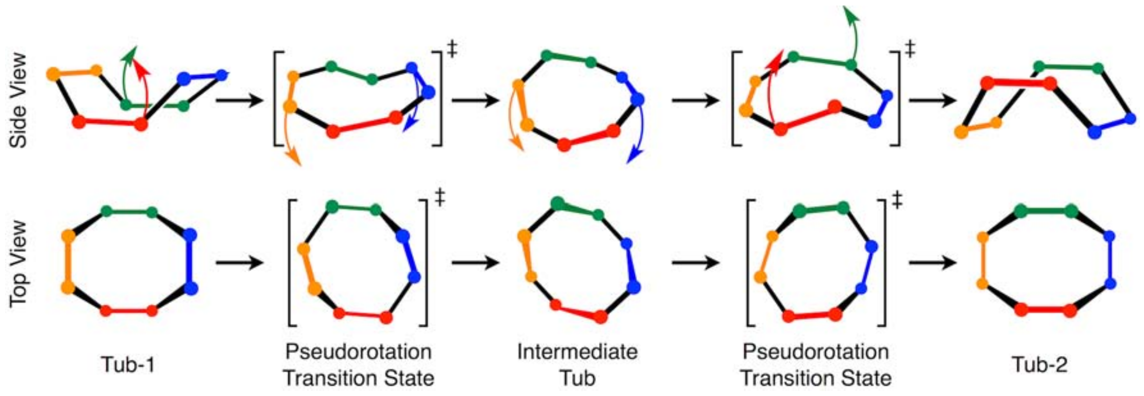

Supplementary Figure 49 | Pseudorotation of COT. Schematic illustrations of the process of pseudorotation of COT from one tub conformer (Tub-1) to the other (Tub-2). The coloured arrows represent the motion of the corresponding coloured carbon atoms during the pseudorotation.

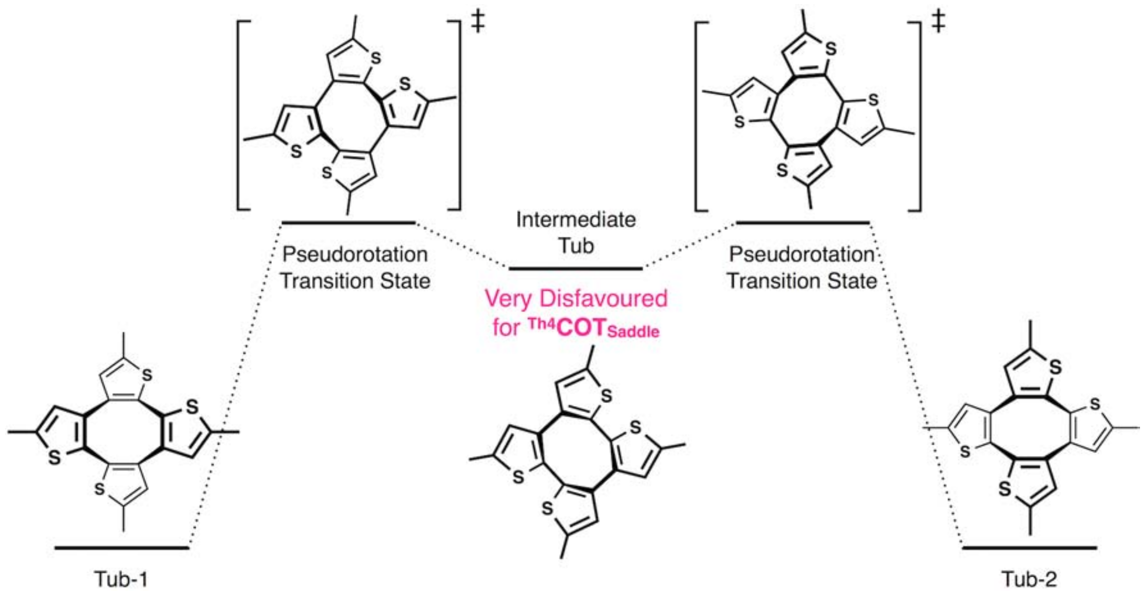

Supplementary Figure 50 | Hypothetical energy diagram for pseudorotation of ^{Th4}COT$_{Saddle}$. Schematic illustrations of a hypothetical energy diagram for pseudorotation process of **^{Th4}COT$_{Saddle}$** from one saddle enantiomer (Tub-1) to the mirror image (Tub-2). The intermediate tub conformer of **^{Th4}COT$_{Saddle}$** is very disfavoured due to steric repulsion of fused thiophene rings.

a) S_0 state

Symmetry	Relative electronic energy (kcal mol^{-1})	No. of imaginary frequencies
C_i	0.00	1
D_{2h}	0.82	3

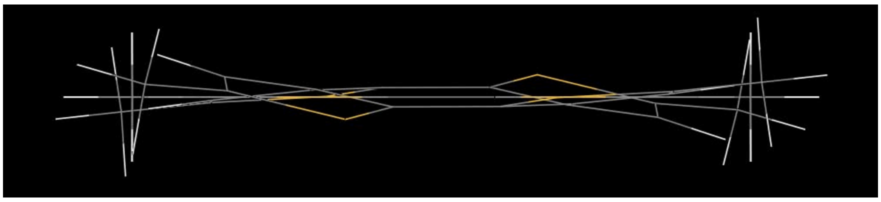

b) T_1 state

Symmetry	Relative electronic energy (kcal mol^{-1})	No. of imaginary frequencies
C_s	0.00	1
C_{2v}	0.09	3
C_{2h}	0.09	2
D_{2h}	0.46	5

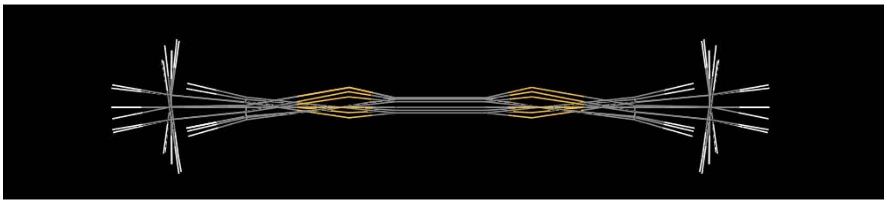

c) S_1 state

Symmetry	Relative electronic energy (kcal mol^{-1})	No. of imaginary frequencies
C_s	0.00	1
C_{2h}	0.30	2
D_{2h}	0.65	5

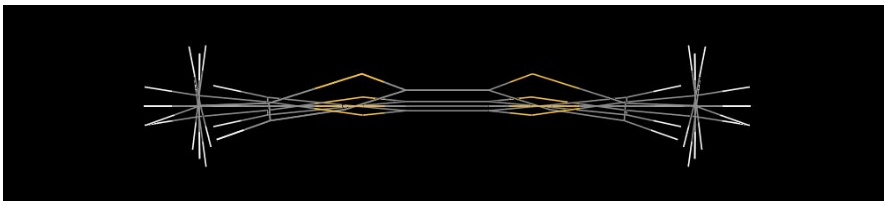

Supplementary Figure 51 | Optimized saddle points of different order near the planar geometry of $^{Th4}COT_{Saddle}$. Figures below each table shows an overlay of the optimized saddle points for the corresponding electronic states.

Supplementary Note 1

Calculated Atomic Coordinates

Energies are given for the B3LYP-D3(BJ)/6-31G(d) level of optimization for the singlet ground state and triplet states and the TD-B3LYP-D3(BJ)/6-31+G(d,p) level of optimization for the singlet excited states.

$^{Th4}COT_{Saddle}$: minimum energy in S_0 state
Symmetry: D_2
Energy: -2364.63372020

C	-0.627810	1.636175	0.382973
C	-1.567722	0.626037	0.369551
C	-1.040572	2.754559	1.181310
H	-0.424369	3.637692	1.315307
C	-2.265865	2.608136	1.769291
S	-2.958284	1.059262	1.355384
C	0.627810	1.636175	-0.382973
C	1.567722	0.626037	-0.369551
C	1.040572	2.754559	-1.181310
H	0.424369	3.637692	-1.315307
C	2.265865	2.608136	-1.769291
S	2.958284	1.059262	-1.355384
C	0.627810	-1.636175	0.382973
C	1.567722	-0.626037	0.369551
C	1.040572	-2.754559	1.181310
H	0.424369	-3.637692	1.315307
C	2.265865	-2.608136	1.769291
S	2.958284	-1.059262	1.355384
C	-0.627810	-1.636175	-0.382973
C	-1.567722	-0.626037	-0.369551
C	-1.040572	-2.754559	-1.181310
H	-0.424369	-3.637692	-1.315307
C	-2.265865	-2.608136	-1.769291
S	-2.958284	-1.059262	-1.355384
C	2.984528	-3.566099	2.668597
H	2.394752	-4.481097	2.780114
H	3.145350	-3.145185	3.668494
H	3.966724	-3.843281	2.267321
C	2.984528	3.566099	-2.668597
H	2.394752	4.481097	-2.780114
H	3.145350	3.145185	-3.668494
H	3.966724	3.843281	-2.267321
C	-2.984528	3.566099	2.668597
H	-3.966724	3.843281	2.267321
H	-2.394752	4.481097	2.780114
H	-3.145350	3.145185	3.668494
C	-2.984528	-3.566099	-2.668597
H	-3.966724	-3.843281	-2.267321
H	-2.394752	-4.481097	-2.780114
H	-3.145350	-3.145185	-3.668494

Bq atom coordinates for NICS scan:

bq	0.000000	0.000000	0.000000
bq	0.000000	0.000000	0.100000
bq	0.000000	0.000000	0.200000
bq	0.000000	0.000000	0.300000
bq	0.000000	0.000000	0.400000
bq	0.000000	0.000000	0.500000
bq	0.000000	0.000000	0.600000
bq	0.000000	0.000000	0.700000
bq	0.000000	0.000000	0.800000
bq	0.000000	0.000000	0.900000
bq	0.000000	0.000000	1.000000
bq	0.000000	0.000000	1.100000
bq	0.000000	0.000000	1.200000
bq	0.000000	0.000000	1.300000
bq	0.000000	0.000000	1.400000
bq	0.000000	0.000000	1.500000
bq	0.000000	0.000000	1.600000
bq	0.000000	0.000000	1.700000
bq	0.000000	0.000000	1.800000
bq	0.000000	0.000000	1.900000
bq	0.000000	0.000000	2.000000
bq	0.000000	0.000000	2.100000
bq	0.000000	0.000000	2.200000
bq	0.000000	0.000000	2.300000
bq	0.000000	0.000000	2.400000
bq	0.000000	0.000000	2.500000
bq	0.000000	0.000000	2.600000
bq	0.000000	0.000000	2.700000
bq	0.000000	0.000000	2.800000
bq	0.000000	0.000000	2.900000
bq	0.000000	0.000000	3.000000
bq	0.000000	0.000000	3.100000
bq	0.000000	0.000000	3.200000
bq	0.000000	0.000000	3.300000
bq	0.000000	0.000000	3.400000
bq	0.000000	0.000000	3.500000
bq	0.000000	0.000000	3.600000
bq	0.000000	0.000000	3.700000
bq	0.000000	0.000000	3.800000
bq	0.000000	0.000000	3.900000
bq	0.000000	0.000000	4.000000
bq	0.000000	0.000000	4.100000
bq	0.000000	0.000000	4.200000
bq	0.000000	0.000000	4.300000

bq	0.000000	0.000000	4.400000
bq	0.000000	0.000000	4.500000
bq	0.000000	0.000000	4.600000
bq	0.000000	0.000000	4.700000
bq	0.000000	0.000000	4.800000
bq	0.000000	0.000000	4.900000

^{Th4}COT$_{Saddle}$: transition state in S_0 state

Symmetry: C_i

Energy: -2364.58672639

C	0.822710	1.708460	0.104670
C	1.712370	0.663500	-0.134760
C	1.530800	2.958240	0.277280
H	1.049710	3.876500	0.580550
C	2.876160	2.921090	0.086140
S	3.360550	1.309400	-0.305760
C	-0.661650	1.777630	0.094990
C	-1.653300	0.801430	0.129550
C	-1.239690	3.097540	-0.040130
H	-0.652690	3.995680	-0.159610
C	-2.596800	3.162120	-0.076690
S	-3.254530	1.568630	0.008410
C	-0.822710	-1.708460	-0.104670
C	-1.712370	-0.663500	0.134760
C	-1.530800	-2.958240	-0.277280
H	-1.049710	-3.876500	-0.580550
C	-2.876160	-2.921090	-0.086140
S	-3.360550	-1.309400	0.305760
C	0.661650	-1.777630	-0.094990
C	1.653300	-0.801430	-0.129550
C	1.239690	-3.097540	0.040130
H	0.652690	-3.995680	0.159610
C	2.596800	-3.162120	0.076690
S	3.254530	-1.568630	-0.008410
C	-3.861720	-4.043820	-0.173910
H	-3.344310	-4.969480	-0.443910
H	-4.632540	-3.852720	-0.930100
H	-4.373920	-4.210710	0.781550
C	-3.468310	4.372280	-0.203040
H	-2.846350	5.267260	-0.301230
H	-4.111480	4.503280	0.675730
H	-4.121560	4.315300	-1.081960
C	3.861720	4.043820	0.173910
H	4.632540	3.852720	0.930100
H	3.344310	4.969480	0.443910
H	4.373920	4.210710	-0.781550
C	3.468310	-4.372280	0.203040
H	4.111480	-4.503280	-0.675730
H	2.846350	-5.267260	0.301230
H	4.121560	-4.315300	1.081960

Bq atom coordinates for NICS scan:

bq	0.000000	0.000000	0.000000
bq	0.000000	0.000000	0.100000
bq	0.000000	0.000000	0.200000
bq	0.000000	0.000000	0.300000
bq	0.000000	0.000000	0.400000
bq	0.000000	0.000000	0.500000
bq	0.000000	0.000000	0.600000
bq	0.000000	0.000000	0.700000
bq	0.000000	0.000000	0.800000
bq	0.000000	0.000000	0.900000
bq	0.000000	0.000000	1.000000
bq	0.000000	0.000000	1.100000
bq	0.000000	0.000000	1.200000
bq	0.000000	0.000000	1.300000
bq	0.000000	0.000000	1.400000
bq	0.000000	0.000000	1.500000
bq	0.000000	0.000000	1.600000
bq	0.000000	0.000000	1.700000
bq	0.000000	0.000000	1.800000
bq	0.000000	0.000000	1.900000
bq	0.000000	0.000000	2.000000
bq	0.000000	0.000000	2.100000
bq	0.000000	0.000000	2.200000
bq	0.000000	0.000000	2.300000
bq	0.000000	0.000000	2.400000
bq	0.000000	0.000000	2.500000
bq	0.000000	0.000000	2.600000
bq	0.000000	0.000000	2.700000
bq	0.000000	0.000000	2.800000
bq	0.000000	0.000000	2.900000
bq	0.000000	0.000000	3.000000
bq	0.000000	0.000000	3.100000
bq	0.000000	0.000000	3.200000
bq	0.000000	0.000000	3.300000
bq	0.000000	0.000000	3.400000
bq	0.000000	0.000000	3.500000
bq	0.000000	0.000000	3.600000
bq	0.000000	0.000000	3.700000
bq	0.000000	0.000000	3.800000
bq	0.000000	0.000000	3.900000
bq	0.000000	0.000000	4.000000
bq	0.000000	0.000000	4.100000
bq	0.000000	0.000000	4.200000
bq	0.000000	0.000000	4.300000
bq	0.000000	0.000000	4.400000
bq	0.000000	0.000000	4.500000
bq	0.000000	0.000000	4.600000
bq	0.000000	0.000000	4.700000
bq	0.000000	0.000000	4.800000
bq	0.000000	0.000000	4.900000

^{Th4}COT$_{Saddle}$: minimum energy in T_1 state

Symmetry: D_2

Energy: -2364.56733458

C	-0.665310	1.701215	0.259819
C	-1.646728	0.655518	0.251215
C	-1.231430	2.932605	0.746712
H	-0.635910	3.828145	0.879193
C	-2.546070	2.892358	1.089993

S	-3.211277	1.291146	0.831846
C	0.665310	1.701215	-0.259819
C	1.646728	0.655518	-0.251215
C	1.231430	2.932605	-0.746712
H	0.635910	3.828145	-0.879193
C	2.546070	2.892358	-1.089993
S	3.211277	1.291146	-0.831846
C	0.665310	-1.701215	0.259819
C	1.646728	-0.655518	0.251215
C	1.231430	-2.932605	0.746712
H	0.635910	-3.828145	0.879193
C	2.546070	-2.892358	1.089993
S	3.211277	-1.291146	0.831846
C	-0.665310	-1.701215	-0.259819
C	-1.646728	-0.655518	-0.251215
C	-1.231430	-2.932605	-0.746712
H	-0.635910	-3.828145	-0.879193
C	-2.546070	-2.892358	-1.089993
S	-3.211277	-1.291146	-0.831846
C	3.389359	-3.984665	1.663571
H	2.801132	-4.902593	1.757101
H	3.771808	-3.722128	2.658067
H	4.257633	-4.198648	1.027766
C	3.389359	3.984665	-1.663571
H	2.801132	4.902593	-1.757101
H	3.771808	3.722128	-2.658067
H	4.257633	4.198648	-1.027766
C	-3.389359	3.984665	1.663571
H	-4.257633	4.198648	1.027766
H	-2.801132	4.902593	1.757101
H	-3.771808	3.722128	2.658067
C	-3.389359	-3.984665	-1.663571
H	-4.257633	-4.198648	-1.027766
H	-2.801132	-4.902593	-1.757101
H	-3.771808	-3.722128	-2.658067

Bq atom coordinates for NICS scan:

bq	0.000000	0.000000	0.000000
bq	0.000000	0.000000	0.100000
bq	0.000000	0.000000	0.200000
bq	0.000000	0.000000	0.300000
bq	0.000000	0.000000	0.400000
bq	0.000000	0.000000	0.500000
bq	0.000000	0.000000	0.600000
bq	0.000000	0.000000	0.700000
bq	0.000000	0.000000	0.800000
bq	0.000000	0.000000	0.900000
bq	0.000000	0.000000	1.000000
bq	0.000000	0.000000	1.100000
bq	0.000000	0.000000	1.200000
bq	0.000000	0.000000	1.300000
bq	0.000000	0.000000	1.400000
bq	0.000000	0.000000	1.500000
bq	0.000000	0.000000	1.600000
bq	0.000000	0.000000	1.700000
bq	0.000000	0.000000	1.800000
bq	0.000000	0.000000	1.900000
bq	0.000000	0.000000	2.000000
bq	0.000000	0.000000	2.100000
bq	0.000000	0.000000	2.200000
bq	0.000000	0.000000	2.300000
bq	0.000000	0.000000	2.400000
bq	0.000000	0.000000	2.500000
bq	0.000000	0.000000	2.600000
bq	0.000000	0.000000	2.700000
bq	0.000000	0.000000	2.800000
bq	0.000000	0.000000	2.900000
bq	0.000000	0.000000	3.000000
bq	0.000000	0.000000	3.100000
bq	0.000000	0.000000	3.200000
bq	0.000000	0.000000	3.300000
bq	0.000000	0.000000	3.400000
bq	0.000000	0.000000	3.500000
bq	0.000000	0.000000	3.600000
bq	0.000000	0.000000	3.700000
bq	0.000000	0.000000	3.800000
bq	0.000000	0.000000	3.900000
bq	0.000000	0.000000	4.000000
bq	0.000000	0.000000	4.100000
bq	0.000000	0.000000	4.200000
bq	0.000000	0.000000	4.300000
bq	0.000000	0.000000	4.400000
bq	0.000000	0.000000	4.500000
bq	0.000000	0.000000	4.600000
bq	0.000000	0.000000	4.700000
bq	0.000000	0.000000	4.800000
bq	0.000000	0.000000	4.900000

$^{Th4}COT_{Saddle}$: transition state in T_1 state

Symmetry: C_s

Energy: -2364.55225327

C	-1.755270	-0.716010	-0.050850
C	-0.703290	-1.691180	0.102360
C	-3.030040	-1.383530	-0.216070
H	-3.938610	-0.860610	-0.474860
C	-3.039340	-2.732070	-0.092170
S	-1.434010	-3.332430	0.217470
C	-1.759290	0.716980	-0.011310
C	-0.705130	1.696520	-0.070900
C	-3.045950	1.375190	0.097910
H	-3.968480	0.839740	0.264810
C	-3.050440	2.728320	0.057460
S	-1.430290	3.342230	-0.107360
C	1.759290	0.716980	-0.011310
C	0.705130	1.696520	-0.070900
C	3.045950	1.375190	0.097910
H	3.968480	0.839740	0.264800
C	3.050440	2.728320	0.057460
S	1.430290	3.342230	-0.107360
C	1.755270	-0.716010	-0.050850
C	0.703290	-1.691180	0.102360

C	3.030040	-1.383530	-0.216070
H	3.938610	-0.860610	-0.474860
C	3.039340	-2.732070	-0.092180
S	1.434010	-3.332430	0.217470
C	4.216940	3.656200	0.164150
H	5.142780	3.083270	0.272590
H	4.124060	4.324350	1.029330
H	4.310480	4.289080	-0.727170
C	-4.216940	3.656200	0.164150
H	-5.142780	3.083270	0.272590
H	-4.310480	4.289080	-0.727170
H	-4.124060	4.324350	1.029340
C	-4.198740	-3.666800	-0.212980
H	-4.052550	-4.389670	-1.025190
H	-5.114630	-3.103860	-0.416640
H	-4.349910	-4.240780	0.709840
C	4.198740	-3.666800	-0.212980
H	4.349910	-4.240780	0.709840
H	5.114630	-3.103860	-0.416640
H	4.052550	-4.389670	-1.025190

Bq atom coordinates for NICS scan:

bq	0.000000	0.000000	0.000000
bq	0.000000	0.000000	0.100000
bq	0.000000	0.000000	0.200000
bq	0.000000	0.000000	0.300000
bq	0.000000	0.000000	0.400000
bq	0.000000	0.000000	0.500000
bq	0.000000	0.000000	0.600000
bq	0.000000	0.000000	0.700000
bq	0.000000	0.000000	0.800000
bq	0.000000	0.000000	0.900000
bq	0.000000	0.000000	1.000000
bq	0.000000	0.000000	1.100000
bq	0.000000	0.000000	1.200000
bq	0.000000	0.000000	1.300000
bq	0.000000	0.000000	1.400000
bq	0.000000	0.000000	1.500000
bq	0.000000	0.000000	1.600000
bq	0.000000	0.000000	1.700000
bq	0.000000	0.000000	1.800000
bq	0.000000	0.000000	1.900000
bq	0.000000	0.000000	2.000000
bq	0.000000	0.000000	2.100000
bq	0.000000	0.000000	2.200000
bq	0.000000	0.000000	2.300000
bq	0.000000	0.000000	2.400000
bq	0.000000	0.000000	2.500000
bq	0.000000	0.000000	2.600000
bq	0.000000	0.000000	2.700000
bq	0.000000	0.000000	2.800000
bq	0.000000	0.000000	2.900000
bq	0.000000	0.000000	3.000000
bq	0.000000	0.000000	3.100000
bq	0.000000	0.000000	3.200000
bq	0.000000	0.000000	3.300000
bq	0.000000	0.000000	3.400000
bq	0.000000	0.000000	3.500000
bq	0.000000	0.000000	3.600000
bq	0.000000	0.000000	3.700000
bq	0.000000	0.000000	3.800000
bq	0.000000	0.000000	3.900000
bq	0.000000	0.000000	4.000000
bq	0.000000	0.000000	4.100000
bq	0.000000	0.000000	4.200000
bq	0.000000	0.000000	4.300000
bq	0.000000	0.000000	4.400000
bq	0.000000	0.000000	4.500000
bq	0.000000	0.000000	4.600000
bq	0.000000	0.000000	4.700000
bq	0.000000	0.000000	4.800000
bq	0.000000	0.000000	4.900000

^{Th4}COT$_{Saddle}$: minimum energy in S$_1$ state

Symmetry: D_2

Energy: -2364.59880632

C	0.666665	0.255025	1.707634
C	1.648793	0.249807	0.657431
C	1.239794	0.736754	2.939818
C	2.553129	1.081775	2.899784
C	-0.666665	-0.255025	1.707634
C	-1.648793	-0.249807	0.657431
C	-1.239794	-0.736754	2.939818
C	-2.553129	-1.081775	2.899784
C	-0.666631	0.255074	-1.707043
C	-1.648750	0.250216	-0.656823
C	-1.239705	0.736449	-2.939357
C	-2.553042	1.081498	-2.899415
C	0.666631	-0.255074	-1.707043
C	1.648750	-0.250216	-0.656823
C	1.239705	-0.736449	-2.939357
C	2.553042	-1.081498	-2.899415
C	-3.398847	1.649042	-3.995458
C	-3.398979	-1.649536	3.995680
C	3.398979	1.649536	3.995680
C	3.398847	-1.649042	-3.995458
S	3.210679	0.833132	1.294410
S	-3.210679	-0.833132	1.294410
S	-3.210640	0.833269	-1.294028
S	3.210640	-0.833269	-1.294028
H	0.647132	0.866753	3.838148
H	-0.647132	-0.866753	3.838148
H	-0.647170	0.865834	-3.837873
H	0.647170	-0.865834	-3.837873
H	-2.812168	1.736379	-4.915282
H	-3.781025	2.646104	-3.740962
H	-4.268468	1.013296	-4.205975
H	-2.812036	-1.738021	4.915221
H	-3.782108	-2.646084	3.740621
H	-4.267984	-1.013193	4.206964
H	4.267984	1.013193	4.206964

H	2.812036	1.738021	4.915221
H	3.782108	2.646084	3.740621
H	4.268468	-1.013296	-4.205975
H	2.812168	-1.736379	-4.915282
H	3.781025	-2.646104	-3.740962

Bq atom coordinates for NICS scan:

bq	0.000000	0.000000	0.000000
bq	0.000000	0.100000	0.000000
bq	0.000000	0.200000	0.000000
bq	0.000000	0.300000	0.000000
bq	0.000000	0.400000	0.000000
bq	0.000000	0.500000	0.000000
bq	0.000000	0.600000	0.000000
bq	0.000000	0.700000	0.000000
bq	0.000000	0.800000	0.000000
bq	0.000000	0.900000	0.000000
bq	0.000000	1.000000	0.000000
bq	0.000000	1.100000	0.000000
bq	0.000000	1.200000	0.000000
bq	0.000000	1.300000	0.000000
bq	0.000000	1.400000	0.000000
bq	0.000000	1.500000	0.000000
bq	0.000000	1.600000	0.000000
bq	0.000000	1.700000	0.000000
bq	0.000000	1.800000	0.000000
bq	0.000000	1.900000	0.000000
bq	0.000000	2.000000	0.000000
bq	0.000000	2.100000	0.000000
bq	0.000000	2.200000	0.000000
bq	0.000000	2.300000	0.000000
bq	0.000000	2.400000	0.000000
bq	0.000000	2.500000	0.000000
bq	0.000000	2.600000	0.000000
bq	0.000000	2.700000	0.000000
bq	0.000000	2.800000	0.000000
bq	0.000000	2.900000	0.000000
bq	0.000000	3.000000	0.000000
bq	0.000000	3.100000	0.000000
bq	0.000000	3.200000	0.000000
bq	0.000000	3.300000	0.000000
bq	0.000000	3.400000	0.000000
bq	0.000000	3.500000	0.000000
bq	0.000000	3.600000	0.000000
bq	0.000000	3.700000	0.000000
bq	0.000000	3.800000	0.000000
bq	0.000000	3.900000	0.000000
bq	0.000000	4.000000	0.000000
bq	0.000000	4.100000	0.000000
bq	0.000000	4.200000	0.000000
bq	0.000000	4.300000	0.000000
bq	0.000000	4.400000	0.000000
bq	0.000000	4.500000	0.000000
bq	0.000000	4.600000	0.000000
bq	0.000000	4.700000	0.000000
bq	0.000000	4.800000	0.000000
bq	0.000000	4.900000	0.000000
bq	0.000000	5.000000	0.000000

^{Th4}COT$_{Saddle}$: transition state in S_1 state

Symmetry: C_s

Energy: -2364.58394641

C	0.209163	-0.713191	1.745301
C	-0.074483	-1.674136	0.703934
C	0.353261	-1.393145	3.020030
C	0.093041	-2.722660	3.035747
C	0.218147	0.712999	1.737858
C	-0.119915	1.663899	0.702843
C	0.430206	1.408955	2.994318
C	0.128602	2.729789	3.017650
C	0.218147	0.712999	-1.737858
C	-0.119915	1.663899	-0.702843
C	0.430206	1.408955	-2.994318
C	0.128602	2.729789	-3.017650
C	0.209163	-0.713191	-1.745301
C	-0.074483	-1.674136	-0.703934
C	0.353261	-1.393145	-3.020030
C	0.093041	-2.722660	-3.035747
C	0.194434	3.676935	-4.171819
C	0.194434	3.676935	4.171819
C	0.102253	-3.656472	4.202719
C	0.102253	-3.656472	-4.202719
S	-0.318599	-3.292300	1.442133
S	-0.403041	3.273108	1.448791
S	-0.403041	3.273108	-1.448791
S	-0.318599	-3.292300	-1.442133
H	0.602696	-0.888267	3.939957
H	0.787785	0.925504	3.890260
H	0.787785	0.925504	-3.890260
H	0.602696	-0.888267	-3.939957
H	0.536031	3.150954	-5.067519
H	0.885556	4.505005	-3.975075
H	-0.787942	4.114527	-4.385897
H	0.536031	3.150954	5.067519
H	-0.787942	4.114527	4.385897
H	0.885556	4.505005	3.975075
H	-0.880717	-4.118402	4.353394
H	0.367463	-3.112849	5.113607
H	0.827119	-4.467242	4.063927
H	0.827119	-4.467242	-4.063927
H	0.367463	-3.112849	-5.113607
H	-0.880717	-4.118402	-4.353394

Bq atom coordinates for NICS scan:

bq	0.000000	0.000000	0.000000
bq	0.100000	0.000000	0.000000
bq	0.200000	0.000000	0.000000
bq	0.300000	0.000000	0.000000
bq	0.400000	0.000000	0.000000
bq	0.500000	0.000000	0.000000
bq	0.600000	0.000000	0.000000
bq	0.700000	0.000000	0.000000

bq	0.800000	0.000000	0.000000
bq	0.900000	0.000000	0.000000
bq	1.000000	0.000000	0.000000
bq	1.100000	0.000000	0.000000
bq	1.200000	0.000000	0.000000
bq	1.300000	0.000000	0.000000
bq	1.400000	0.000000	0.000000
bq	1.500000	0.000000	0.000000
bq	1.600000	0.000000	0.000000
bq	1.700000	0.000000	0.000000
bq	1.800000	0.000000	0.000000
bq	1.900000	0.000000	0.000000
bq	2.000000	0.000000	0.000000
bq	2.100000	0.000000	0.000000
bq	2.200000	0.000000	0.000000
bq	2.300000	0.000000	0.000000
bq	2.400000	0.000000	0.000000
bq	2.500000	0.000000	0.000000
bq	2.600000	0.000000	0.000000
bq	2.700000	0.000000	0.000000
bq	2.800000	0.000000	0.000000
bq	2.900000	0.000000	0.000000
bq	3.000000	0.000000	0.000000
bq	3.100000	0.000000	0.000000
bq	3.200000	0.000000	0.000000
bq	3.300000	0.000000	0.000000
bq	3.400000	0.000000	0.000000
bq	3.500000	0.000000	0.000000
bq	3.600000	0.000000	0.000000
bq	3.700000	0.000000	0.000000
bq	3.800000	0.000000	0.000000
bq	3.900000	0.000000	0.000000
bq	4.000000	0.000000	0.000000
bq	4.100000	0.000000	0.000000
bq	4.200000	0.000000	0.000000
bq	4.300000	0.000000	0.000000
bq	4.400000	0.000000	0.000000
bq	4.500000	0.000000	0.000000
bq	4.600000	0.000000	0.000000
bq	4.700000	0.000000	0.000000
bq	4.800000	0.000000	0.000000
bq	4.900000	0.000000	0.000000
bq	5.000000	0.000000	0.000000

$^{Th4}COT_{Saddle}$: transition state in S_1 state

Symmetry: D_{2h}

CASSCF energy: -2356.603025

C	-0.709827	1.751484	-0.001761
C	-1.687584	0.703955	-0.004073
C	-1.388935	3.050711	0.000217
H	-0.878793	3.985257	0.011209
C	-2.716265	3.040453	-0.008608
S	-3.321511	1.425463	-0.020430
C	0.710201	1.751335	0.001124
C	1.687738	0.703610	0.006407
C	1.389578	3.050412	-0.003902
H	0.879649	3.985032	-0.018195
C	2.716891	3.039913	0.006498
S	3.321776	1.424819	0.022660
C	0.709830	-1.751515	-0.002246
C	1.687595	-0.703989	-0.002696
C	1.388923	-3.050745	-0.002438
H	0.878772	-3.985313	0.005578
C	2.716262	-3.040484	-0.009840
S	3.321493	-1.425483	-0.016114
C	-0.710196	-1.751362	0.000708
C	-1.687716	-0.703628	0.006790
C	-1.389610	-3.050419	-0.004860
H	-0.879708	-3.985042	-0.019938
C	-2.716923	-3.039874	0.006135
S	-3.321696	-1.424761	0.025230
C	3.650089	-4.212239	-0.011030
H	3.087688	-5.138126	-0.004228
H	4.295305	-4.198979	0.861816
H	4.285737	-4.205420	-0.890887
C	3.650974	4.211477	0.003565
H	3.088742	5.137481	0.006182
H	4.288402	4.201567	-0.875017
H	4.294397	4.201040	0.877711
C	-3.650105	4.212198	-0.009258
H	-4.283298	4.207272	-0.890931
H	-3.087748	5.138083	0.000780
H	-4.297748	4.197037	0.861721
C	-3.651047	-4.211409	0.004136
H	-4.289958	-4.203524	0.881644
H	-3.088833	-5.137424	0.001256
H	-4.292990	-4.198913	-0.871091

$^{Th6}CDH_{Screw}$: minimum energy in S_0 state

Symmetry: C_2

Energy: -3546.97176826

C	-1.253075	-1.378922	1.075643
C	-0.141040	-0.710493	1.555582
C	-1.146129	-2.799098	1.275678
H	-1.896608	-3.498194	0.922321
C	0.008602	-3.201713	1.883509
S	1.011714	-1.826643	2.263529
C	1.253130	1.378905	1.075588
C	0.141118	0.710472	1.555577
C	1.146197	2.799079	1.275646
H	1.896663	3.498178	0.922266
C	-0.008496	3.201688	1.883551
S	-1.011613	1.826621	2.263563
C	2.444378	0.792125	0.438788
C	2.519130	-0.248561	-0.479902
C	3.746643	1.312598	0.759498
H	3.888387	2.114120	1.476828
C	4.790008	0.684558	0.143786
S	4.197983	-0.576836	-0.905449
C	0.250178	-0.687929	-1.595388

C	1.492353	-1.054898	-1.111897
C	-0.497692	-1.802781	-2.097309
H	-1.495977	-1.697426	-2.506787
C	0.156224	-2.998174	-2.018449
S	1.733807	-2.790231	-1.296866
C	-0.250263	0.687936	-1.595368
C	-1.492409	1.054904	-1.111803
C	0.497577	1.802789	-2.097331
H	1.495836	1.697434	-2.506873
C	-0.156329	2.998184	-2.018417
S	-1.733867	2.790240	-1.296735
C	-2.444356	-0.792134	0.438913
C	-2.519154	0.248564	-0.479759
C	-3.746606	-1.312601	0.759695
H	-3.888313	-2.114129	1.477026
C	-4.790003	-0.684542	0.144056
S	-4.198030	0.576858	-0.905200
C	0.474674	-4.598509	2.150861
H	-0.327049	-5.307978	1.922660
H	0.766969	-4.743904	3.197446
H	1.340480	-4.853186	1.526772
C	-6.255762	-0.951046	0.293991
H	-6.790150	-0.077087	0.685715
H	-6.411186	-1.780890	0.990191
H	-6.723038	-1.219645	-0.661257
C	-0.332379	-4.351698	-2.429053
H	-1.309809	-4.260021	-2.912380
H	-0.446142	-5.017403	-1.563938
H	0.350351	-4.841192	-3.133674
C	6.255774	0.951075	0.293631
H	6.411232	1.780915	0.989828
H	6.722987	1.219687	-0.661643
H	6.790194	0.077119	0.685316
C	-0.474536	4.598483	2.150970
H	-1.340376	4.853186	1.526939
H	0.327181	5.307948	1.922740
H	-0.766767	4.743853	3.197576
C	0.332251	4.351709	-2.429045
H	-0.350520	4.841206	-3.133624
H	1.309652	4.260032	-2.912430
H	0.446066	5.017412	-1.563934

^{Th6}CDH$_{Screw}$: transition state in S$_0$ state

Symmetry: C_1

Energy: -3546.93023267

C	-1.217600	1.882810	-1.003490
C	-2.040990	1.217690	-0.109280
C	-1.992140	2.391850	-2.115140
H	-1.563680	2.917450	-2.957440
C	-3.332500	2.151430	-2.060310
S	-3.721380	1.256930	-0.622760
C	-1.300250	-0.868240	1.114210
C	-1.757520	0.430180	1.081250
C	-1.229180	-1.381510	2.450340
H	-0.878120	-2.381620	2.679460
C	-1.663400	-0.505000	3.408260
S	-2.168610	1.001180	2.686000
C	-1.265210	-1.705840	-0.114100
C	-0.242860	-2.077170	-0.976400
C	-2.541930	-2.225300	-0.525790
H	-3.446050	-2.018580	0.036270
C	-2.529600	-2.973360	-1.664630
S	-0.907260	-3.076820	-2.282580
C	2.078850	-1.224090	-0.155900
C	1.194490	-1.838870	-1.024780
C	3.441640	-1.302050	-0.596080
H	4.253560	-0.858700	-0.029520
C	3.622880	-1.943630	-1.786150
S	2.089610	-2.488130	-2.409760
C	1.771130	-0.494810	1.087330
C	1.317790	0.806890	1.093820
C	2.083970	-0.985320	2.394520
H	2.434640	-1.995940	2.575930
C	1.890020	-0.069160	3.392050
S	1.309450	1.436080	2.733080
C	0.238420	2.161130	-0.932060
C	1.223740	1.710330	-0.060570
C	0.805970	3.068510	-1.907670
H	0.220980	3.568800	-2.666780
C	2.145350	3.294200	-1.806640
S	2.794990	2.392200	-0.477170
C	-4.382590	2.559070	-3.047630
H	-3.918550	3.091970	-3.883330
H	-5.128920	3.223870	-2.596220
H	-4.917800	1.693050	-3.455120
C	2.999530	4.174060	-2.666790
H	3.500840	4.953810	-2.081130
H	2.381650	4.665760	-3.424590
H	3.777790	3.601620	-3.185830
C	4.895090	-2.176880	-2.539250
H	5.735470	-1.753570	-1.980770
H	4.875380	-1.704500	-3.528980
H	5.092440	-3.245310	-2.689770
C	-3.675810	-3.639070	-2.359470
H	-4.600560	-3.449600	-1.806110
H	-3.538780	-4.725150	-2.428740
H	-3.808900	-3.259200	-3.379770
C	-1.739820	-0.717750	4.889050
H	-1.221640	0.073530	5.442950
H	-1.275960	-1.673870	5.150180
H	-2.777370	-0.737710	5.245280
C	2.073580	-0.256070	4.866370
H	1.119000	-0.216400	5.403410
H	2.726230	0.511390	5.298810
H	2.526880	-1.233080	5.061070

Bq atom coordinates for NICS scan:

bq	0.000000	0.000000	0.000000
bq	0.000000	0.000000	-0.100000
bq	0.000000	0.000000	-0.200000
bq	0.000000	0.000000	-0.300000

bq	0.000000	0.000000	-0.400000
bq	0.000000	0.000000	-0.500000
bq	0.000000	0.000000	-0.600000
bq	0.000000	0.000000	-0.700000
bq	0.000000	0.000000	-0.800000
bq	0.000000	0.000000	-0.900000
bq	0.000000	0.000000	-1.000000
bq	0.000000	0.000000	-1.100000
bq	0.000000	0.000000	-1.200000
bq	0.000000	0.000000	-1.300000
bq	0.000000	0.000000	-1.400000
bq	0.000000	0.000000	-1.500000
bq	0.000000	0.000000	-1.600000
bq	0.000000	0.000000	-1.700000
bq	0.000000	0.000000	-1.800000
bq	0.000000	0.000000	-1.900000
bq	0.000000	0.000000	-2.000000
bq	0.000000	0.000000	-2.100000
bq	0.000000	0.000000	-2.200000
bq	0.000000	0.000000	-2.300000
bq	0.000000	0.000000	-2.400000
bq	0.000000	0.000000	-2.500000
bq	0.000000	0.000000	-2.600000
bq	0.000000	0.000000	-2.700000
bq	0.000000	0.000000	-2.800000
bq	0.000000	0.000000	-2.900000
bq	0.000000	0.000000	-3.000000
bq	0.000000	0.000000	-3.100000
bq	0.000000	0.000000	-3.200000
bq	0.000000	0.000000	-3.300000
bq	0.000000	0.000000	-3.400000
bq	0.000000	0.000000	-3.500000
bq	0.000000	0.000000	-3.600000
bq	0.000000	0.000000	-3.700000
bq	0.000000	0.000000	-3.800000
bq	0.000000	0.000000	-3.900000
bq	0.000000	0.000000	-4.000000
bq	0.000000	0.000000	-4.100000
bq	0.000000	0.000000	-4.200000
bq	0.000000	0.000000	-4.300000
bq	0.000000	0.000000	-4.400000
bq	0.000000	0.000000	-4.500000
bq	0.000000	0.000000	-4.600000
bq	0.000000	0.000000	-4.700000
bq	0.000000	0.000000	-4.800000
bq	0.000000	0.000000	-4.900000

$^{Th6}CDH_{Screw}$: minimum energy in T_1 state

Symmetry: C_1

Energy: -3546.89784334

C	1.190140	-1.451922	-1.142155
C	0.080916	-0.689503	-1.485740
C	0.966206	-2.851785	-1.377403
H	1.697356	-3.611199	-1.120856
C	-0.274380	-3.158109	-1.860254
S	-1.215649	-1.707376	-2.095713
C	-1.147433	1.532273	-1.033229
C	-0.075120	0.745540	-1.459495
C	-0.893751	2.934695	-1.253539
H	-1.584899	3.708142	-0.936144
C	0.327219	3.215955	-1.786811
S	1.217843	1.745698	-2.102801
C	-2.389655	1.092459	-0.424190
C	-2.607831	-0.062130	0.448927
C	-3.614019	1.712461	-0.760491
H	-3.661597	2.586330	-1.401925
C	-4.754007	1.068510	-0.335628
S	-4.368464	-0.391395	0.571812
C	-0.311074	-0.663094	1.434062
C	-1.735225	-0.849763	1.157978
C	0.299465	-1.874463	1.832011
H	1.361669	-1.949716	2.033033
C	-0.557665	-2.926381	2.027886
S	-2.238803	-2.485493	1.699933
C	0.338989	0.618046	1.482912
C	1.680129	0.845470	1.151445
C	-0.309651	1.806444	1.974100
H	-1.350278	1.815068	2.276473
C	0.501210	2.894124	2.067596
S	2.119699	2.518917	1.507627
C	2.438029	-0.985619	-0.521956
C	2.614767	-0.007661	0.463553
C	3.688765	-1.574473	-0.914627
H	3.749823	-2.337539	-1.683747
C	4.795028	-1.058155	-0.305196
S	4.332198	0.175621	0.841190
C	-0.862232	-4.511020	-2.115382
H	-0.107790	-5.283143	-1.934129
H	-1.215366	-4.620585	-3.147890
H	-1.715043	-4.706694	-1.453597
C	6.231715	-1.415124	-0.525764
H	6.815276	-0.555289	-0.877053
H	6.304986	-2.203573	-1.281167
H	6.709078	-1.781082	0.391702
C	-0.224346	-4.311040	2.468165
H	0.836830	-4.378767	2.725820
H	-0.427908	-5.040485	1.672296
H	-0.813623	-4.613281	3.343285
C	-6.174487	1.440071	-0.598722
H	-6.220407	2.350310	-1.204286
H	-6.726109	1.622568	0.333084
H	-6.705991	0.644025	-1.136621
C	0.950612	4.556263	-2.017760
H	1.802718	4.715081	-1.344795
H	0.217038	5.346515	-1.829410
H	1.315886	4.670417	-3.045219
C	0.158413	4.267957	2.550033
H	0.805985	4.588113	3.375070
H	-0.877065	4.288277	2.903384
H	0.255886	5.010792	1.747969

$^{Th6}CDH_{Screw}$: transition state in T_1 state

Symmetry: C_1

Energy: -3546.85906072

C	0.896230	1.899210	0.877290
C	1.675110	1.160650	-0.004680
C	1.724660	2.559550	1.862130
H	1.331370	3.215290	2.627050
C	3.063750	2.330000	1.761650
S	3.383420	1.274080	0.423950
C	1.518700	-1.109290	-1.122380
C	1.459500	0.277480	-1.157190
C	1.743180	-1.687520	-2.415590
H	1.777780	-2.759150	-2.582970
C	1.860170	-0.770690	-3.421810
S	1.712570	0.852760	-2.796540
C	1.525700	-1.856590	0.120750
C	0.579710	-1.707850	1.233300
C	2.598710	-2.692990	0.470440
H	3.378220	-2.958110	-0.237090
C	2.683530	-3.027460	1.808520
S	1.349370	-2.336340	2.737240
C	-1.716220	-1.272120	0.115580
C	-0.773990	-1.486410	1.214910
C	-3.028110	-1.599170	0.496030
H	-3.859280	-1.523090	-0.198050
C	-3.220280	-1.888220	1.834650
S	-1.706900	-1.816640	2.724620
C	-1.542540	-0.524240	-1.146520
C	-1.504070	0.855070	-1.139390
C	-1.679840	-1.055050	-2.469410
H	-1.695390	-2.118730	-2.680830
C	-1.772470	-0.097860	-3.444700
S	-1.704190	1.503390	-2.751340
C	-0.570210	2.075420	0.954410
C	-1.547910	1.688680	0.053940
C	-1.162410	2.752670	2.086660
H	-0.595590	3.118470	2.932080
C	-2.518310	2.886180	2.046550
S	-3.149540	2.167640	0.595130
C	4.159600	2.872280	2.626750
H	3.736880	3.524290	3.397500
H	4.883460	3.457980	2.047590
H	4.712970	2.070260	3.129820
C	-3.410960	3.538080	3.057180
H	-3.947090	4.396020	2.633690
H	-2.815080	3.896710	3.902170
H	-4.162180	2.840410	3.446200
C	-4.504510	-2.168020	2.539290
H	-5.344730	-2.070990	1.844900
H	-4.665420	-1.469540	3.371300
H	-4.527670	-3.181700	2.961530
C	3.756940	-3.799400	2.497440
H	4.545140	-4.068560	1.787510
H	3.370750	-4.725710	2.943860
H	4.211640	-3.217090	3.310220
C	2.020040	-1.026340	-4.888570
H	1.130960	-0.722390	-5.453800
H	2.178230	-2.095220	-5.062750
H	2.876120	-0.484660	-5.307640
C	-1.892160	-0.295690	-4.924380
H	-1.087460	0.212500	-5.468430
H	-2.842480	0.090260	-5.313510
H	-1.839200	-1.362780	-5.161150

Bq atom coordinates for NICS scan:

bq	0.000000	0.000000	0.000000
bq	0.000000	0.000000	0.100000
bq	0.000000	0.000000	0.200000
bq	0.000000	0.000000	0.300000
bq	0.000000	0.000000	0.400000
bq	0.000000	0.000000	0.500000
bq	0.000000	0.000000	0.600000
bq	0.000000	0.000000	0.700000
bq	0.000000	0.000000	0.800000
bq	0.000000	0.000000	0.900000
bq	0.000000	0.000000	1.000000
bq	0.000000	0.000000	1.100000
bq	0.000000	0.000000	1.200000
bq	0.000000	0.000000	1.300000
bq	0.000000	0.000000	1.400000
bq	0.000000	0.000000	1.500000
bq	0.000000	0.000000	1.600000
bq	0.000000	0.000000	1.700000
bq	0.000000	0.000000	1.800000
bq	0.000000	0.000000	1.900000
bq	0.000000	0.000000	2.000000
bq	0.000000	0.000000	2.100000
bq	0.000000	0.000000	2.200000
bq	0.000000	0.000000	2.300000
bq	0.000000	0.000000	2.400000
bq	0.000000	0.000000	2.500000
bq	0.000000	0.000000	2.600000
bq	0.000000	0.000000	2.700000
bq	0.000000	0.000000	2.800000
bq	0.000000	0.000000	2.900000
bq	0.000000	0.000000	3.000000
bq	0.000000	0.000000	3.100000
bq	0.000000	0.000000	3.200000
bq	0.000000	0.000000	3.300000
bq	0.000000	0.000000	3.400000
bq	0.000000	0.000000	3.500000
bq	0.000000	0.000000	3.600000
bq	0.000000	0.000000	3.700000
bq	0.000000	0.000000	3.800000
bq	0.000000	0.000000	3.900000
bq	0.000000	0.000000	4.000000
bq	0.000000	0.000000	4.100000
bq	0.000000	0.000000	4.200000
bq	0.000000	0.000000	4.300000
bq	0.000000	0.000000	4.400000

bq	0.000000	0.000000	4.500000
bq	0.000000	0.000000	4.600000
bq	0.000000	0.000000	4.700000
bq	0.000000	0.000000	4.800000
bq	0.000000	0.000000	4.900000

$^{Th6}CDH_{Screw}$: minimum energy in S_1 state

Symmetry: C_2

Energy: -3546.95377310

C	-1.322874	1.407466	-1.165761
C	-0.115996	0.696540	-1.412192
C	-1.192599	2.795151	-1.527293
H	-1.989692	3.515867	-1.385601
C	0.055966	3.168331	-1.928163
S	1.155580	1.800483	-1.954232
C	1.322993	-1.407426	-1.165727
C	0.116127	-0.696497	-1.412189
C	1.192723	-2.795104	-1.527285
H	1.989814	-3.515823	-1.385595
C	-0.055839	-3.168281	-1.928173
S	-1.155477	-1.800451	-1.954135
C	2.507941	-0.915696	-0.513210
C	2.637176	0.122929	0.464872
C	3.794983	-1.470035	-0.847459
H	3.910733	-2.261053	-1.579547
C	4.868027	-0.858794	-0.272585
S	4.354852	0.430121	0.802331
C	0.306548	0.647871	1.413941
C	1.681020	0.900134	1.158726
C	-0.383816	1.834305	1.833242
H	-1.445022	1.841652	2.049293
C	0.413630	2.923847	2.017222
S	2.087737	2.570597	1.622848
C	-0.306643	-0.647954	1.413918
C	-1.681096	-0.900201	1.158586
C	0.383653	-1.834383	1.833339
H	1.444828	-1.841734	2.049534
C	-0.413827	-2.923915	2.017236
S	-2.087868	-2.570696	1.622555
C	-2.507859	0.915728	-0.513322
C	-2.637183	-0.122949	0.464697
C	-3.794870	1.470108	-0.847624
H	-3.910558	2.261169	-1.579675
C	-4.867963	0.858860	-0.272850
S	-4.354886	-0.430133	0.802018
C	0.557817	4.542515	-2.237156
H	-0.263057	5.262567	-2.174245
H	0.988101	4.604122	-3.243609
H	1.337836	4.846913	-1.528338
C	-6.321065	1.142359	-0.481324
H	-6.846233	0.269973	-0.888250
H	-6.440964	1.971395	-1.184186
H	-6.819057	1.415670	0.456548
C	0.020311	4.280472	2.505761
H	-1.046560	4.292775	2.744711
H	0.207598	5.048540	1.745525
H	0.578303	4.565278	3.405301
C	6.321150	-1.142245	-0.480984
H	6.441114	-1.971237	-1.183887
H	6.819089	-1.415599	0.456903
H	6.846323	-0.269824	-0.887826
C	-0.557680	-4.542461	-2.237199
H	-1.337676	-4.846892	-1.528367
H	0.263205	-5.262504	-2.174332
H	-0.987992	-4.604043	-3.243641
C	-0.020577	-4.280535	2.505839
H	-0.578727	-4.565358	3.405275
H	1.046251	-4.292825	2.744979
H	-0.207718	-5.048596	1.745559

$^{Th6}CDH_{Screw}$: transition state in S_1 state

Symmetry: C_s

Energy: -3546.90235796

C	0.723210	-0.928080	1.680049
C	1.592160	0.179965	1.439843
C	1.440805	-1.973394	2.377849
H	1.008376	-2.935529	2.617711
C	2.692528	-1.648226	2.801789
S	3.125717	-0.018777	2.319782
C	1.348734	1.093290	-0.880248
C	1.502530	1.252836	0.516137
C	1.481070	2.327356	-1.577266
H	1.408220	2.402788	-2.656536
C	1.752078	3.406034	-0.777126
S	1.877499	2.932988	0.918225
C	1.609173	-0.228506	-1.481603
C	0.720936	-1.109017	-2.087358
C	2.952551	-0.737795	-1.459133
H	3.769072	-0.187149	-1.005857
C	3.118324	-1.944681	-2.076373
S	1.585609	-2.531139	-2.682658
C	-1.608568	-0.229172	-1.482055
C	-0.719807	-1.109342	-2.087532
C	-2.951728	-0.739035	-1.459844
H	-3.768594	-0.188703	-1.006812
C	-3.116836	-1.946039	-2.077035
S	-1.583723	-2.531894	-2.682909
C	-1.348774	1.092792	-0.880816
C	-1.503067	1.252517	0.515499
C	-1.481252	2.326702	-1.578085
H	-1.408111	2.401954	-2.657349
C	-1.752824	3.405435	-0.778213
S	-1.878619	2.932630	0.917184
C	-0.723727	-0.928165	1.679839
C	-1.592739	0.179772	1.439339
C	-1.441392	-1.973527	2.377494
H	-1.008921	-2.935606	2.617505
C	-2.693261	-1.648479	2.801093
S	-3.126505	-0.019091	2.318927
C	3.641543	-2.483015	3.601815

H	3.189966	-3.454878	3.819795
H	3.893547	-2.000966	4.553745
H	4.580579	-2.656885	3.063476
C	-3.642402	-2.483343	3.600892
H	-3.894843	-2.001210	4.552664
H	-3.190722	-3.455092	3.819169
H	-4.581212	-2.657464	3.062238
C	-4.378572	-2.722842	-2.288064
H	-5.217613	-2.187944	-1.834372
H	-4.324148	-3.717908	-1.831215
H	-4.600090	-2.860533	-3.353079
C	4.380443	-2.720925	-2.287165
H	5.219125	-2.185771	-1.833111
H	4.602346	-2.858318	-3.352139
H	4.326286	-3.716102	-1.830528
C	1.953162	4.829684	-1.180704
H	1.192215	5.487250	-0.741494
H	1.888304	4.920141	-2.268775
H	2.931803	5.208384	-0.861472
C	-1.954216	4.828962	-1.182068
H	-1.193461	5.486778	-0.742905
H	-2.932975	5.207487	-0.862991
H	-1.889285	4.919246	-2.270150

$^{Th2}COT_{Saddle}$: minimum energy in S_0 state

Symmetry: C_2

Energy: -1337.12185258

C	-0.001450	1.735360	-0.557100
C	-1.244020	1.139080	-0.573290
C	0.001450	2.969890	0.177560
H	0.891180	3.580960	0.295260
C	-1.202940	3.307830	0.725570
S	-2.405080	2.104730	0.325190
C	1.195870	1.274500	-1.274530
C	1.734910	0.045100	-1.278130
C	0.001450	-1.735360	-0.557100
C	1.244020	-1.139080	-0.573290
C	-0.001450	-2.969890	0.177560
H	-0.891180	-3.580960	0.295260
C	1.202940	-3.307830	0.725570
S	2.405080	-2.104730	0.325190
C	-1.195870	-1.274500	-1.274530
C	-1.734910	-0.045100	-1.278130
C	1.540780	-4.495900	1.572140
H	0.667170	-5.149550	1.656000
H	1.839970	-4.202430	2.585750
H	2.362650	-5.082210	1.144110
C	-1.540780	4.495900	1.572140
H	-2.362650	5.082210	1.144110
H	-0.667170	5.149550	1.656000
H	-1.839970	4.202430	2.585750
H	2.671280	-0.087170	-1.819550
H	1.732590	2.052010	-1.817960
H	-2.671280	0.087170	-1.819550
H	-1.732590	-2.052010	-1.817960

$^{Th2}COT_{Saddle}$: transition state in S_0 state

Symmetry: C_{2h}

Energy: -1337.11263182

C	1.734920	0.640560	0.000000
C	1.698040	-0.705670	0.000000
C	0.744430	1.721680	0.000000
C	-0.642600	1.710310	0.000000
C	1.260920	3.061050	0.000000
H	2.327070	3.267840	0.000000
C	0.324600	4.058390	0.000000
S	-1.260920	3.356770	0.000000
C	-1.734920	-0.640560	0.000000
C	-1.698040	0.705670	0.000000
C	-0.744430	-1.721680	0.000000
C	0.642600	-1.710310	0.000000
C	-1.260920	-3.061050	0.000000
H	-2.327070	-3.267840	0.000000
C	-0.324600	-4.058390	0.000000
S	1.260920	-3.356770	0.000000
C	0.539880	5.540040	0.000000
H	1.612400	5.757000	0.000000
H	0.099910	6.016700	0.884060
H	0.099910	6.016700	-0.884060
C	-0.539880	-5.540040	0.000000
H	-0.099910	-6.016700	-0.884060
H	-1.612400	-5.757000	0.000000
H	-0.099910	-6.016700	0.884060
H	2.687730	-1.159990	0.000000
H	2.747040	1.040450	0.000000
H	-2.687730	1.159990	0.000000
H	-2.747040	-1.040450	0.000000

$^{Th2}COT_{Saddle}$: minimum energy in T_1 state

Symmetry: C_{2h}

Energy: -1337.08718020

C	0.017200	1.878870	0.000220
C	-1.298840	1.297070	-0.000410
C	-0.060980	3.317950	0.000540
H	0.832710	3.934930	0.001140
C	-1.309640	3.854220	0.000260
S	-2.526160	2.590080	-0.000690
C	1.298840	1.294590	-0.000570
C	1.824250	-0.002040	-0.000450
C	-0.017200	-1.878870	0.000220
C	1.298840	-1.297070	-0.000410
C	0.060980	-3.317950	0.000540
H	-0.832710	-3.934930	0.001140
C	1.309640	-3.854220	0.000260
S	2.526160	-2.590080	-0.000690
C	-1.298840	-1.294590	-0.000570
C	-1.824250	0.002040	-0.000450
C	1.701470	-5.296440	0.001390
H	0.807780	-5.927580	-0.002290
H	2.296350	-5.552300	0.887100

H	2.303380	-5.551890	-0.879620
C	-1.701470	5.296440	0.001390
H	-2.303380	5.551890	-0.879620
H	-0.807780	5.927580	-0.002290
H	-2.296350	5.552300	0.887100
H	2.913030	0.008090	-0.000510
H	2.084740	2.047200	-0.000670
H	-2.913030	-0.008090	-0.000510
H	-2.084740	-2.047200	-0.000670

$^{Th2}COT_{Saddle}$: minimum energy in S_1 state
Symmetry: C_{2h}
Energy: -1337.10692141

C	0.772920	1.714310	-0.000070
C	-0.673940	1.710570	-0.000160
C	1.276190	3.068260	0.000220
H	2.341030	3.278130	0.000440
C	0.343740	4.057900	0.000220
S	-1.276190	3.386510	-0.000020
C	1.707930	0.669560	-0.000410
C	1.669480	-0.736190	-0.000410
C	-0.772920	-1.714310	-0.000070
C	0.673940	-1.710570	-0.000160
C	-1.276190	-3.068260	0.000220
H	-2.341030	-3.278130	0.000440
C	-0.343740	-4.057900	0.000220
S	1.276190	-3.386510	-0.000020
C	-1.707930	-0.669560	-0.000410
C	-1.669480	0.736190	-0.000410
C	-0.558020	-5.537120	0.000500
H	-1.628530	-5.759150	0.000320
H	-0.110810	-6.007480	0.884210
H	-0.110440	-6.007910	-0.882790
C	0.558020	5.537120	0.000500
H	0.110440	6.007910	-0.882790
H	1.628530	5.759150	0.000320
H	0.110810	6.007480	0.884210
H	2.672290	-1.159730	-0.000610
H	2.730370	1.040630	-0.000560
H	-2.672290	1.159730	-0.000610
H	-2.730370	-1.040630	-0.000560

[12]Annulene: minimum energy in T_1 state
Symmetry: C_2
Energy: -464.393683485

C	0.452380	-0.557980	-2.533000
C	-0.452380	0.557980	-2.533000
C	-1.183160	1.139850	-1.508670
C	-1.382670	0.657740	-0.192960
C	-1.418330	-0.682250	0.199270
C	-1.183160	-1.153590	1.490890
C	-0.469390	-0.519730	2.539490
C	0.469390	0.519730	2.539490
C	1.183160	1.153590	1.490890
C	1.418330	0.682250	0.199270
C	1.382670	-0.657740	-0.192960
C	1.183160	-1.139850	-1.508670
H	0.573130	-1.025480	-3.511090
H	-0.573130	1.025480	-3.511090
H	1.656420	1.418850	-0.566460
H	1.606240	-2.120030	-1.731610
H	1.526170	-1.404560	0.586460
H	1.554020	2.152490	1.722280
H	0.677610	0.925790	3.530330
H	-0.677610	-0.925790	3.530330
H	-1.554020	-2.152490	1.722280
H	-1.656420	-1.418850	-0.566460
H	-1.526170	1.404560	0.586460
H	-1.606240	2.120030	-1.731610

[12]Annulene: higher-order saddle point in T_1 state
Symmetry: D_{12h}
Energy: -464.240937102

C	0.000000	0.000000	2.724070
C	0.000000	1.362040	2.359180
C	0.000000	2.359100	1.362030
C	0.000000	2.724040	-0.000000
C	0.000000	2.359100	-1.362030
C	0.000000	1.362040	-2.359180
C	0.000000	0.000000	-2.724070
C	-0.000000	1.362040	-2.359180
C	-0.000000	2.359100	-1.362030
C	-0.000000	2.724040	0.000000
C	-0.000000	2.359100	1.362030
C	-0.000000	1.362040	2.359180
H	0.000000	0.000000	3.812860
H	-0.000000	1.906520	3.302190
H	-0.000000	3.302030	1.906430
H	-0.000000	3.812940	0.000000
H	0.000000	3.302030	-1.906430
H	0.000000	1.906520	-3.302190
H	0.000000	0.000000	-3.812860
H	0.000000	1.906520	-3.302190
H	0.000000	3.302030	-1.906430
H	0.000000	3.812940	-0.000000
H	0.000000	3.302030	1.906430
H	0.000000	1.906520	3.302190

(Optimized at B3LYP-D3(BJ)/6-311+G(d,p))
Fluorenone: minimum energy in S_0 state
Symmetry: C_{2v}
Energy: -575.630658299

C	0.000000	3.027760	-1.393490
C	0.000000	3.465070	-0.066250
C	0.000000	2.537220	0.983410
C	0.000000	1.188760	0.665980
C	0.000000	0.741140	-0.670570
C	0.000000	1.661140	-1.710490
H	0.000000	3.758990	-2.196950

H	0.000000	4.529190	0.149970
H	0.000000	2.852620	2.022620
H	0.000000	1.339750	-2.748220
C	-0.000000	-1.188760	0.665980
C	-0.000000	-2.537220	0.983410
C	-0.000000	-3.465070	-0.066250
C	-0.000000	-3.027760	-1.393490
C	-0.000000	-1.661140	-1.710490
C	-0.000000	-0.741140	-0.670570
H	-0.000000	-2.852620	2.022620
H	-0.000000	-4.529190	0.149970
H	-0.000000	-3.758990	-2.196950
H	-0.000000	-1.339750	-2.748220
C	0.000000	-0.000000	1.578420
O	0.000000	-0.000000	2.796450

(Optimized at B3LYP-D3(BJ)/6-311+G(d,p))

Fluorenone: minimum energy in T_1 state

Symmetry: C_{2v}

Energy: -575.546777465

C	0.000000	2.991310	-1.427910
C	0.000000	3.426550	-0.052750
C	0.000000	2.518870	0.995320
C	0.000000	1.155770	0.700270
C	0.000000	0.699430	-0.695310
C	0.000000	1.660550	-1.749630
H	0.000000	3.742300	-2.211790
H	0.000000	4.492860	0.153850
H	0.000000	2.843520	2.031370
H	0.000000	1.337390	-2.786730
C	-0.000000	-1.155770	0.700270
C	-0.000000	-2.518870	0.995320
C	-0.000000	-3.426550	-0.052750
C	-0.000000	-2.991310	-1.427910
C	-0.000000	-1.660550	-1.749630
C	-0.000000	-0.699430	-0.695310
H	-0.000000	-2.843520	2.031370
H	-0.000000	-4.492860	0.153850
H	-0.000000	-3.742300	-2.211790
H	-0.000000	-1.337390	-2.786730
C	0.000000	-0.000000	1.602780
O	0.000000	-0.000000	2.846250

$^{Th4}COT_{Saddle}$: Saddle point in the S_0 state

Number of imaginary frequencies: 3

Symmetry: D_{2h}

Energy: -2364.58552467

C	-0.74466	1.76275	0.00000
C	-1.68653	0.73484	0.00000
C	-1.39740	3.05552	0.00000
H	-0.87624	3.99890	0.00000
C	-2.75528	3.05202	0.00000
S	-3.32741	1.42714	0.00000
C	0.74466	1.76275	0.00000
C	1.68653	0.73484	0.00000
C	1.39740	3.05552	0.00000
H	0.87624	3.99890	0.00000
C	2.75528	3.05202	0.00000
S	3.32741	1.42714	0.00000
C	0.74466	-1.76275	0.00000
C	1.68653	-0.73484	0.00000
C	1.39740	-3.05552	0.00000
H	0.87624	-3.99890	0.00000
C	2.75528	-3.05202	0.00000
S	3.32741	-1.42714	0.00000
C	-0.74466	-1.76275	-0.00000
C	-1.68653	-0.73484	-0.00000
C	-1.39740	-3.05552	-0.00000
H	-0.87624	-3.99890	-0.00000
C	-2.75528	-3.0520	-0.00000
S	-3.32741	-1.42714	-0.00000
C	3.68980	-4.22091	0.00000
H	3.11657	-5.15304	0.00000
H	4.33908	-4.22318	0.88385
H	4.33908	-4.22318	-0.88385
C	3.68980	4.22091	0.00000
H	3.11657	5.15304	0.00000
H	4.33908	4.22318	-0.88385
H	4.33908	4.22318	0.88385
C	-3.68980	4.22091	0.00000
H	-4.33908	4.22318	-0.88385
H	-3.11657	5.15304	0.00000
H	-4.33908	4.22318	0.88385
C	-3.68980	-4.22091	-0.00000
H	-4.33908	-4.22318	0.88385
H	-3.11657	-5.15304	-0.00000
H	-4.33908	-4.22318	-0.88385

$^{Th4}COT_{Saddle}$: Saddle point in the T_1 state

Number of imaginary frequencies: 5

Symmetry: D_{2h}

Energy: -2364.55145171

C	-0.71730	1.77324	0.00000
C	-1.68910	0.70561	0.00000
C	-1.39285	3.05614	0.00000
H	-0.87992	4.00411	0.00000
C	-2.74568	3.04436	0.00000
S	-3.34504	1.41397	0.00000
C	0.71730	1.77324	0.00000
C	1.68910	0.70561	0.00000
C	1.39285	3.05614	0.00000
H	0.87992	4.00411	0.00000
C	2.74568	3.04436	0.00000
S	3.34504	1.41397	0.00000
C	0.71730	-1.77324	0.00000
C	1.68910	-0.70561	0.00000
C	1.39285	-3.05614	0.00000
H	0.87992	-4.00411	0.00000
C	2.74568	-3.04436	0.00000
S	3.34504	-1.41397	0.00000

C	-0.71730	-1.77324	-0.00000
C	-1.68910	-0.70561	-0.00000
C	-1.39285	-3.05614	-0.00000
H	-0.87992	-4.00411	-0.00000
C	-2.74568	-3.04436	-0.00000
S	-3.34504	-1.41397	-0.00000
C	3.68275	-4.20842	0.00000
H	3.11739	-5.14520	0.00000
H	4.33337	-4.20185	0.88323
H	4.33337	-4.20185	-0.88323
C	3.68275	4.20842	0.00000
H	3.11739	5.14520	0.00000
H	4.33337	4.20185	-0.88323
H	4.33337	4.20185	0.88323
C	-3.68275	4.20842	0.00000
H	-4.33337	4.20185	-0.88323
H	-3.11739	5.14520	0.00000
H	-4.33337	4.20185	0.88323
C	-3.68275	-4.20842	-0.00000
H	-4.33337	-4.20185	0.88323
H	-3.11739	-5.14520	-0.00000
H	-4.33337	-4.20185	-0.88323

^{Th4}COT$_{Saddle}$: Saddle point in the T_1 state

Number of imaginary frequencies: 3

Symmetry: C_{2v}

Energy: -2364.55157135

C	-0.13054	0.71678	1.76246
C	0.05338	1.68084	0.70501
C	-0.23367	1.39641	3.03863
H	-0.41769	0.89076	3.97318
C	-0.06350	2.73904	3.03790
S	0.21560	3.32310	1.42378
C	-0.13056	-0.71678	1.76246
C	0.05342	-1.68083	0.70500
C	-0.23377	-1.39643	3.03861
H	-0.41791	-0.89078	3.97315
C	-0.06354	-2.73904	3.03789
S	0.21569	-3.32309	1.42378
C	-0.13056	-0.71678	-1.76246
C	0.05342	-1.68083	-0.70500
C	-0.23377	-1.39643	-3.03861
H	-0.41791	-0.89078	-3.97315
C	-0.06354	-2.73904	-3.03789
S	0.21569	-3.32309	-1.42378
C	-0.13054	0.71678	-1.76246
C	0.05338	1.68084	-0.70501
C	-0.23367	1.39641	-3.03863
H	-0.41769	0.89076	-3.97318
C	-0.06350	2.73904	-3.03790
S	0.21560	3.32310	-1.42378
C	-0.08556	-3.67605	-4.20171
H	-0.27148	-3.12149	-5.12646
H	-0.87034	-4.43513	-4.09441
H	0.86843	-4.20733	-4.30841
C	-0.08556	-3.67605	4.20171
H	-0.27148	-3.12149	5.12646
H	0.86843	-4.20733	4.30841
H	-0.87034	-4.43513	4.09441
C	-0.08546	3.67604	4.20173
H	0.86851	4.20737	4.30835
H	-0.27127	3.12146	5.12650
H	-0.87029	4.43508	4.09451
C	-0.08546	3.67604	-4.20173
H	-0.87029	4.43508	-4.09451
H	-0.27127	3.12146	-5.12650
H	0.86851	4.20737	-4.30835

^{Th4}COT$_{Saddle}$: Saddle point in the T_1 state

Number of imaginary frequencies: 2

Symmetry: C_{2h}

Energy: -2364.55225247

C	0.01986	0.71651	1.75780
C	-0.08693	1.69445	0.70422
C	0.15789	1.37892	3.03902
H	0.37190	0.84905	3.95509
C	0.07529	2.73036	3.04542
S	-0.16305	3.33864	1.43171
C	-0.01986	-0.71651	1.75780
C	0.08693	-1.69445	0.70422
C	-0.15789	-1.37892	3.03902
H	-0.37190	-0.84905	3.95509
C	-0.07529	-2.73036	3.04542
S	0.16305	-3.33864	1.43171
C	-0.01986	-0.71651	-1.75780
C	0.08693	-1.69445	-0.70422
C	-0.15789	-1.37892	-3.03902
H	-0.37190	-0.84905	-3.95509
C	-0.07529	-2.73036	-3.04542
S	0.16305	-3.33864	-1.43171
C	0.01986	0.71651	-1.75780
C	-0.08693	1.69445	-0.70422
C	0.15789	1.37892	-3.03902
H	0.37190	0.84905	-3.95509
C	0.07529	2.73036	-3.04542
S	-0.16305	3.33864	-1.43171
C	-0.18951	-3.66146	-4.20853
H	-0.34722	-3.09273	-5.12987
H	-1.02827	-4.35823	-4.08782
H	0.71896	-4.26424	-4.33172
C	-0.18951	-3.66146	4.20853
H	-0.34722	-3.09273	5.12987
H	0.71896	-4.26424	4.33172
H	-1.02827	-4.35823	4.08782
C	0.18951	3.66146	4.20853
H	1.02827	4.35823	4.08782
H	0.34722	3.09273	5.12987
H	-0.71896	4.26424	4.33172
C	0.18951	3.66146	-4.20853
H	-0.71896	4.26424	-4.33172

H	0.34722	3.09273	-5.12987
H	1.02827	4.35823	-4.08782

$^{Th4}COT_{Saddle}$: Saddle point in the S_1 state

Number of imaginary frequencies: 5

Symmetry: D_{2h}

Energy: -2364.58296275 (ultrafine integration grid)

C	-0.71501	1.77318	0.00000
C	-1.69222	0.70540	0.00000
C	-1.39085	3.05889	0.00000
H	-0.87749	4.00608	0.00000
C	-2.74461	3.04661	0.00000
S	-3.34407	1.41668	0.00000
C	0.71501	1.77318	0.00000
C	1.69222	0.70540	0.00000
C	1.39085	3.05889	0.00000
H	0.87749	4.00608	0.00000
C	2.74461	3.04661	0.00000
S	3.34407	1.41668	0.00000
C	0.71501	-1.77318	0.00000
C	1.69222	-0.70540	0.00000
C	1.39085	-3.05889	0.00000
H	0.87749	-4.00608	0.00000
C	2.74461	-3.04661	0.00000
S	3.34407	-1.41668	0.00000
C	-0.71501	-1.77318	-0.00000
C	-1.69222	-0.70540	-0.00000
C	-1.39085	-3.05889	-0.00000
H	-0.87749	-4.00608	-0.00000
C	-2.74461	-3.04661	-0.00000
S	-3.34407	-1.41668	-0.00000
C	3.68373	-4.20932	-0.00000
H	3.11861	-5.14538	-0.00000
H	4.33294	-4.20075	0.88336
H	4.33294	-4.20075	-0.88336
C	3.68373	4.20932	0.00000
H	3.11861	5.14538	0.00000
H	4.33294	4.20075	-0.88336
H	4.33294	4.20075	0.88336
C	-3.68373	4.20932	-0.00000
H	-4.33294	4.20075	-0.88336
H	-3.11861	5.14538	-0.00000
H	-4.33294	4.20075	0.88336
C	-3.68373	-4.20932	-0.00000
H	-4.33294	-4.20075	0.88336
H	-3.11861	-5.14538	-0.00000
H	-4.33294	-4.20075	-0.88336

$^{Th4}COT_{Saddle}$: Saddle point in the S_1 state

Number of imaginary frequencies: 2

Symmetry: C_{2h}

Energy: -2364.58352053 (ultrafine integration grid)

C	0.01918	0.71437	1.76031
C	-0.07788	1.69680	0.70436
C	0.14761	1.37978	3.04435
H	0.34405	0.85275	3.96510
C	0.07059	2.73219	3.04738
S	-0.15229	3.33830	1.43165
C	-0.01918	-0.71437	1.76031
C	0.07788	-1.69680	0.70436
C	-0.14761	-1.37978	3.04435
H	-0.34405	-0.85275	3.96510
C	-0.07059	-2.73219	3.04738
S	0.15229	-3.33830	1.43165
C	-0.01918	-0.71437	-1.76031
C	0.07788	-1.69680	-0.70436
C	-0.14761	-1.37978	-3.04435
H	-0.34405	-0.85275	-3.96510
C	-0.07059	-2.73219	-3.04738
S	0.15229	-3.33830	-1.43165
C	0.01918	0.71437	-1.76031
C	-0.07788	1.69680	-0.70436
C	0.14761	1.37978	-3.04435
H	0.34405	0.85275	-3.96510
C	0.07059	2.73219	-3.04738
S	-0.15229	3.33830	-1.43165
C	-0.17566	-3.66712	-4.20859
H	-0.32126	-3.10005	-5.13207
H	-1.01833	-4.35907	-4.09341
H	0.73201	-4.27238	-4.31878
C	-0.17566	-3.66712	4.20859
H	-0.32126	-3.10005	5.13207
H	0.73201	-4.27238	4.31878
H	-1.01833	-4.35907	4.09341
C	0.17566	3.66712	4.20859
H	1.01833	4.35907	4.09341
H	0.32126	3.10005	5.13207
H	-0.73201	4.27238	4.31878
C	0.17566	3.66712	-4.20859
H	-0.73201	4.27238	-4.31878
H	0.32126	3.10005	-5.13207
H	1.01833	4.35907	-4.09341

Supplementary References

1 Ditchfield, R., Hehre, W. J. & Pople, J. A. Self-consistent molecular-orbital methods. IX. An extended Gaussian-type basis for molecular-orbital studies of organic molecules. *J. Chem. Phys.* **54,** 724–728 (1971).

2 Krishnan, R., Binkley, J. S., Seeger, R. & Pople, J. A. Self-consistent molecular orbital methods. XX. A basis set for correlated wave functions. *J. Chem. Phys.* **72,** 650–650 (1980).

3 Gaussian 09, Revision E.01, Frisch, M. J., Trucks, G. W., Schlegel, H. B., Scuseria, G. E., Robb, M. A., Cheeseman, J. R., Scalmani, G., Barone, V., Mennucci, B., Petersson, G. A., Nakatsuji, H., Caricato, M., Li, X., Hratchian, H. P., Izmaylov, A. F., Bloino, J., Zheng, G., Sonnenberg, J. L., Hada, M., Ehara, M., Toyota, K., Fukuda, R., Hasegawa, J., Ishida, M., Nakajima, T., Honda, Y., Kitao, O., Nakai, H., Vreven, T., Montgomery, J. A. Jr., Peralta, J. E., Ogliaro, F., Bearpark, M., Heyd, J. J., Brothers, E., Kudin, K. N., Staroverov, V. N., Kobayashi, R., Normand, J., Raghavachari, K., Rendell, A., Burant, J. C., Iyengar, S. S., Tomasi, J., Cossi, M., Rega, N., Millam, J. M., Klene, M., Knox, J. E., Cross, J. B., Bakken, V., Adamo, C., Jaramillo, J., Gomperts, R., Stratmann, R. E., Yazyev, O., Austin, A. J., Cammi, R., Pomelli, C., Ochterski, J. W., Martin, R. L., Morokuma, K., Zakrzewski, V. G., Voth, G. A., Salvador, P., Dannenberg, J. J., Dapprich, S., Daniels, A. D., Farkas, Ö., Foresman, J. B., Ortiz, J. V., Cioslowski, J., & Fox, D. J. *Gaussian, Inc*., Wallingford CT (2009).

4 Zhao, Y. & Truhlar, D. The M06 suite of density functionals for main group thermochemistry, thermochemical kinetics, noncovalent interactions, excited states, and transition elements: Two new functionals and systematic testing of four M06-class functionals and 12 other functionals. *Theor. Chem. Acc.* **120,** 215–241 (2008).

5 Grimme, S. Semiempirical hybrid density functional with perturbative second-order correlation. *J. Chem. Phys.* **124,** 034108 (2006).

6 Gaussian 16, Revision A.03, Frisch, M. J.; Trucks, G. W.; Schlegel, H. B.; Scuseria, G. E.; Robb, M. A.; Cheeseman, J. R.; Scalmani, G.; Barone, V.; Petersson, G. A.; Nakatsuji, H.; Li, X.; Caricato, M.; Marenich, A. V.; Bloino, J.; Janesko, B. G.; Gomperts, R.; Mennucci, B.; Hratchian, H. P.; Ortiz, J. V.; Izmaylov, A. F.; Sonnenberg, J. L.; Williams-Young, D.; Ding, F.; Lipparini, F.; Egidi, F.; Goings, J.; Peng, B.; Petrone, A.; Henderson, T.; Ranasinghe, D.; Zakrzewski, V. G.; Gao, J.; Rega, N.; Zheng, G.; Liang, W.; Hada, M.; Ehara, M.; Toyota, K.; Fukuda, R.; Hasegawa, J.; Ishida, M.; Nakajima, T.; Honda, Y.;

Kitao, O.; Nakai, H.; Vreven, T.; Throssell, K.; Montgomery, J. A., Jr.; Peralta, J. E.; Ogliaro, F.; Bearpark, M. J.; Heyd, J. J.; Brothers, E. N.; Kudin, K. N.; Staroverov, V. N.; Keith, T. A.; Kobayashi, R.; Normand, J.; Raghavachari, K.; Rendell, A. P.; Burant, J. C.; Iyengar, S. S.; Tomasi, J.; Cossi, M.; Millam, J. M.; Klene, M.; Adamo, C.; Cammi, R.; Ochterski, J. W.; Martin, R. L.; Morokuma, K.; Farkas, O.; Foresman, J. B.; Fox, D. J. Gaussian, Inc., Wallingford CT, 2016.

7 Rahalkar, A.; Stanger, A. "Aroma", http://chemistry.technion.ac.il/members/amnon-stanger/

8 Stanger, A. Nucleus-independent chemical shifts (NICS): Distance dependence and revised criteria for aromaticity and antiaromaticity. *J. Org. Chem.* **71,** 883–893 (2006).

9 Stanger, A. Obtaining relative induced ring currents quantitatively from NICS. *J. Org. Chem.* **75,** 2281–2288 (2010).

10 Gershoni-Poranne, R. & Stanger, A. The NICS-*XY*-Scan: Identification of local and global ring currents in multi-ring systems. *Chem. Eur. J.* **20,** 5673–5688 (2014).

11 Wolinski, K., Hinton, J. F. & Pulay, P. Efficient implementation of the gauge-independent atomic orbital method for NMR chemical shift calculations. *J. Am. Chem. Soc.* **112,** 8251–8260 (1990).

12 Roos, B. O., Taylor, P. R. & Siegbahn, P. E. M. A complete active space SCF method (CASSCF) using a density matrix formulated super-CI approach. *Chem. Phys.* **48,** 157–173 (1980).

13 Aidas K., Angeli C., Bak K. L., Bakken V., Bast R., Boman L., *et al.* The Dalton quantum chemistry program system. *Wiley Interdiscip. Rev.: Comput. Mol. Sci.* **4,** 269–284 (2014).

14 Keith, T. A. & Bader, R. F. W. Calculation of magnetic response properties using a continuous set of gauge transformations. *Chem. Phys. Lett.* **210,** 223–231 (1993).

15 Riplinger, C. & Neese, F. An efficient and near linear scaling pair natural orbital based local coupled cluster method. *J. Chem. Phys.* **138,** 034106 (2013).

16 Riplinger, C., Sandhoefer, B., Hansen, A. & Neese, F. Natural triple excitations in local coupled cluster calculations with pair natural orbitals. *J. Chem. Phys.* **139**, 134101 (2013).

17 Neese, F. The ORCA program system. *Wiley Interdiscip. Rev.: Comput. Mol. Sci.* **2,** 73–78 (2012).

18 Dunning, T. H. Gaussian basis sets for use in correlated molecular calculations. I. The atoms boron through neon and hydrogen. *J. Chem. Phys.* **90,** 1007–1023 (1989).

19 Woon, D. E. & Dunning, T. H. Gaussian basis sets for use in correlated molecular

calculations. III. The atoms aluminum through argon. *J. Chem. Phys.* **98,** 1358–1371 (1993).

20 Liakos, D. G., Sparta, M., Kesharwani, M. K., Martin, J. M. L. & Neese, F. Exploring the accuracy limits of local pair natural orbital coupled-cluster theory. *J. Chem. Theory Comput.* **11,** 1525–1539 (2015).

21 Aquilante, F., Autschbach, J., Carlson, R. K., Chibotaru, L. F., Delcey, M. G., De Vico, L., *et al.,* Molcas 8: New capabilities for multiconfigurational quantum chemical calculations across the periodic table. *J. Comput. Chem.* **37,** 506–541 (2016).

22 Andersson, K., Malmqvist, P.-Å. & Roos, B. O. Second-order perturbation theory with a complete active space self-consistent field reference function. *J. Chem. Phys.* **96,** 1218–1226 (1992).

23 Finley, J., Malmqvist, P.-Å., Roos, B. O. & Serrano-Andrés, L. The multi-state CASPT2 method. *Chem. Phys. Lett.* **288,** 299–306 (1998).

24 Del Campo, J. M. & Köster, A. M. A hierarchical transition state search algorithm. *J. Chem. Phys.* **129,** 024107 (2008).

25 Kato, S., Lee, H. S., Gareyev, R., Wenthold, P. G., Lineberger, W. C., DePuy, C. H., *et al.,* Experimental and computational studies of the structures and energetics of cyclooctatetraene and its derivatives. *J. Am. Chem. Soc.* **119,** 7863–7864 (1997).

26 Garavelli, M., Bernardi, F., Cembran, A., Castaño, O., Frutos, L. M., Merchán, M., *et al.,* Cyclooctatetraene computational photo- and thermal chemistry: Cyclooctatetraene computational photo- and thermal chemistry: A reactivity model for conjugated hydrocarbons. *J. Am. Chem. Soc.* **124,** 13770–13789 (2002).

27 Turro, N. J., Ramamurthy, V. & Scaiano, J. C. *Principles of Molecular Photochemistry: An introduction* (University Science Books, 2009).

28 Herkstroeter, W. G., Lamola, A. A. & Hammond, G. S. Mechanisms of photochemical reactions in solution. XXVIII. Values of triplet excitation energies of selected sensitizers. *J. Am. Chem. Soc.* **86,** 4537–4540 (1964).

29 Biczók, L. & Bérces, T. Temperature dependence of the rate of photophysical process of fluorenone. *J. Phys. Chem.* **92,** 3842–3845 (1988).

30 Zwarich, R. & Bree, A. A study of the excited electronic states of 9-fluorenone. *J. Mol. Spectrosc.* **52,** 329–343 (1974).

31 Paquette, L. A., Wang, T. Z., Luo, J., Cottrell, C. E., Clough, A. E. & Anderson, L. B. Is pseudorotation the operational pathway for bond shifting within [8]annulenes? Probe of

planarization requirements by 1,3-annulation of the cyclooctatetraene ring. Kinetic analysis of racemization and 2-D NMR quantitation of π-bond alternation and ring inversion as a function of polymethylene chain length. *J. Am. Chem. Soc.* **112,** 239–253 (1990).

32 Wenthold, P. G., Hrovat, D. A., Borden, W. T. & Lineberger, W. C. Transition-state spectroscopy of cyclooctatetraene. *Science* **272,** 1456–1459 (1996).

33 Schild, A. & Paulus, B. Multireference calculations for ring inversion and double bond shifting in cyclooctatetraene. *J. Comput. Chem.* **34,** 1393–1397 (2013).

34 Sakamoto, Y. & Suzuki. T. Tetrabenzo[8]circulene: Aromatic saddles from negatively curved graphene. *J. Am. Chem. Soc.* **135,** 14074–14077 (2013).

35 Gu, X., Li, H., Shan, B., Liu, Z. & Miao, Q. Synthesis, structure, and properties of tetrabenzo[7]circulene. *Org. Lett.* **19,** 2246–2249 (2017).

36 Garavelli, M. *et al.* Cyclooctatetraene computational photo- and thermal chemistry: A reactivity model for conjugated hydrocarbons. *J. Am. Chem. Soc.* **124,** 13770–13789 (2002).
